# A State-of-the-Art of Exoskeletons in Line with the WHO’s Vision on Healthy Aging: From Rehabilitation of Intrinsic Capacities to Augmentation of Functional Abilities

**DOI:** 10.3390/s24072230

**Published:** 2024-03-30

**Authors:** Rebeca Alejandra Gavrila Laic, Mahyar Firouzi, Reinhard Claeys, Ivan Bautmans, Eva Swinnen, David Beckwée

**Affiliations:** 1 Rehabilitation Research, Department of Physiotherapy, Human Physiology and Anatomy, Vrije Universiteit Brussel, Laarbeeklaan 103, 1090 Jette, Belgium; rebecagavrila@gmail.com (R.A.G.L.); mahyar.firouzi@vub.be (M.F.); reinhard.claeys@vub.be (R.C.); david.beckwee@vub.be (D.B.); 2 Brain, Body and Cognition Research Group, Faculty of Psychology and Educational Sciences, Vrije Universiteit Brussel, Pleinlaan 2, 1050 Elsene, Belgium; 3 Center for Neurosciences (C4N), Vrije Universiteit Brussel, Pleinlaan 2, 1050 Elsene, Belgium; 4 Brubotics (Human Robotics Research Center), Vrije Universiteit Brussel, Pleinlaan 2, 1050 Elsene, Belgium; 5 FRIA, Frailty in Ageing, Vrije Universiteit Brussel, Laarbeeklaan 103, 1090 Brussels, Belgium; ivan.bautmans@vub.be

**Keywords:** exoskeletons, assistive technology, older adults, healthy aging, intrinsic capacity, functional ability

## Abstract

The global aging population faces significant health challenges, including an increasing vulnerability to disability due to natural aging processes. Wearable lower limb exoskeletons (LLEs) have emerged as a promising solution to enhance physical function in older individuals. This systematic review synthesizes the use of LLEs in alignment with the WHO’s healthy aging vision, examining their impact on intrinsic capacities and functional abilities. We conducted a comprehensive literature search in six databases, yielding 36 relevant articles covering older adults (65+) with various health conditions, including sarcopenia, stroke, Parkinson’s Disease, osteoarthritis, and more. The interventions, spanning one to forty sessions, utilized a range of LLE technologies such as Ekso^®^, HAL^®^, Stride Management Assist^®^, Honda Walking Assist^®^, Lokomat^®^, Walkbot^®^, Healbot^®^, Keeogo Rehab^®^, EX1^®^, overground wearable exoskeletons, Eksoband^®^, powered ankle–foot orthoses, HAL^®^ lumbar type, Human Body Posturizer^®^, Gait Enhancing and Motivation System^®^, soft robotic suits, and active pelvis orthoses. The findings revealed substantial positive outcomes across diverse health conditions. LLE training led to improvements in key performance indicators, such as the 10 Meter Walk Test, Five Times Sit-to-Stand test, Timed Up and Go test, and more. Additionally, enhancements were observed in gait quality, joint mobility, muscle strength, and balance. These improvements were accompanied by reductions in sedentary behavior, pain perception, muscle exertion, and metabolic cost while walking. While longer intervention durations can aid in the rehabilitation of intrinsic capacities, even the instantaneous augmentation of functional abilities can be observed in a single session. In summary, this review demonstrates consistent and significant enhancements in critical parameters across a broad spectrum of health conditions following LLE interventions in older adults. These findings underscore the potential of LLE in promoting healthy aging and enhancing the well-being of older adults.

## 1. Background

Healthy aging, as defined by the WHO, is the process of developing and maintaining the functional ability that enables well-being in older age [1]. This functional ability is intertwined with both individual intrinsic capacity (IC) and the surrounding environment in which individuals reside and interact. IC helps identify the focal points for medical assessments and treatments, while the domains related to functional ability determine the measure of its effectiveness. Within the domain of functional ability, the “ability to be mobile” is identified as a pivotal subdomain. It is a key factor for healthy aging, especially if we consider that 45% of adults who are more than 75 years old have at least one physical function difficulty. As physical losses occur with advancing age, older adults may face challenges in performing functional mobility tasks such as walking, standing up from a seated position, or climbing stairs [2]. When physical limitations hinder mobility, individuals are often inclined to avoid physical activity altogether. This behavior increases the risk of diseases associated with a sedentary lifestyle and can lead to withdrawal from participation in society, ultimately impacting their independence and quality of life [3]. The losses associated with declines in mobility extend beyond the individual: when older people are not able to move around, their social networks are affected, and the community may lose valuable contributions and might need additional resources to support older people in their daily lives. Facilitating the ability of older people to be able to get around when and how they choose, at an affordable cost, are important provisions of the United Nations Convention on the rights of persons with disabilities and optional protocol [4]. While traditional mobility aids (e.g., crutches, canes, and walkers) offer support by unloading joints, reducing pain, and improving balance, they come with limitations. For instance, they are often heavy, bulky, cumbersome, and restrict upper limb movement and functionality, hindering functional tasks requiring manual dexterity such as carrying objects, cooking, and using one’s hands freely while walking. Additionally, they may not adequately assist with essential functional activities like sit-to-stand transfers [3]. In this context, lower limb exoskeletons (LLEs) represent a potential game-changer for healthy aging, even if there is a significant gap in the understanding of the societal and rehabilitation implications of integrating LLEs into the lives of the older population.

IC, a key component of healthy aging, comprises physical and mental capacities, categorized into cognitive, psychological, locomotor, sensory, and vitality domains [5]. Evaluating IC in each of these areas necessitates rigorous ‘*stress tests*’ designed to measure maximum capability; thus, IC needs to be differentiated with ‘performance indicators’ within the same domain. For instance, to quantify *endurance* as an IC, the utilization of the 6 min walking test is considered a stress test since one is asked to walk ‘as far as possible’ in 6 min. Conversely, when executing a 10 meter-walking test at a self-paced walking speed, the outcomes primarily signify ‘a performance’ rather than an inherent capacity. Of particular importance for LLE use in older adults is locomotor capacity, encompassing musculoskeletal aspects crucial for endurance, balance, muscle strength, function, power, and joint functionality [6]. But ‘vitality capacity’ is also particularly important; for this capacity, self-perceived fatigue and muscle fatigability have been suggested as top biomarkers [7]. Most studies on LLE use in older adults lack this comprehensive assessment, highlighting a literature gap.

Aging-related physical changes that are related to the concept of IC often impact ‘functional abilities’ such as gait function and contribute to injuries in adults aged 65 and older [8,9]. These changes can lead to reduced physical activity, contributing to disability and psychosocial issues such as social isolation and depression [10,11]. Conversely, regular physical activity is associated with improved physical and cognitive functioning in older adults, a cornerstone of healthy aging [12]. Consequently, LLEs could be beneficial in older adults to bypass the decreased levels of IC, allowing for augmented functional ability [2]. In this context, LLEs have the potential to serve as an assistive technology, supporting older adults during their daily life activities.

Recent research underscores the significant benefits of exercise programs for older adults, slowing age-related changes and increasing life expectancy [13,14]. To address the challenges of healthy aging, the focus should be on IC rather than specific chronic diseases [15], as preserving physical performance, including muscle strength, power, and endurance, is essential for a healthy and productive life among the aging population and a key contributor to late-life mobility and independence [16].

In rehabilitation settings, LLEs have demonstrated potential for high-dosage, high-intensity gait training, complementing conventional exercise programs, while reducing strain on therapists [17]. Therefore, LLE training also has a great potential to rehabilitate IC and augment functional abilities.

As such, LLEs have shown promise in rehabilitating patients after stroke (e.g., enhancing walking speed and balance [18]) and spinal cord injury (e.g., enhancing walking speed, walking endurance, and bone mineral density [19]). Additionally, they have displayed potential in addressing age-related physical changes in functional ability of community-dwelling older adults (e.g., improved gait kinematics and kinetics, trunk and lower extremity muscle strength, and metabolic efficiency) [20]. Consequently, although most available exoskeletons are designed to augment human performance in industrial settings or aid in the rehabilitation of individuals with neurological conditions, their use is expected to expand beyond these contexts to assist older people in their functional abilities and to age in place [21].

Beyond the specific setting (e.g., industry, rehabilitation, assistive) in which they can be employed, LLEs can be categorized in various ways based on their functional attributes and design features. For instance, passive exoskeletons have no power sources, but rely on kinematic forces to ensure locomotion (e.g., using springs). Active exoskeletons, on the other hand, employ power sources to activate actuators (i.e., the devices responsible for generating motion in a specific part or joint within the exoskeleton). These actuators can drive a single joint (e.g., the Honda Walking Assist^®^ solely assists the hip joints), while some devices utilize multiple actuators to drive a combination of joints (e.g., the Ekso-GT^®^ [Ekso Bionics, San Rafael, CA, USA] assists both hip and knee joints) [22]. Furthermore, stationary exoskeletons (e.g., the treadmill-based Lokomat^®^ [Hocoma, Zürich, Switzerland] and Walkbot^®^ [Walkbot, Seoul, Republic of Korea]) offer a secure environment for repetitive training but are confined to rehabilitative institutions [18]. In contrast, wearable exoskeletons (e.g., Honda Walking Assist^®^ [Honda, Tokyo, Japan], Ekso-GT^®^ and Hybrid Assistive Limb^®^ [Cyberdyne, Tsukuba, Japan]) overcome this limitation, but may be less supportive and require a minimum trunk balance. With recent technological advancements, wearable exoskeletons designed to enhance physical functioning in aging populations [23] are becoming more affordable, lighter, and less constraining [24]. As outlined previously, these devices have demonstrated their potential in patients with neurological conditions, with robot-assisted rehabilitation programs showing positive impacts on gait-related outcomes in stroke and spinal cord injury [18,25,26], but also on quality of life and depressive symptoms in a range of neurological disorders [27].

We postulate that the potential of powered exoskeletons exceeds rehabilitation and industry settings, as they present a novel approach to enable older adults to engage in activities with greater ease and confidence. LLEs could be employed to compensate for diminished IC reserves (e.g., muscle strength, endurance, and movement speed) in healthy older adults, to aid in functional mobility tasks such as level walking, uphill walking, climbing stairs, and sit-to-stand transfers. Analogous to long-term effects reported in neurological populations, these improvements might extend beyond mere wear periods and can positively impact habitual physical activity and cardiorespiratory function and facilitate high-intensity activities like hiking and keeping pace with grandchildren.

Despite promising developments in treating neurological conditions, a comprehensive assessment of the effectiveness of robotic LLE-based interventions within a healthy aging context is lacking [28]. Therefore, this systematic review aims to provide an updated overview of LLE use for augmenting functional abilities as well as their impact on performance indicators and stress tests for IC domains in older adults, both with and without various health conditions.

## 2. Methods

### 2.1. Study Registration

**Figure 1:**
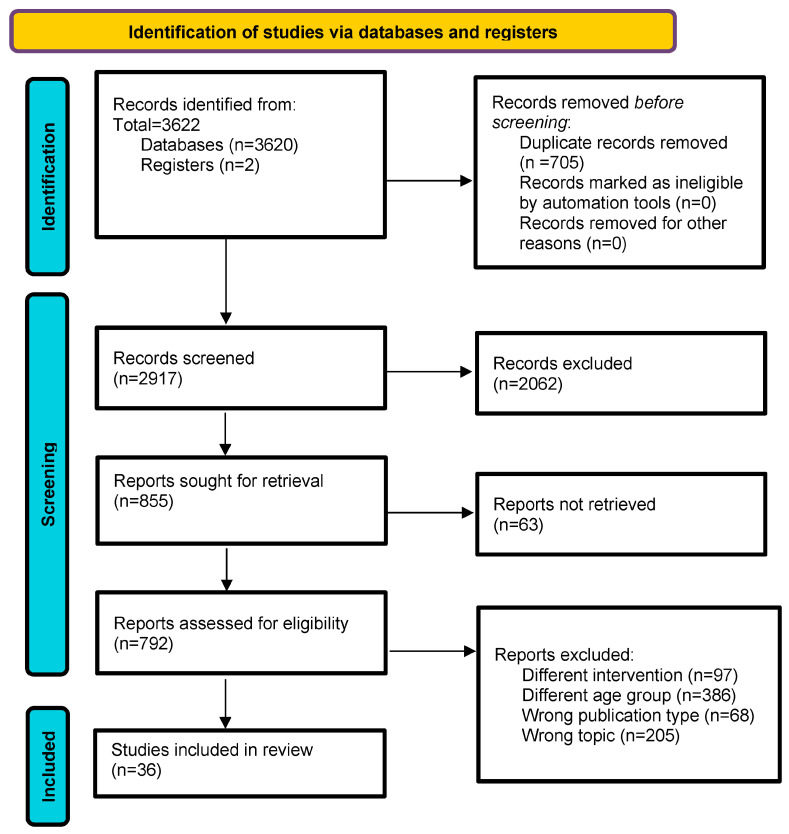
Prisma flow diagram.

The protocol for this systematic review was prospectively registered in the International Prospective Register of Systematic Reviews (PROSPERO, registration number [CRD42023434655]) in June 2023 and reported in accordance with the Preferred Reporting Items for Systematic Review and Meta-Analysis Protocols (PRISMA) 2020 statement [29] [Figure 1] and the Cochrane Handbook for Systematic Reviews of Interventions [30].

### 2.2. Search Strategy and Study Selection Criteria

In June 2023, a systematic search was conducted across various databases and registries, including PubMed, EMBASE, Web of Science (WOS), the Cochrane Central Register of Controlled Trials, CINAHL, PEDro, and IEEE Xplore Digital Library.

The search strategy focused on older individuals, exoskeletons, functional ability, IC, and performance indicators, using relevant terms related to age groups and interventions. Specific outcome-related terms were not included due to the expected limited number of articles meeting inclusion criteria. The full search string is shown in Appendix A.

English-language studies were included if they involved the use of LLEs in human participants aged ≥50 years, with a mean age of ≥65 years. No publication date restrictions were imposed.

Studies that met one or more of the following criteria were excluded from the review: not related to wearable LLEs; performed in a different setting than hospitals, universities, rehabilitation centers, participants’ homes, and care facilities for older adults; evaluating different outcomes than participants’ intrinsic capacities, functional independence, quality of life, and functional abilities (i.e., the ability to meet their basic needs, to learn, grow, and make decisions, to be mobile, to build and maintain relationships, and to contribute to society); case report studies, research and project reports, annual or activity reports, theses, conference proceedings, pre-prints, newsletters, technical reports, recommendations and technical standards, patents, technical notes, presentations, field notes, laboratory research books, academic courseware, lecture notes, and evaluations.

### 2.3. Data Extraction and Analysis

First, references obtained from the systematic search were entered and deduplicated into EndNote X9 (Clarivate Analytics, Philadelphia, PA, USA). Second, titles and abstracts were screened for alignment with inclusion and exclusion criteria, employing the Rayyan QCRI web application. Selected studies’ full texts were subsequently reviewed for final inclusion. This selection process was conducted independently by two researchers, with disagreements resolved through discussion and, if necessary, consultation with a third reviewer.

Data extraction followed, utilizing a standardized collection form. The extracted information encompassed the first author’s name, publication year, study design, sample size, dropout count, patients’ clinical history, participants’ age and gender, exoskeleton specifications, intervention details, measurement systems employed, and outcomes. In cases of incomplete or unclear data, the corresponding author was contacted via e-mail for clarification.

### 2.4. Data Classification

**Figure 2:**
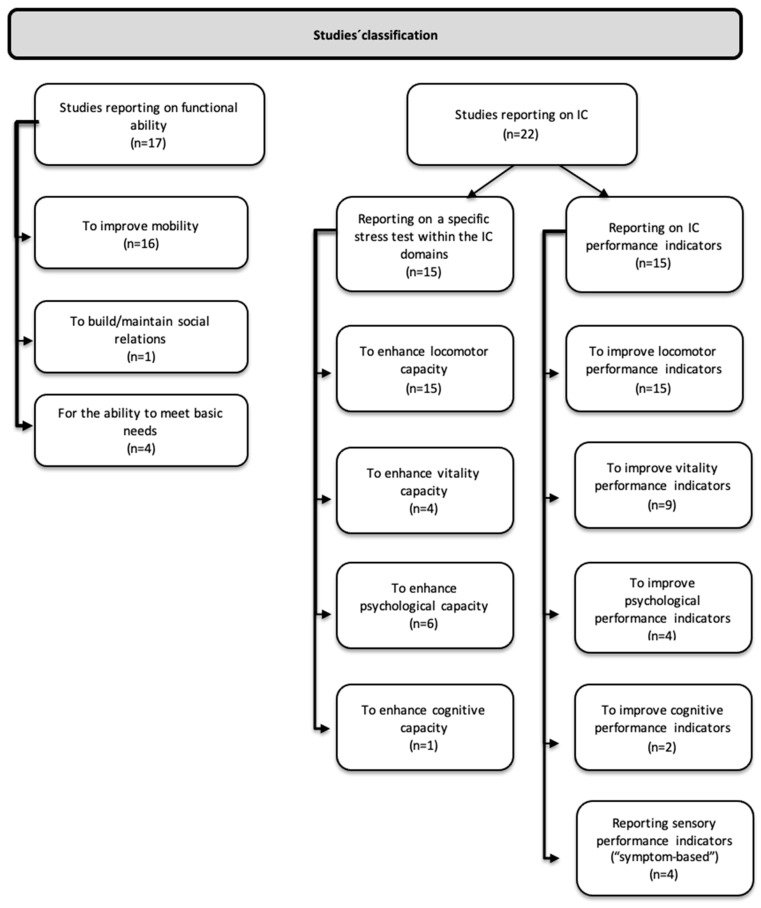
Studies’ classification.

The extracted results were classified following the WHO vision on healthy aging. Hence, two main categories were used: IC and functional ability. If studies reported on the effects of LLE use on functional ability (i.e., mobility, ability to learn, grow and make decisions, ability to build and maintain social relationships, ability to contribute, and ability to meet basic needs), they were allocated to the *functional ability* category. If studies reported effects of LLE use in light of measurements of IC (i.e., results of stress tests), they were allocated to the *intrinsic capacity* category. However, if studies reported on *performance* rather than on a specific stress test within the IC domains, they were allocated to a subcategory, i.e., the intrinsic capacity *performance indicator* category. This is shown in Figure 2.

### 2.5. Quality Assessment

The studies’ methodological quality was assessed using the Downs and Black Scale [31] [Appendix B] and the studies were classified as excellent (26–27), good (20–25), fair (15–19), or poor (≤14).

## 3. Results

The database search retrieved a total of 3622 records. A total of 2917 articles were screened by title and abstract and 792 full-text articles were assessed for eligibility, of which 36 articles were included in the qualitative synthesis [Figure 1 and Supplementary Material (Tables S1–S3)].

### 3.1. Studies’ Methodological Quality

A total of 22.2% of the included studies had a poor quality [32,33,34,35,36,37,38,39], 55.5% had a fair quality [24,40,41,42,43,44,45,46,47,48,49,50,51,52,53,54,55,56,57,58] and 22.2% had a good quality [59,60,61,62,63,64,65,66] [Appendix B].

### 3.2. Demographic and Study Characteristics

The included studies consisted of retrospective, prospective interventional, or observational research conducted in single or multi-center settings [Supplementary Materials (Tables S1–S3)]. A total of 17 studies investigated functional abilities [33,34,36,42,46,47,49,51,53,54,56,57,59,60,61,63,65] [Table S1 in Supplementary Material], 23 investigated IC [32,33,34,35,36,37,42,45,47,49,50,52,53,55,56,57,58,59,60,61,62,63,65] [Table S2 in Supplementary Material], and 26 investigated performance indicators [24,33,35,36,37,38,40,41,42,43,44,46,50,51,53,54,56,57,58,59,60,61,62,63,64,65] [Table S3 in Supplementary Materials] [Figure 2].

These studies covered a range of conditions, with ten investigating LLE use in patients with stroke [24,45,46,51,54,56,59,63,64,65], four focusing on patients with Parkinson’s Disease (PD) [38,58,60,61], two examining patient cohorts with various other neurological conditions [50,52], three centered on patients with osteoarthritis [42,53,57], and one each for hip fracture [34], sarcopenia [49], and depression [55]. Additionally, there were 14 studies involving healthy older adults [32,33,35,36,37,39,40,41,43,44,47,48,62,66] [Supplementary Materials (Tables S1–S3)].

### 3.3. LLEs to Improve Functional Ability

Seventeen studies assessed functional ability after the use of LLEs [33,34,36,42,46,47,49,51,53,54,56,57,59,60,61,63,65] and their results are detailed in Table S1 in the Supplementary Materials.

#### 3.3.1. LLEs to Improve Mobility

Mobility measures were considered in 16 studies [33,34,36,42,46,47,49,51,53,54,56,57,60,61,62,65], of which two focused on patients with PD [60,61], three on patients with osteoarthritis [42,53,57], one on patients with sarcopenia [49], one on patients with hip fracture [34], five on patients with stroke [46,51,54,56,65], and four on healthy older adults [33,36,47,62] [Table S1 in Supplementary Materials].

In patients with PD, Gryfe et al. (2022) [60] investigated the impact of an 8-week Keeogo Rehab^TM^ [B-Temia, Saint-Augustin-de-Desmaures, Quebec, Canada] exoskeleton intervention, demonstrating improvements in preferred gait speed and on the Freezing of Gait Questionnaire (FoG-Q) and Unified Parkinson’s Disease Rating Scale (UPDRS)—motor functioning sub-scale, and Parkinson’s Disease Questionnaire-39 (PDQ-39)—mobility sub-scale (*p* = 0.017) post-intervention, compared to the other groups. Kawashima et al. (2022) [61] studied the effects of a 3-month gait training intervention using the Stride Management Assist^®^ (SMA) [Honda, Tokyo, Japan] exoskeleton, but no significant changes were observed in the FoG-Q and 10 Meter Walk Test (10MWT) within and between groups.

In patients with osteoarthritis, Koseki et al. (2021) [42] demonstrated significant improvement in Western Ontario and McMaster University Osteoarthritis index (WOMAC) function scores at 8 weeks post total knee arthroplasty using the Honda Walking Assist^®^ (HWA) (ES = 0.21 at T0, ES = 0.18 at T1, ES = 0.54 at T2 and ES = 2.33 at T3), in comparison with the control group (*p* < 0.001) [Table S1]. Setoguchi et al. (2022) [53] and Yoshikawa et al. (2018) [57] explored Hybrid Assistive Limb^®^ (HAL) gait training post hip arthroplasty and post total knee arthroplasty (TKA), respectively. Setoguchi et al. (2022) [53] reported statistically significant within-group temporal changes in the Harris hip score function subscore for patients who received the LLE intervention and in the control group (*p* < 0.05), in the Harris hip score motion subscore for the control group (*p* < 0.05), in the 36-Item Short Form Survey (SF-36) physical functioning subscore for both groups (*p* < 0.05), and in the SF-36 role limitations subscore after LLE intervention (*p* < 0.05). Yoshikawa et al. (2018) [57] showed varying between-group improvements in the WOMAC function score at different time points, but did not consistently demonstrate statistically significant differences between groups at the different studied timepoints.

The study on sarcopenia by Norris et al. (2007) [49] with powered ankle–foot orthoses (PAFOs) revealed no statistically significant improvement in preferred walking speed. Comparisons between walking with standard shoes, inactive PAFOs, and active PAFOs did not yield statistically significant differences (*p* = 0.098, *p* = 0.536, and *p* = 0.474, respectively). However, there was a non-significant trend observed between walking with standard shoes and inactive PAFOs, suggesting a potential impact that did not reach statistical significance in this small cohort of older adults.

In patients with hip fracture, Fujikawa et al. (2022) [34] demonstrated substantial improvement in functional mobility with HAL^®^ rehabilitation, when combined with conventional rehabilitation, as indicated by the significant reduction in Five Times Sit-to-Stand test (FTSS) scores among patients with hip fracture (*p* < 0.01; ES = 1.81 (95% CI = 0.93—2.66)).

Five studies on patients with stroke explored various LLE interventions, showcasing improvements in functional outcomes but with mixed results in inter-group differences [46,51,54,56,65]. Taki (2020) [54] found an increase in Functional Independence Measure (FIM) motor subscores (*p* = 0.013) and in ambulation at a hospital ward on discharge (*p* = 0.011) after gait training with HAL^®^ for 3 h/day for 7 days/week. Longatelli (2021) [46] showed significant improvement in the Capacity Score of patients with stroke after a 4-week intervention consisting of 12 assisted rehabilitation sessions and 8 conventional therapy sessions, as well as in the control group (*p* < 0.01). Moreover, Watanabe (2017) [56] found statistically significant improvements in the FAC after 12 sessions of HAL over 4 weeks (*p* = 0.026), while the differences in the LE Fugl-Meyer Assessment were not statistically significant (*p* = 0.131).

Park et al. (2021) [51] conducted a study in which participants performed interlimb coordinated humanoid robotic sessions with a VR/AR game, along with conventional physical therapy 7 days/week, for 2 weeks. They found a within-group improvement on the Fugl-Meyer Assessment Lower Extremity (FMA-LE) synergy scale flexor synergy test (*p* = 0.000) and in FMA-LE synergy scale total synergy (*p* = 0.007) over time. Yeung (2021) [65] also reported differences between groups in the Functional Ambulation Category (FAC) over time after 30 min/weekday interventions with the Power-Assisted Ankle Robot and Swing-Controlled Ankle Robot, together with a conventional training routine (2 h/weekday) (increase of 1.4 [1.0, 1.9] (*p* < 0.001) in G1, increase of 1.4 [0.9, 2.0] (*p* < 0.001) in G2, and increase of 0.9 [0.4, 1.3] (*p* < 0.01) in G3).

Mobility in healthy older adults was studied in four studies [33,36,47,62]. First, Jayaraman et al. (2022) [36] conducted a study in which participants performed twelve gait training sessions over 4–6 weeks; these authors found statistically significant improvements in participants’ scores on the Functional Gait Assessment (FGA) (*p* < 0.001), number of sedentary bouts (>3 min) per day (*p* = 0.004), time spent in the sedentary bouts (*p* = 0.003), and 5xSTS (*p* < 0.001) post-intervention. Fang (2022) [33] evaluated two protocols involving assistance and resistance modes of an ankle exoskeleton and reported an improvement in participants’ self-selected walking speed (1.07 vs 1.12) from T0 to T1 and in fast walking speed (1.38 vs 1.59) over time. Martini (2019) [47] administered a four-week robot-assisted gait training regimen in an active pelvis orthosis (APO) group, measuring differences in daily steps at baseline, for which they found no significant changes [Table S1]. Finally, Lee (2022) [62] investigated the effects of a four-week exoskeleton exercise program. They found significant improvements in participants’ scores on the Short Physical Performance Battery (SPPB) over time (*p* < 0.01) [Table S1 in the Supplementary Material].

#### 3.3.2. LLEs to Improve Older Persons’ Ability to Build/Maintain Social Relations

One study assessed older persons’ ability to build and/or maintain social relations in patients with osteoarthritis after a 6-week HAL^®^ gait training intervention, which showed no significant impact on social functioning [53] [Table S1 in the Supplementary Materials].

#### 3.3.3. LLEs to Improve the Ability to Meet Basic Needs

For patients with stroke, three studies investigated the effects of LLE training on their ability to meet basic needs [54,59,63]. First, Rojek (2020) [63] investigated the effects of a four-week Ekso GT^®^ intervention, associated with occupational therapy and individually tailored physical therapy, and reported statistically significant improvements over time in the Barthel Index (*p* = 0.01) and Rivermead Mobility Index [Table S1]. Calabrò (2018) [59] investigated the effects of an 8-week Ekso™ training intervention, associated with transcranial magnetic stimulation (TMS), and also reported significant improvements in the Rivermead Mobility Index after LLE training (ES = 0.6, *p* = 0.03). Finally, Taki (2020) [54] investigated the effects of knee–ankle–foot orthoses (KAFO), ankle–foot orthoses (AFO), and HAL^®^ 3 h per day, 7 days per week, and reported statistically significant improvements over time in FIM total sores after gait training with HAL^®^ (*p* = 0.024).

In the case of patients with PD [60], an intervention using the Keeogo Rehab^TM^ powered knee assistance exoskeleton for eight weeks did not yield significant differences in ADL subscores [Table S1 in the Supplementary Materials].

### 3.4. LLEs to Enhance IC

The impact of LLEs to enhance IC has been investigated in 15 studies [32,33,34,36,42,50,56,57,58,59,60,61,62,63,65] and their results are detailed in Table S2 in the Supplementary Materials.

#### 3.4.1. LLEs to Enhance Locomotor Capacity

First, locomotor capacity was taken into account in fifteen studies [32,33,34,36,42,50,56,57,58,59,60,61,62,63,65], of which three focused on patients with PD [58,60,61], one on other neurological disorders [50], two in patients with osteoarthritis [42,57], four on patients with stroke [56,59,63,65], one on patients with hip fracture [34], and four on healthy older adults [32,33,36,62] [Table S2 in Supplementary Materials].

In patients with PD, Yun et al. (2019) [58] investigated the effects of a 4-week intervention using the Walkbot^®^, and reported significant improvements in the Berg Balance Scale (BBS) scores, which were observed immediately after treatment (*p* = 0.004) and at the one-month follow-up (*p* = 0.024). Gryfe et al. (2022) [60] conducted an 8-week exoskeleton exercise intervention, and showcased notable increases in the 6 Minute Walk Test (6MWT) for the exoskeleton group, compared to the others (*p* < 0.001). The study by Kawashima et al. (2022) [61] investigated the effects of 10 gait training sessions with the SMA^®^ exoskeleton for 3 months, and revealed positive outcomes, emphasizing statistically significant improvements in the 3 Minute Walk Test (3MWT) post-intervention (*p* = 0.023). However, they did not find statistically significant differences for the BBS and Functional Reach Test (FRT) in any of the groups [Table S2].

Panizzolo et al. (2022) [50] extended the exploration to patients with other neurological disorders beyond PD, utilizing the Exoband^®^[Moveowalks, Padua, Italy] in a ten-session walking program over five weeks. Participants wore the Exoband while walking for 10 min back and forth along a 60 m corridor. They were instructed to attempt to walk as far as possible (i.e., cover the longest possible walking distance) and were able to stop and rest during the walking session as needed. They reported a significant increase in the longest walking distance while wearing the Exoband^®^ (*p* < 0.05) and a statistically significant correlation between sessions spent walking with the Exoband^®^ and meters covered (r = 0.9126; *p* < 0.01).

For patients with osteoarthritis, Koseki et al. (2021) [42] administered 17–20 gait training sessions using the HWA^®^, from week 1 to 5 post-TKA and found statistically significant differences in the maximal passive and active extension of the knee between groups only at baseline (*p* = 0.027, ES = 1.02 and *p* = 0.031, ES = 0.99, respectively), but not at the other timepoints. At one week post-TKA, there was a significant improvement in the maximum walking speed (ES = 1.04), and at 1 and 2 weeks post-TKA, there was a significant improvement in step length at maximum walking speed (ES = 1.02 and ES = 0.87, respectively). Moreover, Yoshikawa et al. (2018) [57] administered 10–12 sessions of 15 min each with HAL^®^, over 4 weeks, along with conventional physical therapy. They found that passive knee extension ROM differences among groups were statistically significant at 2 weeks following TKA (*p* = 0.034) and 4 weeks post-TKA (*p* = 0.006), and that there was a significant between-group difference in maximal walking speed 4 and 5 weeks post-TKA (*p* = 0.006 and *p* = 0.027, respectively), and in step length at maximum walking speed in weeks 2, 4, and 5 post-TKA (*p* = 0.016, *p* = 0.001 and *p* = 0.003, respectively). However, the differences in active knee extension ROM among groups were only statistically significant at weeks 2 and 3 post-TKA (*p* = 0.005 and *p* = 0.048).

The locomotor capacity of patients with stroke was explored in studies by Calabrò et al. (2018) [59], Rojek et al. (2020) [65], Watanabe et al. (2017) [59,63], and Yeung et al. (2021) [65], which used the Ekso^®^ [65], HAL^®^ [56], and various robotic-assisted trainings [65]. Calabrò et al. (2018) [59] reported significant improvements in exoskeleton-assisted gait training (EGT)-induced functional outcomes, measured via the Timed Up and Go (TUG) test at 8 weeks post-gait training (ES = 0.5, *p* < 0.02), while Rojek et al. (2020) [63] reported significant changes in both balance and functional status after EGT, together with a slight and insignificant trend towards reducing the total load distribution on the feet, particularly on the uninvolved limb. Similarly, Yeung et al. (2021) [65], found significant improvements in BBS scores post-intervention in the whole group (increase of 18.8 [13.1, 24.4] (*p* < 0.001) in G1, increase of 12.6 [6.2, 18.9] (*p* < 0.01) in G2, and increase of 14.4 [9.4, 19.3] (*p* < 0.001) in G3).

Watanabe et al. (2017) [56], however, did not find statistically significant differences among groups in maximal walking speed (*p* = 0.975), 6MWT (*p* = 0.810), and TUG (*p* = 0.413) after 12 HAL sessions.

Fujikawa et al. (2022) [34] conducted the only study investigating locomotor capacity in patients with hip fracture, implementing conventional rehabilitation alongside HAL^®^ rehabilitation and reporting a reduction in TUG over time in all participants.

Four studies investigated locomotor capacity in healthy older adults [32,33,36,62]. First, Jayaraman et al. (2022) [36] conducted a study in which participants used the Gait Enhancing and Motivating System (GEMS-H) during 12 gait training sessions (30 min each) over a period of 4–6 weeks and saw an improvements in the 10MWT (*p* = 0.001), 6MWT (*p* < 0.001) and BBS scores (*p* < 0.001). Aprigliano et al. (2019) [32] administered one session and 14 experimental trials with the APO. They reported that the assistive approach effectively enhanced balance recovery in the sagittal plane for both perturbation paradigms. However, it did not demonstrate effectiveness in maintaining stability in the frontal plane. Fang et al. (2022) [33] conducted a study in which participants used a dual-mode ankle exoskeleton for ankle assistance and resistance; these authors found that the resistance protocol produced a 35% increase in 6MWT distance (m), an increase of 18% (right side) and 43% (left side) in plantar flexor strength, and an increase in fast walking speed (m/s) over time. Lee et al. (2022) [62] administered a 4-week intervention using EX1^®^ and reported significant improvements in BBS scores (*p* < 0.01), TUG (*p* < 0.01), and FRT (*p* < 0.01) for all the groups, with associated changes in muscle strength.

#### 3.4.2. LLEs to Enhance Vitality Capacity

In addition to examining locomotor capacity, several studies delved into vitality capacity across diverse patient groups [Table S2 in Supplementary Material].

Kawashima et al. (2022) [61] examined vitality in patients with PD, using the SMA^®^ exoskeleton for 3 months, revealing a significant reduction in energy expenditure as measured by the Physiological Cost Index (PCI) during the 3MWT after the SMA intervention (*p* = 0.046).

In patients with osteoarthritis, Koseki et al. (2021) [42] conducted 17–20 gait training sessions with the HWA^®^, reporting no significant changes in knee extension and flexion torque 1 to 5 weeks after TKA. Conversely, Yoshikawa et al. (2018) [57], using HAL^®^, observed significant changes in knee extension torque 5 weeks post-intervention (*p* = 0.014).

Lefeber et al. (2018) [45] focused on the immediate effect of walking with a Lokomat^®^ across three conditions in patients with stroke, and reported significant differences in the net oxygen consumption (*p* = 0.037), net respiratory exchange ratio (*p* = 0.047), and the net oxygen cost (*p* = 0.037) after 6 min of walking based on the implemented level of assistance.

#### 3.4.3. LLEs to Enhance Psychological Capacity

Psychological capacity was assessed in studies involving patients with PD [58,60], osteoarthritis [53], depression [55], sarcopenia [49], and healthy older adults [62] [Table S2 in Supplementary Materials].

Two studies in patients with PD investigated the effects of LLEs on psychological capacity [58]. Yun et al. (2019) [58] administered a 4-week intervention using the Walkbot^®^ and did not find significant changes in the Korean version of the Falls Efficacy Scale (KFES). Gryfe et al. (2022) [60] administered an 8-week intervention with the Keeogo Rehab^TM^ and did not find significant decreases in the Activities-Specific Balance Confidence (ABC) test for any group at any timepoint.

Verrusio et al. (2018) [55] investigated psychological capacity in patients with depression using the Human Body Posturizer^®^ (HBP) [Posturizer, Italy] (3 sessions/week for 6 months) and observed a reduction in the Geriatric Depression Scale (*p* < 0.01).

Norris et al. (2006) [49] explored vitality capacity in patients with sarcopenia, utilizing the EXO3^®^ exoskeleton. Significant improvements in the SF-36 vitality sub-scale were reported.

Lee (2022) [62] investigated psychological capacity in healthy older adults, using the EX1^®^ for 4 weeks, reporting improvements in the Geriatric Depression Scale Short Form (GDS-SF) (*p* < 0.05).

#### 3.4.4. LLEs to Enhance Cognitive Capacity

In patients with PD, Yun et al. (2019) [58] assessed cognitive capacity with Walkbot^®^ interventions over 4 weeks. They reported a tendency towards an increase in dual-task interference in gait velocity, although differences were not statistically significant, but not in dual-task physical aspects [Table S2].

### 3.5. LLEs to Improve Performance Indicators

The impact of lower limb exoskeletons (LLEs) on various performance indicators has been the subject of extensive research, with multiple studies shedding light on their effects in different populations [24,38,42,46,51,53,54,56,57,58,59,61,63,64,65] [Table S3 in Supplementary Materials].

#### 3.5.1. LLEs to Improve Locomotor Performance Indicators

Fifteen studies investigated locomotor performance indicators [24,38,42,46,51,53,54,56,57,58,59,61,63,64,65] [Table S3 in Supplementary Material].

In the domain of locomotor performance for patients with PD, significant findings emerged. Romanato et al. (2022) [38] observed notable improvements in muscle forces during various walking phases after 4 weeks of EksoGT^®^ training (*p* < 0.05). Yun et al. (2020) [58], using the Walkbot^®^ for 4 weeks of gait training, reported a significant increase in the 10MWT comfortable gait speed over time (*p* = 0.041). Kawashima et al. (2022) [61], in a 3-month randomized controlled trial (RCT) with the SMA^®^ exoskeleton, reported significant enhancements in walking speed, step length, and ranges of motion.

Studies focusing on interventions for patients with osteoarthritis also showed positive outcomes [42,53,57]. Setoguchi et al. (2022) [53] conducted a study in which participants used the HAL^®^; these authors reported significant improvements in hip extension and range of motion over time (*p* < 0.05). Koseki et al. (2021) [42] conducted a study in which participants performed 17–20 sessions using the HWA^®^ after TKA, and demonstrated notable changes in self-selected walking speed (*p* = 0.022) and step length (*p* = 0.032), and Yoshikawa et al. (2018) [57] also found significant differences in self-selected walking speed (*p* = 0.022-*p* = 0.030) and step length (*p* = 0.002-*p* = 0.011) after participants performed 10–12 HAL^®^ training sessions.

Nine studies investigated locomotor performance indicators in patients with stroke [24,46,51,54,56,59,63,64,65]. Calabrò et al. (2018) [59] conducted a study in which participants used Ekso^®^ for 8 weeks, and reported significant improvements in walking speed during the 10MWT (ES = 0.9, *p* < 0.001), hip and knee muscle activation (ES = 0.8, *p* = 0.001), gait quality index (ES = 0.9, *p* < 0.001), step cadence (ES = 0.9, *p* < 0.001), and stance/swing ratio in the affected limb (ES = 0.8, *p* = 0.008). Also, they reported EGT-induced reductions in gait cycle duration in the affected and unaffected limb, and stance/swing ratio in the unaffected limb (ES = 0.9, *p* < 0.001).

Longatelli et al. (2021) [46] found selective improvements in patients’ muscular activation strategies, especially in the semitendinosus muscle, and Rojek et al. (2020) [63] observed that patients increased their walking time and steps during LLE gait therapy; both studies administered a 4-week Ekso^®^ intervention. Taki et al. (2020) [53] conducted a study in which participants used HAL^®^ 3 h/day for 7 days/week and found no significant differences in Br-stage between groups. Watanabe et al. (2017) [56] investigated the effects of 12 sessions with HAL^®^ and they reported no statistically significant differences after training. Also, Park et al. (2021) [50] reported significant improvements in the hip and knee angles and active forces of patients after a 2-week intervention using the Walkbot^®^.

Firouzi et al. (2022) [24] conducted a study assessing the immediate effects of the HWA^®^ during three walking conditions: normal walking at a self-selected comfortable speed (I1), unassisted walking with HWA^®^ (I2), and optimal assisted walking with HWA^®^ (I3). Each condition involved walking three times on a 5 m walkway, totaling 40–60 min. Comparisons between conditions revealed that walking speed increased in most patients in I1 vs I3, showed mixed results in I1 vs I2, and uniformly increased in I2 vs I3. Stride lengths and velocities generally increased across interventions for both paretic and non-paretic limbs. The paretic swing phase increased in I1 vs I3, while the non-paretic swing phase increased in I2 vs I3. Paretic and non-paretic stance phases and double support phases exhibited mixed results across interventions. Moreover, percentage changes in various parameters demonstrated individual-specific improvements or declines.

Son et al. (2021) [64], administering 10 Healbot T sessions, reported significant increases in self-selected speed for both pelvis-off and pelvis-on groups, and notable improvements in muscle activity, stride length, cadence, and walking speed. Finally, Yeung et al. (2021) [65] conducted a study in which participants utilized the Power-Assisted Ankle Robot and Swing-Controlled Ankle Robot for 30 min/weekday, together with a conventional training routine (2 h/weekday), and reported significant increases in self-selected walking speed (10 MWT) over time for all groups (increase of 0.32 [0.18, 0.46] (*p* < 0.001) at G1, increase of 0.17 [0.09, 0.25] (*p* < 0.01) at G2, and increase of 0.17 [0.06, 0.29] (*p* < 0.01) at G3).

Eight studies investigated locomotor performance indicators in healthy older adults [33,35,36,37,41,44,62].

Jayaraman et al. (2022) [36] conducted a study in which participants used GEMS-H^®^ in twelve gait training sessions (30 min each) over a period of 4–6 weeks; these authors reported an improvement in 10MWT self-selected gait speed (*p* = 0.001).

Lee et al. (2017) [43] conducted a study in which participants performed one session with the GEMS^®^ at their own comfortable speed; these authors found statistically significant increases in gait speed, cadence, stride length, step width, and single support time with robot assistance (*p* < 0.01), and they also found a reduction in muscle activation due to hip assistance. Lee et al. (2017) [44], using the same protocol, reported an increase in gait speed, stride length, cadence, and single support time with robot assistance, together with reduced muscle activity in the rectus femoris and medial gastrocnemius throughout the terminal stance phase, and the medial gastrocnemius throughout pre-swing phase. This was associated with an increase in the maximum force and peak pressure.

Jin et al. (2017) [37] conducted a study in which participants used a soft wearable robotic suit built in-house for 2 days (four 6-min treadmill trials, at fixed preferred walking speed)); these authors reported an increase in maximum hip angle (*p* < 0.05), maximum vertical displacement of center of mass (COM) (*p* < 0.01), maximum vertical position of knee (*p* < 0.001), maximum vertical position of ankle (*p* = NS), maximum vertical position of toe (*p* < 0.001), stride duration (*p* < 0.001), and walk ratio (*p* < 0.001) with the robotic suit powered on. Additionally, Jin et al. (2019) [41] conducted a study in which participants performed a 6-week training intervention using a soft robotic suit; these authors reported an average increase in the maximum hip angle, maximum knee angle, maximum ankle angle, and walk ratio (*p* = 0.0052) with the device powered on. When the device was powered off, there was a reduction of the maximum hip flexion (*p* = 0.0411), maximum knee flexion (*p* = 0.0350), and maximum ankle dorsiflexion (*p* = 0.0085).

Fang et al. (2022) [33] conducted a study in which participants used a dual-mode ankle exoskeleton during two visits; using soleus imaging electromyography (iEMG), these authors observed changes in the minimum soleus variance ratio and stance phase with the ankle resistance protocol. Galle et al. (2022) [35] conducted a study in which participants performed four trials using bilateral ankle–foot exoskeletons at a fixed speed; these authors reported statistically significant changes in step length and walking conditions with the exoskeleton. Finally, Lee et al. (2022) [62] conducted a study in which participants used the EX1^®^ for 4 weeks; these authors reported significant changes in 10MWT self-selected velocity over time.

#### 3.5.2. LLEs to Improve Vitality Performance Indicators

Panizzolo et al. (2022) [50] investigated vitality performance indicators in other neurological conditions beyond PD. They conducted a study in which participants used the Exoband^®^ in 10 walking sessions (10-min each) for 5 consecutive weeks, and reported a rate of perceived exertion (RPE) difference over the 10 sessions (<0.05), and a negative correlation between the sessions spent walking with Exoband^®^ and RPE (*p* < 0.01).

Setoguchi et al. (2022) [53] investigated vitality performance indicators in a study in which particpants performed three HAL sessions/week for 6 weeks, but these authors only reported statistically significant within-group changes for the SF-36 vitality category in the control group (*p* < 0.05).

Norris et al. (2006) [49] conducted a study in which patients with sarcopenia used a PAFO for ankle plantarflexion assistance; these authors observed a lower metabolic cost of transport and metabolic energy per stride at preferred walking speeds with the PAFOs active.

In healthy older adults, vitality performance indicators were assessed in six studies [33,35,37,47,62].

First, Jin et al. (2017) [37] conducted a study in which participants performed four 6-min treadmill walking trials at a preferred walking speed using a soft wearable robotic suit built in-house; these authors performed two sets of measurements in 2 days, and reported a lower energy expenditure and energy efficiency (*p* < 0.05) with the robotic suit powered on. Martini et al. (2019) [47] conducted a study in which participants used the APO at a fixed walking speed for 4 weeks; these authors observed significant reductions in the metabolic cost of transport (*p* < 0.01), and increases in oxygen uptake rate (*p* = 0.0024) and metabolic power (*p* = 0.011) post-intervention.

Fang et al. (2022) [33] conducted a study in which participants used a dual-mode ankle exoskeleton at a fixed walking speed and observed that four out of five participants experienced a reduction (up to 19%) in metabolic power during assisted walking compared to their baseline, especially in participants with higher baseline metabolic power, while the metabolic power decreased 9% over time using the resistance training protocol.

Galle et al. (2022) [35] conducted a study in which participants used bilateral ankle–foot exoskeletons in four walking trials at a fixed walking speed; these authors reported statistically significant changes in the net metabolic power (I1 > I4 (*p* = 0.05, ES = 0.91)) and an increased perceived fatigue in the legs given by the visual analogue scale (VAS) over time (*p* = 0.05). Lee et al. (2022) [62] administered a 4-week intervention using the EX1^®^ and reported reductions in the participants’ net cardiopulmonary metabolic cost (*p* < 0.05) post-intervention. Finally, Lee et al. (2017) [43] investigated the immediate effect of using the GEMS^®^ at the participants’ own comfortable speed across three conditions. They reported an oxygen consumption per unit mass that was about 7% lower while using full robot assistance vs no robot assistance at self-selected speeds (*p* < 0.05); the EEm (kcal/min) at the participants’ own comfortable speed was 6.6% lower while using full robot assistance vs no robot assistance at self-selected speeds (*p* < 0.05) [Table S3 in Supplementary Materials].

#### 3.5.3. LLEs to Improve Psychological Performance Indicators

The effects of LLEs on psychological performance indicators were also explored in specific populations. Gryfe et al. (2022) [60] investigated them in patients with PD, using Keeogo Rehab^TM^ for 8 weeks, and revealed a significant decrease in the PDQ-39 emotional well-being sub-scale, but did not find statistically significant changes over time in the Hospital Anxiety and Depression Scale (HADS) anxiety score, the HADS depression score, the UPDRS mentation sub-scale, and the PDQ-39 stigma sub-scale. Carral et al. (2022) [40], however, delved into the psychological aspects in healthy older adults using AUTONOMYO^®^ [Autonomyo, Switzerland], highlighting participants’ perceptions of enhanced autonomy while also perceiving that their usage would alleviate the sense of burden they might impose on their support network. However, there was a degree of ambivalence among participants, influenced by their personal experiences of the aging process and their perceptions of human–machine interactions [Table S3 in Supplementary Materials].

Roggeman et al. (2022) [52] conducted a study in which older patients used the HWA^®^ while performing 30 min of walking; these authors reported the following median scores using the Intrinsic Motivation Inventory (IMI): 43 for interest/enjoyment, 36 for perceived competence, 19 for effort/importance, 6 for pressure/tension, 45 for value/usefulness, and 32 for relatedness.

Setoguchi et al. (2022) [53] assessed psychological indicators in patients with osteoarthritis using the HAL^®^ during three sessions per week for 6 weeks in total, reporting significant improvements in the SF-36 role emotional scores (*p* < 0.05), but not statistically significant changes for the SF-36 mental health scores.

#### 3.5.4. LLEs to Improve Cognitive Performance Indicators

Gryfe et al. (2022) [60] conducted a study in which patients with PD used Keeogo Rehab^TM^ for 8 weeks; these authors reported significant increases in the SCales for Outcomes in PArkinson’s disease-COGnition (SCOPA-COG) (*p* = 0.003) and memory and learning (*p* = 0.001) over time. Moreover, Taki (2020) [54] conducted a study in which participants used the HAL^®^ 3 h/day for 7 days/week; these authors found statistically significant differences over time in the FIM cognitive subscore (*p* = 0.008) [Table S3 in Supplementary Materials].

#### 3.5.5. Sensory Performance Indicators (“Symptom-Based”)

Gryfe et al. (2022) [60] investigated sensory performance indicators (“symptom-based”) in patients with PD. These researchers administered the Keeogo Rehab^TM^ for 8 weeks of aerobic strength and functional mobility exercises and reported no statistically significant changes in the PDQ-39 communication sub-scale and the PDQ-39 bodily discomfort sub-scale over time.

Three studies investigated sensory performance indicators (“symptom-based”) in patients with osteoarthritis [42,53,57]. First, Koseki et al. (2021) [42] conducted a study in which participants performed 17–20 sessions using the HWA^®^, but these authors did not find any statistically significant changes in the WOMAC-p for any group at any timepoint.

Setoguchi et al. (2022) [53] conducted a study in which participants used the HAL^®^ for 6 weeks; these authors found within-group changes in the Harris hip score pain subscore and in the Harris hip score bodily pain subscore (*p* < 0.05). Finally, Yoshikawa et al. (2018) [57] also conducted a study in which participants used the HAL^®^ for 4 weeks; these authors found statistically significant changes in the WOMAC-p (*p* = 0.021) over time [Table S3 in Supplementary Materials].

## 4. Discussion

In this systematic review, we aimed to shed light on the potential of LLEs in the context of healthy aging. We therefore comprehensively assessed the impact of LLEs on the intrinsic capacities, functional abilities, and physical performance of older adults, in alignment with the WHO’s healthy aging vision. We reviewed 36 studies, encompassing healthy individuals and individuals with a spectrum of health conditions, including stroke, PD, osteoarthritis, hip fracture, sarcopenia, and depression.

Our findings collectively reveal consistently positive outcomes in various intrinsic capacities crucial to healthy aging, manifesting after as few as one to forty LLE sessions. Across all health conditions and in healthy individuals, LLE interventions showed notable improvements on stress tests, measuring maximum capability across the subdomains of locomotor capacity, vitality capacity, and psychological capacity. Specifically, maximum walking speed, step length at maximum walking speed, and maximum walking distance were increased following LLE training. Apart from these locomotor improvements, significant enhancements in energy expenditure, perceived vitality, and depressive symptoms were also reported.

Furthermore, significant improvements were observed in a wide range of performance indicators spanning the same subdomains. Locomotor enhancements encompassed improved muscle strength, muscle activation, joint angles, stance and swing phases, single and double support phases, stride and step lengths, number of steps, cadence, and self-paced gait speed. Regarding the other subdomains, significant improvements in energy expenditure, energy efficiency, muscle exertion, pain perception, and perceived vitality were also reported. Interestingly, while psychological performance indicators revealed modest or no changes in most populations, improvements in depressive symptoms were reported in those with depression and in healthy older adults.

The positive effects of LLEs extend beyond IC, with notable improvements in functional abilities, encompassing mobility and the ability to meet basic needs. For instance, individuals with PD showed improvements in their preferred gait speed, overall motor functioning, mobility, and severity of freezing of gait, suggesting a potential impact on slowing PD progression and enhancing functional mobility. Similarly, patients post-TKA, hip fracture, and stroke also showed improvements in various functional mobility measures. Finally, although positive effects of LLEs on functional outcomes are expected in individuals with impairments due to physical limitations, a key takeaway from this review is that healthy older adults also experienced positive effects of LLE training on their intrinsic capacities and functional abilities, including lower extremity function, physical performance, walking speed, overall sedentary behavior, and functional mobility. These findings align with previous work by Federici and colleagues (2015) [67], reinforcing the positive effects of LLEs in patients with neurological conditions, and extending their potential benefits for healthy older adults. Although the majority of existing exoskeletons are designed to assist in the rehabilitation of neurological populations or to augment human performance in industrial environments, their implications exceed physical functioning. Hence, LLEs should be considered as pivotal instruments in helping older adults maintain functional abilities, promoting independent living, and engaging in active lives as they age. Therefore, future research should also focus on evaluating LLEs in realistic home environments by implementing relevant activities of daily living to gain a deeper understanding of their potential benefits beyond a standard lab setting.

When considering the potential of LLEs for assessing, monitoring, and promoting older individuals’ health, it is imperative to contextualize these findings within the broader framework of IC, which has been introduced and discussed as a marker of healthy aging by the WHO [68,69]. Unlike the traditional disease-centered paradigm, which often fails to adequately address the complex and heterogeneous needs of older individuals, IC offers a comprehensive perspective, potentially aiding health monitoring through various technologies [70], where LLEs could play a significant role. While IC gains traction as a standard for measuring and monitoring older adults’ health [70,71], its integration in research and clinical practice remains limited. For instance, although most studies included in this review postdated the WHO’s introduction of the concept of IC as part of its World Report on Aging and Health [1], there remains considerable heterogeneity in the outcome measures employed. This variability can be attributed to the lack of standardized criteria for assessing IC subdimensions, as well as for quantifying IC as a global measure [69]. Although there is no consensus on assessing IC dimensions or establishing a global IC score, the WHO has already provided outcome measure recommendations for each dimension, as reviewed by Lopez-Ortiz et al. (2022) [69].

In light of these considerations, future research endeavors should adhere to the WHO’s recommendations for assessing IC subdimensions and prioritize interventions and innovative concepts to optimize IC in older adults. Modern technologies, like LLEs, have the potential to target specific IC subdimensions, and enable the development of interventions that could effectively maintain functional abilities or reverse functional loss throughout life [72].

In the context of neurological rehabilitation, LLE training benefits are founded on the assumption that task repetition would enhance motor learning and increase functional recovery [73]. Although improvements in gait parameters have been documented during a single LLE session post-stroke [24], assumedly, more than one session would be necessary to observe the substantial, lasting benefits of task repetition. Outside the context of neurological rehabilitation, however, this principle may be less applicable. For instance, analogous to how some exoskeletons can instantaneously, but artificially augment the user’s capabilities to levels exceeding that of normal human performance (e.g., allowing the user to carry heavy loads with minimal effort [74]), LLEs could be employed in healthy older adults to augment muscle strength and endurance, increase movement speed, and improve gait patterns (e.g., by increasing stride lengths and reducing asymmetries) [2]. Such improvements are not only confined to periods when users wear the exoskeletons but may extend to high-intensity activities, such as hiking or keeping pace with grandchildren [33]. Consequently, LLEs may offer an effective solution to address aging-related challenges by increasing habitual levels of physical activity and securing independent daily life in the long-term [2,75].

Nonetheless, it remains challenging to pinpoint the threshold of fatigue, effort, pain, or fall risk that would prompt individuals to opt for exoskeleton-assisted mobility in their daily lives [2]. A crucial aspect to consider in this regard is the adherence to user-centric design principles, encompassing factors such as the user-friendliness, safety, and comfort of LLEs [3]. While many prototypes and even some commercial LLEs currently fall short in these areas, the field and technologies are rapidly evolving, paving the way for more widespread use in the foreseeable future.

Apart from current limitations related to the design of LLEs, this review exposes several other gaps in the current understanding of LLE technologies within the context of healthy aging. For one, further research is urgently required to elucidate the relationship between the mechanisms of action of diverse LLEs and the targeted outcomes. Additionally, identifying the most effective LLE type, dosage, and intervention protocols for older adults of various health statuses is essential. Our review encompassed multiple studies utilizing a wide range of LLEs, different intervention durations, and a variety of outcome measures. For instance, various studies reported improvements in a wide range of parameters after interventions lasting two [51], four [37,46,47,56,57,58,62,63,64], five [42,50], six [36,41,53], and eight weeks [59,60]. Interestingly, Galle et al. (2022) [35], Lefeber et al. (2018) [45], Firouzi et al. (2022) [24], and Lee et al. (2017) [43] reported improvements even after a single session. Conversely, Kawashima et al. (2022) [61] did not observe improvements after a three-month intervention and Watanabe et al. (2017) [56] did not find a significant impact on maximal walking speed and functional mobility after 12 sessions.

Evidently, the included studies exhibited high heterogeneity in the health conditions studied, reported outcome measures, and intervention durations, ranging from one to 40 sessions. While this heterogeneity poses challenges in interpreting and synthesizing the reported findings, this review employed the WHO’s framework for the measurement of healthy aging as a guiding structure to descriptively categorize the included studies [28]. To this end, it is critical to distinguish between the *rehabilitation* of IC (e.g., muscle strength, balance, endurance) and the *augmentation* of functional abilities (e.g., walking, stairclimbing, sit-to-stand transfers), when considering LLE interventions.

Similar to conventional rehabilitation strategies, the LLE-focused rehabilitation of IC takes time. Extensive training programs are necessary to induce long-lasting, clinically relevant improvements in IC and functional abilities. This is supported by the observed trend indicating that longer intervention durations tend to yield more significant improvements across various performance indicators, including locomotor function, vitality, psychological well-being, cognitive capacity, and sensory symptoms. Studies with longer intervention periods, particularly those exceeding four weeks and involving three sessions per week, consistently report more substantial enhancements in functional mobility, gait parameters, psychological measures, and cognitive function.

On the other hand, immediate effects can be expected regarding the augmentation of functional abilities through LLEs. Analogous to how impaired vision can be instantaneously augmented—but not intrinsically enhanced—by corrective glasses, LLEs have the potential to augment older individuals’ functional abilities. This is further evidenced by the immediate benefits observed in specifically targeted parameters in single-session trials [24,43,45].

There is currently no global consensus on measurement methods for IC and functional ability, hindering widespread application. To enable comparison between exoskeleton types and outcome measures, standardized measurement protocols are required to allow better comparisons between groups. Future studies may reveal differential benefits based on factors like locomotion capacity, requiring tailored training approaches. In clinical practice, optimizing IC trajectory through screening, in-depth assessment, personalized care plans, and community engagement is crucial. LLE manufacturers should consider parameters related to older patients’ functional abilities and intrinsic capacities to design efficient exoskeletons. Additionally, involving older adults in the development process, through co-design methodologies, could enhance the effectiveness and acceptance of such technologies [76].

Unfortunately, our study encountered limitations in making outcome comparisons due to the substantial variability in study characteristics and participant profiles among the included studies. The limited number of eligible studies also reflected the scarcity of the literature on lower limb exoskeleton use in older adults, compounded by insufficient details regarding participant heterogeneity, comorbidities, and clinical management. Future investigations should aim to address these variables to mitigate potential biases. Moreover, we were not able to study differences in outcomes by training duration and frequency, mainly due to the high heterogeneity among studies. However, future studies should focus on further investigating the association between training effects and intervention frequency and duration in older adults.

In summary, the findings reviewed here underscore the potential of LLEs to enhance IC and support the implementation of LLEs to augment functional abilities in older adults, regardless of their health status. While numerous gait rehabilitation exoskeletons are available for clinical settings, the range of exoskeletons designed to address IC and functional ability in daily life for older adults is limited. Therefore, we believe that exoskeleton manufacturers and clinicians should work together towards the development of better exoskeletons, which could effectively support independent living, and enhance the overall well-being of older adults.

## 5. Conclusions

This review revealed consistent and remarkable improvements in various key parameters across all studied health conditions following LLE training. These improvements encompassed functional abilities, IC, and performance indicators, leading to improvements in QoL. Although longer intervention durations tend to yield more substantial improvements across various indicators and aid in the rehabilitation of IC (e.g., muscle strength, balance, endurance), even the instantaneous augmentation of functional abilities (e.g., walking, stairclimbing, sit-to-stand transfers) can be observed in a single session. These findings underscore the potential of LLEs in promoting healthy aging and enhancing the well-being of older adults.

## Data Availability

No new data were created or analyzed in this study. Data sharing is not applicable to this article.

## Supplementary Materials

The following supporting information can be downloaded at: https://www.mdpi.com/article/10.3390/s24072230/s1. Table S1: Summary of studies which used exoskeletons and assessed functional ability; Table S2: Summary of studies which used exoskeletons and assessed intrinsic capacity; Table S3: Summary of studies which used exoskeletons and assessed performance indicators.

## Appendix A. Systematic Search’s Full String

**PubMed**

**Concept 1: older adults**
“Aged”[Mesh] OR “oldest old”[tiab] OR quinquagenarian*[tiab] OR sexagenarian*[tiab] OR septuagenarian*[tiab] OR octogenarian*[tiab] OR nonagenarian*[tiab] OR frail*[tiab] OR “functionally impair*” [tiab] OR “older adult*” [tiab] OR senior*[tiab] OR retire*[tiab] OR “Geriatrics”[Mesh:NoExp] OR geriatric*[tiab] OR ((50[tiab] OR 55[tiab] OR 65[tiab] OR 70[tiab] OR 75[tiab] OR 80[tiab] OR 85[tiab] OR 90[tiab] OR 95[tiab] OR 100[tiab]) AND (age[tiab] OR aged[tiab] OR ages[tiab] OR old[tiab] OR year*[tiab])) OR Aged[tiab] OR “advanced age*”[tiab] OR “advancing years” [tiab] OR ageing[tiab] OR aging[tiab] OR elder*[tiab] OR gerontology[MeSH] OR gerontolog*[tiab] OR “old adult*” [tiab] OR “old age*” [tiab] OR “rest home*”[tiab] OR “very old”[tiab] OR “Nursing Homes”[Mesh] OR “nursing home*”[tiab] OR “Health Services for the Aged”[Mesh] OR “Homes for the Aged”[Mesh] OR “Housing for the Elderly”[Mesh] OR “senior center*” [tiab]

**Concept 2: exoskeletons**
“Exoskeleton Device”[Mesh] OR “exoskeleton*”[tiab] OR “Exoskeleton Device*”[tiab] OR “robotic exoskeleton*”[tiab]

**Embase**

**Concept 1: older adults**
‘aged’/exp OR ‘aged’:ti,ab,kw OR ‘elderl*’:ti,ab,kw OR ‘geriatric care’/exp OR ‘geriatric nursing’/exp OR ‘home for the aged’/exp OR ‘old age assistance’:ti,ab,kw OR ‘aged hospital patient’/exp OR ‘senior center’/exp OR ‘geriatric rehabilitation’/exp OR ‘nursing home’/exp OR ‘nursing home*’:ti,ab,kw OR ‘skilled nursing facilit*’:ti,ab,kw OR ‘nursing home patient’/exp OR ‘frail elderly’/exp OR ‘geriatric’/exp OR ‘geriatric*’:ti,ab,kw OR ((50 OR 55 OR 65 OR 70 OR 75 OR 80 OR 85 OR 90 OR 95 OR 100) NEAR/3 (age OR ages OR aged OR old OR year*)):ti,ab,kw OR ‘oldest old’:ti,ab,kw OR ‘quinquagenarian*’:ti,ab,kw OR ‘sexagenarian*’:ti,ab,kw OR ‘septuagenarian*’:ti,ab,kw OR ‘octogenarian*’:ti,ab,kw OR ‘nonagenarian*’:ti,ab,kw OR ‘frail*’:ti,ab,kw OR ‘functionally impair*’:ti,ab,kw OR ‘older adult*’:ti,ab,kw OR ‘senior*’:ti,ab,kw OR ‘retire*’:ti,ab,kw OR ‘advanced age*’:ti,ab,kw OR ‘advancing years’:ti,ab,kw OR ‘ageing’:ti,ab,kw OR ‘aging’:ti,ab,kw OR ‘gerontolog*’:ti,ab,kw OR ‘old adult*’:ti,ab,kw OR ‘old age*’:ti,ab,kw OR ‘rest home*’:ti,ab,kw OR ‘very old’:ti,ab,kw

**Concept 2: exoskeletons**
‘exoskeleton’/de OR ‘exoskeleton*’:ti,ab,kw OR ‘Exoskeleton Device*’:ti,ab,kw OR ‘robotic exoskeleton*’:ti,ab,kw

**WOS**

**Concept 1: older adults**
“aged” OR “elderl*” OR “oldest old” OR ‘’quinquagenarian*’’ OR ‘’sexagenarian*’’ OR ‘’septuagenarian*’’ OR ‘’octogenarian*’’ OR ‘’nonagenarian*’’ OR ‘’ frail*’’ OR “functionally impair*” OR “older adult*” OR ‘’senior*’’ OR ‘’retire*’’ OR ‘’geriatric*’’ OR “advanced age*”OR “advancing years” OR ‘’ageing’’ OR ‘’aging’’ OR ‘’gerontolog*’’ OR “old adult*” OR “old age*” OR “rest home*” OR “very old” OR ‘’nursing home*’’OR “old age assistance“ OR ‘’skilled nursing facilit*’’ OR “50” OR “55” OR “65” OR “70” OR “75” OR “80” OR “85” OR “90” OR “95” OR “100”

**Concept 2: exoskeletons**
“exoskeleton*” OR “Exoskeleton Device*” OR “robotic exoskeleton*”

**Cochrane**

**Concept 1: older adults**
#1: [mh “Aged”]
#2: [mh “Health Services for the Aged”]
#3: [mh “Senior Centers”]
#4: [mh “Geriatrics”]
#5: [mh “Gerontology”]
#6: [mh “Housing for the Elderly”]
#7: [mh “Nursing Homes”]
#8: (“oldest old” OR quinquagenarian* OR sexagenarian* OR septuagenarian* OR octogenarian* OR nonagenarian* OR frail* OR (functionally NEXT impair*) OR (older NEXT adult*) OR senior* OR retire* OR geriatric* OR
((50 OR 5565 OR 70 OR 75 OR 80 OR 85 OR 90 OR 95 OR 100) NEAR/3 (age OR aged OR ages OR old OR year*))
OR Aged
OR (advanced NEXT age*) OR “advancing years” OR ageing OR aging OR elder* OR gerontolog* OR (old NEXT adult*) OR (old NEXT age*) OR (rest NEXT home*) OR “very old” OR (nursing NEXT home*) OR ‘’geriatr*’’):ti,ab,kw
#9: #1 OR #2 OR #3 OR #4 OR #5 OR #6 OR #7 OR #8

**Concept 2: traumatic brain injury**
#10: [mh “Exoskeleton Device”]
#11: exoskeleton NEXT device OR robotic NEXT exoskeleton* OR exoskeleton:ti,ab,kw
#12: #10 OR #11
#13: #9 AND #12

**Cinahl**

**Concept 1: older adults**
(MH “Aged+”) OR (MH “Health Services for the Aged”) OR (MH ‘’Aged, Hospitalized”) OR (MH “Senior Centers”) OR (MR” Rehabilitation, Geriatric’’) OR (MH “Housing for the Elderly”) OR (MH “Gerontologic Nursing+”) OR (MH “Gerontologic Care”) OR (MH “Nursing Homes+”) OR (MH “Nursing Home Patients”) OR (MH “Frail Elderly”)
OR TI(“aged” OR “elderl*” OR ‘’oldest old’’ OR ‘’ quinquagenarian *’’ OR ‘’sexagenarian*’’ OR ‘’septuagenarian*’’ OR ‘’octogenarian*’’ OR ‘’nonagenarian*’’ OR ‘’ frail*’’ OR “functionally impair*” OR “older adult*” OR ‘’senior*’’ OR ‘’retire*’’ OR ‘’geriatric*’’ OR “advanced age*”OR “advancing years” OR ‘’ageing’’ OR ‘’aging’’ OR ‘’gerontolog*’’ OR “old adult*” OR “old age*” OR “rest home*” OR “very old” OR ‘’nursing home*’’ OR ‘’old age assistance’’ OR ‘’skilled nursing facilit*’’
OR ((50 OR 55 OR 65 OR 70 OR 75 OR 80 OR 85 OR 90 OR 95 OR 100) N3 (age OR ages OR aged OR old OR year*)))
OR AB(“aged” OR “elderl*” OR ‘’oldest old’’ OR ‘’ quinquagenarian *’’ OR ‘’sexagenarian*’’ OR ‘’septuagenarian*’’ OR ‘’octogenarian*’’ OR ‘’nonagenarian*’’ OR ‘’ frail*’’ OR “functionally impair*” OR “older adult*” OR ‘’senior*’’ OR ‘’retire*’’ OR ‘’geriatric*’’ OR “advanced age*”OR “advancing years” OR ‘’ageing’’ OR ‘’aging’’ OR ‘’gerontolog*’’ OR “old adult*” OR “old age*” OR “rest home*” OR “very old” OR ‘’nursing home*’’ OR ‘’old age assistance’’ OR ‘’skilled nursing facilit*’’
OR ((50 OR 55 OR 65 OR 70 OR 75 OR 80 OR 85 OR 90 OR 95 OR 100) N3 (age OR ages OR aged OR old OR year*)))

**Concept 2: exoskeletons**
(MH “Exoskeleton Device”) OR TI(“exoskeleton*” OR “Exoskeleton Device*” OR “robotic exoskeleton*”)OR AB(“exoskeleton*” OR “Exoskeleton Device*” OR “robotic exoskeleton*”)

**PEDro**

**Concept 1: older adults**
old*quinquagenarian*sexagenarian*septuagenarian*octogenarian*nonagenarian*frail* impair*seni*retire*geriatric*age*aging*elder*gerontolog*adult*rest*nursing*senior*

**Concept 2: exoskeletons**
exoskeleton*

**IEEE Xplore Digital Library**

**Concept 1: older adults**
oldest old OR quinquagenarian OR sexagenarian OR septuagenarian OR octogenarian OR nonagenarian OR frail OR functionally impair* OR older adult* OR senior OR retire* OR geriatric OR Aged OR advanced age* OR advancing years OR ageing OR aging OR elder OR gerontology* OR old adult OR old age* OR rest home OR very old OR nursing home* OR senior center

**Concept 2: exoskeletons**
exoskeleton OR Exoskeleton Device OR robotic exoskeleton

## Appendix B. Studies’ Quality Assessment Based on the Downs and Blacks Scale [31]

**Author (Year)**	**Reporting**										**External Validity**			**Internal Validity-Bias**							**Internal Validity—Confounding (Selection Bias)**						**P**	**T**	**Quality**
	**1**	**2**	**3**	**4**	**5**	**6**	**7**	**8**	**9**	**10**	**11**	**12**	**13**	**14**	**15**	**16**	**17**	**18**	**19**	**20**	**21**	**22**	**23**	**24**	**25**	**26**	**27**		
Aprigliano (2019) [32]	1	1	1	1	0	1	1	0	0	1	0	0	1	0	0	0	1	1	1	1	0	0	0	0	0	0	0	12	Poor
Calabrò (2018) [59]	1	1	1	1	1	1	1	1	0	1	1	1	1	1	1	1	1	1	1	1	1	1	1	1	1	0	1	25	Good
Carral (2022) [40]	1	1	1	1	0	1	0	0	0	0	1	1	1	0	0	0	1	1	1	1	1	1	0	0	0	0	1	15	Fair
Fang (2022) [33]	1	1	1	1	0	1	1	0	0	1	1	1	1	0	0	0	0	0	1	1	0	0	0	0	0	0	0	12	Poor
Firouzi (2022) [24]	1	1	1	1	0	1	1	1	0	0	1	1	1	0	0	0	1	1	1	1	0	0	0	0	0	1	0	15	Fair
Fujikawa (2022) [34]	1	1	1	2	0	1	0	0	0	0	1	1	1	0	0	0	0	1	1	1	0	0	0	0	0	0	0	12	Poor
Galle (2022) [35]	1	1	0	1	0	1	1	0	0	0	1	1	1	0	0	0	0	1	1	1	0	0	0	0	0	0	0	11	Poor
Gryfe (2022) [60]	1	1	1	1	0	1	1	0	0	1	1	1	1	0	0	0	1	1	1	1	1	1	1	1	1	1	1	21	Good
Jayaraman (2022) [36]	1	1	0	1	0	1	1	0	0	1	1	1	1	0	0	0	0	1	1	1	0	0	0	0	0	0	0	12	Poor
Jin (2017) [37]	1	1	1	1	0	1	1	0	0	0	1	1	1	0	0	0	0	1	1	1	1	1	0	0	0	0	0	14	Poor
Jin (2019) [41]	1	1	1	1	0	1	1	0	0	1	1	1	1	0	0	0	1	1	1	1	1	1	0	0	0	0	0	16	Fair
Kawashima (2022) [61]	1	1	1	1	0	1	1	0	0	1	1	1	1	0	0	0	1	1	1	1	1	1	1	1	0	1	1	20	Good
Koseki (2021) [42]	1	1	1	1	0	1	1	0	0	1	1	1	1	0	0	0	1	1	1	1	1	1	0	0	0	1	1	18	Fair
Lee (2017) [43]	1	1	1	1	0	1	1	0	0	0	1	1	1	0	0	0	1	1	1	1	1	1	0	0	0	0	0	15	Fair
Lee (2017) [44]	1	1	1	1	0	1	1	0	0	0	1	1	1	0	0	0	1	1	1	1	1	1	1	1	0	0	0	17	Fair
Lee (2022) [62]	1	1	1	1	0	1	1	0	0	0	1	1	1	0	1	0	1	1	1	1	1	1	1	1	1	1	1	21	Good
Lefeber (2018) [45]	1	1	1	1	0	1	1	0	0	0	1	1	1	0	0	0	1	1	1	1	1	1	0	0	0	0	1	16	Fair
Longatelli (2021) [46]	1	1	1	1	0	1	1	0	0	1	1	1	1	1	0	0	1	1	1	1	1	1	0	0	0	0	1	18	Fair
Martini (2019) [47]	1	1	1	1	0	1	1	0	0	0	1	1	1	0	0	0	1	1	1	1	1	1	1	1	1	0	1	19	Fair
Monaco (2017) [48]	1	1	1	1	0	1	1	0	0	1	1	1	1	0	0	0	1	1	1	1	0	1	0	0	0	0	0	15	Fair
Norris (2007) [49]	1	1	1	1	0	1	1	0	0	1	1	1	1	0	0	0	1	1	1	1	1	1	0	0	0	0	1	17	Fair
Panizzolo (2022) [50]	1	1	1	1	0	1	1	0	0	0	1	1	1	0	0	0	1	1	1	1	1	1	0	0	0	0	0	15	Fair
Park (2021) [51]	1	1	1	1	0	1	1	0	0	0	1	1	1	0	1	0	1	1	1	1	1	1	1	1	0	0	1	19	Fair
Roggeman (2022) [52]	1	1	1	1	0	1	1	0	0	1	1	1	1	0	0	0	1	1	1	1	1	1	0	0	0	0	1	17	Fair
Rojek (2020) [63]	1	1	1	1	0	1	1	0	0	1	1	1	1	0	0	0	1	1	1	1	1	1	1	1	1	1	1	21	Good
Romanato (2022) [38]	1	1	1	1	0	1	0	0	0	0	1	1	1	0	0	0	0	1	1	1	0	0	0	0	0	0	0	11	Poor
Setoguchi (2022) [53]	1	1	1	1	0	1	1	0	0	0	1	1	1	0	0	0	1	1	1	1	1	1	1	1	0	0	1	18	Fair
Shore (2022) [39]	1	1	0	1	0	1	0	0	0	0	1	1	1	0	0	0	0	1	1	1	1	1	0	0	0	0	1	13	Poor
Son (2021) [64]	1	1	1	1	0	1	0	0	0	1	1	1	1	1	0	0	1	1	1	1	1	1	1	1	0	1	1	20	Good
Taki (2020) [54]	1	1	1	1	0	1	1	0	0	1	1	1	1	0	0	0	1	1	1	1	1	1	0	0	0	1	1	18	Fair
Verrusio (2018) [55]	1	1	1	1	0	1	1	0	0	0	1	1	1	0	0	0	1	1	1	1	1	1	1	1	0	0	1	18	Fair
Watanabe (2017) [56]	1	1	0	1	0	1	1	0	1	1	1	1	1	0	0	0	0	1	1	1	1	1	1	1	0	1	1	19	Fair
Yeung (2021) [65]	1	1	1	1	1	1	1	1	0	0	1	1	1	0	1	0	1	1	1	1	1	1	1	1	1	0	1	22	Good
Yoshikawa (2018) [57]	1	1	1	1	0	1	1	0	0	1	1	1	1	0	0	0	1	1	1	1	1	1	0	0	0	0	1	17	Fair
Yoshimoto (2022) [66]	1	1	1	1	0	1	1	0	0	1	1	1	1	0	0	0	1	1	1	1	1	1	0	0	0	0	6	22	Good
Yun (2020) [58]	1	1	1	1	0	1	1	0	0	1	1	1	1	0	0	0	1	1	1	1	1	1	0	0	0	1	1	18	Fair
P = power; T = tot.																													

Downs and Blacks Scale questions (Downs and Black, 1998):
1. Is the hypothesis/aim/objective of the study clearly described?
2. Are the main outcomes to be measured clearly described in the Introduction or Methods section?
3. Are the characteristics of the patients included in the study clearly described?
4. Are the interventions of interest clearly described?
5. Are the distributions of principal confounders in each group of subjects to be compared clearly described?
6. Are the main findings of the study clearly described?
7. Does the study provide estimates of the random variability in the data for the main outcomes?
8. Have all important adverse events that may be a consequence of the intervention been reported?
9. Have the characteristics of patients lost to follow-up been described?
10. Have actual probability values been reported (e.g., 0.035 rather than <0.05) for the main outcomes except where the probability value is less than 0.001?
11. Were the subjects asked to participate in the study representative of the entire population from which they were recruited?
12. Were those subjects who were prepared to participate representative of the entire population from which they were recruited?
13. Were the staff, places, and facilities where the patients were treated, representative of the treatment the majority of patients receive?
14. Was an attempt made to blind study subjects to the intervention they have received?
15. Was an attempt made to blind those measuring the main outcomes of the intervention?
16. If any of the results of the study were based on “data dredging”, was this made clear?
17. In trials and cohort studies, do the analyses adjust for different lengths of follow-up of patients, or in case-control studies, is the time period between the intervention and outcome the same for cases and controls?
18. Were the statistical tests used to assess the main outcomes appropriate?
19. Was compliance with the intervention/s reliable?
20. Were the main outcome measures used accurate (valid and reliable)?
21. Were the patients in different intervention groups (trials and cohort studies) or were the cases and controls (case-control studies) recruited from the same population?
22. Were study subjects in different intervention groups (trials and cohort studies) or were the cases and controls (case-control studies) recruited over the same period of time?
23. Were study subjects randomized to intervention groups?
24. Was the randomized intervention assignment concealed from both patients and health care staff until recruitment was complete and irrevocable?
25. Was there adequate adjustment for confounding in the analyses from which the main findings were drawn?
26. Were losses of patients to follow-up taken into account?
27. Did the study have sufficient power to detect a clinically important effect where the probability value for a difference being due to chance is less than 5%?
All questions were scored on the following scale: yes = 1, unable to determine = 0, and no = 0.
