# Supplementary material for: A State-of-the-Art of Exoskeletons in Line with the WHO’s Vision on Healthy Aging: From Rehabilitation of Intrinsic Capacities to Augmentation of Functional Abilities
Source: Sensors (Basel). 2024 Mar 30;24(7):2230. doi: 10.3390/s24072230 (PMC11014060; doi:10.3390/s24072230)
Supplement: Supplementary file 1 [file sensors-24-02230-s001.zip › sensors-2918978-supplementary.pdf]

Supplementary material

Table S1. Summary of studies which used exoskeletons and assessed functional ability

Study (year)	N (n ≥65 years old)	Exoskeleton; supported movement	Study settings and design	Groups	Intervention	Outcome measures	Results
**Measure of mobility**							
**Parkinson's Disease**							
Gryfe et al. (2022) [60]	40 (NA)	Keeogo Rehab™; powered knee assistance	Hospital<br><br>RCT<br><br>T0: Baseline measurement<br><br>T1: Post-intervention (8 weeks)	Exoskeleton exercise (exo) (G1)	8 weeks of aerobic, strength and functional mobility exercises with the exoskeleton (16 sessions, 2 times a week)	Preferred gait speed (m/s)<br><br><br><br><br><br><br><br><br><br><br><br><br><br><br><br><br><br><br><br>FoG-Q (max 24)	G1: Exo<br><br>T0: 1.16 (±0.23)<br>T1: 1.21 (±0.19)<br>T0-T1: increase of 0.05 (±0.16) (P=0.274, P significance level = 0.017))<br><br>G2: Nxo<br><br>T0: 1.12 (±0.34)<br>T1: 1.15 (±0.33)<br>T0-T1: increase of 0.03 (±0.10) (P=0.240, P significance level = 0.017))<br><br>G3: Con<br><br>T0: 1.14 (±0.32)<br>T1: 1.16 (±0.35)<br>T0-T1: increase of 0.02 (±0.19) (P=0.735, P significance level = 0.017))<br><br>Between-groups comparison:<br>T0: (P=0.923)<br>T0-T1: (P=0.837)<br><br>G1:<br>T0: 7.6 (±5.7)<br>T1: 6.8 (±6.5)<br>T0-T1: decrease of 0.8 (±0.442) (P=0.442, P significance level = 0.017))<br><br>G2:<br>T0: 7.3 (±6.2)<br>T1: 6.8 (±5.1)<br>T0-T1: decrease of 0.5 (±3.3) (P=0.586, P significance level = 0.017))
				Non-exoskeleton exercise (Nxo) (G2)	8 weeks of aerobic, strength and functional mobility exercises without the exoskeleton (16 sessions, 2 times a week)		
				Wait-list control (Con) (G3)	No intervention		

							G3:<br>T0: 8.2 (±4.7)<br>T1: 8.8 (±5.0)<br>T0-T1: increase of 0.6 (±2.2) (P=0.337, P significance level = 0.017))<br><br>Between-groups comparison:<br>T0: (P=0.921)<br>T0-T1: (P=0.412)
						UPDRS - Motor functioning sub-scale (max 56)	G1:<br>T0: 13.5 (±6.7)<br>T1: 15.7 (±8.3)<br>T0-T1: increase of 2.2 (±4.1) (P=0.084, P significance level = 0.017))<br><br>G2:<br>T0: 13.4 (±10.0)<br>T1: 14.9 (±8.3)<br>T0-T1: increase of 1.5 (±5.2) (P=0.303, P significance level = 0.017))<br><br>G3:<br>T0: 16.0 (±7.6)<br>T1: 18.4 (±9.2)<br>T0-T1: increase of 2.4 (±5.2) (P=0.124, P significance level = 0.017))<br><br>Between-groups comparison:<br>T0: (P=0.668)<br>T0-T1: (P=0.805)
						PDQ-39 - Mobility sub-scale (max 100)	G1:<br>T0: 25.8 (±16.9)<br>T1: 19.2 (±14.5)<br>T0-T1: decrease of 6.5 (±8.3) (P=0.015, P significance level = 0.017))<br><br>G2:<br>T0: 28.5 (±23.1)<br>T1: 28.8 (±21.2)<br>T0-T1: increase of 0.4 (±13.6) (P=0.921, P significance level = 0.017))<br><br>G3:<br>T0: 35.4 (±14.9)<br>T1: 37.3 (±24.5)<br>T0-T1: increase of 1.9 (±14.1) (P=0.633, P significance level = 0.017))<br><br>Between-groups comparison:

							T0: (P=0.407)<br>T0-T1: (P=0.142)
Kawashima et al. (2022) [61]	12 (12)	Stride Management Assist exoskeleton (Honda R&D); Gait	Hospital<br><br>RCT<br><br>T0: Baseline measurement<br><br>T1: Post-intervention measurement (3 months)	SMA group (G1);	10 gait training sessions with the SMA (30 min each) for 3 months (step training and overground walking)<br><br>AND<br><br>Compare immediate effect with/without SMA during the 10MWT (first 6 times) (device off- to device on- mode)<br><br>I1= device off<br>I2= device on	Comparison of effects within- and between-groups after training intervention:<br><br>FoG-Q (max 24)	Values are mean (SEM)<br><br>G1:<br>T0= 12.2 (1.6) vs T1= 11.4 (1.7) (P=0.338)2<br>G2:<br>T0= 8.4 (1.2) vs T1= 10.6 (1.8) (P=0.239)<br><br>Between-group difference in change: P=0.183
				Control group (G2);	10 gait training sessions (30 min each) for 3 months (step training and overground walking) (**G2)**	10MWT (s)	G1:<br>T0= 12.5 (2.5) vs T1= 11.5 (2.4) (P=0.206)<br><br>G2:<br>T0= 13.7 (2.1) vs T1= 13.3 (1.7) (P=0.648)<br><br>Between-group difference in change: P=0.488
**Osteoarthritis**							
Koseki et al. (2021) [42]	21 (NA)	Honda Walking Assist; assist hip flexion and extension	Hospital<br><br>Quasi-experimental design;<br><br>T0: Pre- TKA measurement<br><br>T1: Week 2 following TKA<br><br>T2: Week 4 following TKA<br><br>T3: Week 8 following TKA	HWA group (G1);	Conventional rehabilitation + HWA gait training from week 1 to 5 after TKA<br>Total of 17-20 gait training sessions (each around 20-min), with 4-5 sessions per week	WOMAC-f	T0:<br>G1= 65.51 (±22.24 vs G2= 69.39 (±12.92) (P=0.623, ES= 0.21)<br><br>T1:<br>G1= 69.79 (±15.17) vs G2= 66.82 (±18.15) (P=0.682, ES=0.18)<br><br>T2:<br>G1= 87.17 (±8.63) vs G2= 81.95 (±10.71) (P=0.223, ES=0.54)<br><br>T3:<br>G1= 94.79 (±3.62) vs G2= 84.99 (±4.72) (P<0.001, ES=2.33)
				Control group (G2);	Conventional rehabilitation		

Setoguchi et al. (2022) [53]	47 (NA)	HAL;	Hospital<br><br>RCT<br><br>T0: Preoperatively<br><br>T1: 1 week postoperatively<br><br>T2: 3 weeks postoperatively	HAL group (G1);	HAL gait training: 3 sessions per week (40-min each) for 6 weeks in total + typical physical therapy on days without HAL training	Harris hip score:<br>- function	T0:<br>G1= 35.5 (±4.78) vs G2= 34.8 (±6.20) (P=0.832)<br><br>T2:<br>G1= 39.9 (±4.85) vs G2= 42.5 (±3.78) (P=0.336)<br><br>Within group changes:<br>G1:<br>T0-T2: P<0.05<br>G2:<br>T0-T2: P<0.05
				Control group (G2);	Typical physical therapy (40-min) each day	- motion	T0:<br>G1= 4.3 (±0.46) vs G2= 4.3 (±0.46) (P=1.000)<br><br>T2:<br>G1= 4.8 (±0.46) vs G2= 4.9 (±0.35) (P=0.535)<br><br>Within group changes:<br>G1:<br>T0-T2: P>0.05<br>G2:<br>T0-T2: P<0.05
						SF-36<br>- Physical functioning	T0:<br>G1= 15.0 (±7.33) vs G2= 13.6 (±20.58) (P=0.915)<br><br>T2:<br>G1= 43.4 (±10.21) vs G2= 35.3 (±11.86) (P=0.205)<br><br>Within group changes:<br>G1:<br>T0-T2: P<0.05<br>G2:<br>T0-T2: P<0.05
						- Role physical	T0:<br>G1= 25.0 (±4.26) vs G2= 28.7 (±16.99) (P=0.264)<br><br>T2:<br>G1= 43.3 (±9.85) vs G2= 42.4(±11.86) (P=0.873)<br><br>Within group changes:<br>G1:<br>T0-T2: P<0.05

							G2:<br>T0-T2: P=NS
Yoshikawa et al. (2018) [57]	19 (NA)	HAL; Gait	Hospital<br><br>Quasi-experimental design;<br><br>T0: Pre- TKA measurement<br>T1: Week 1 following TKA<br>T2: Week 2 following TKA<br>T3: Week 3 following TKA<br>T4: Week 4 following TKA<br>T5: Week 8 following TKA	HAL group (G1);	10-12 HAL training sessions (around 15-min each) over a 4 week period + conventional physical therapy (60-80 min a day)	WOMAC-f	T0:<br>G1= 82.2 (±16.9) vs G2= 74.6 (±15.0) (P=0.287)<br><br>T2:<br>G1= 82.8 (±14.5) vs G2= 69.6 (±19.0) (P=0.110)<br><br>T4:<br>G1= 86.5 (±10.6) vs G2= 83.3 (±11.3) (P=0.515)<br><br>T5:<br>G1= 92.6 (±6.6) vs G2= 85.3 (±5.7) (P=0.077)
				Control group (G2);	Conventional physical therapy (60-120 min a day)		
**Sarcopenia**							
Norris et al. (2007) [49]	7 (7)	PAFO; ankle plantarflexion assistance	Research center<br><br>Cross-sectional design	Older adults	Participants walked in three conditions:<br>I1= with standard shoes,<br>I2= with the PAFOs inactive,<br>I3= with the PAFOs active	Preferred walking speed (m/s)	I1= 1.08 (±0.20), I2= 1.03 (±0.16) and I3= 1.05 (±0.17)<br>I1 vs I2: P=0.098<br>I1 v I3: P=0.536<br>I2 vs I3: P=0.474
				Young adults (data not considered)	Participants walked in three conditions:<br>I1= with standard shoes,<br>I2= with the PAFOs inactive,<br>I3= with the PAFOs active		
**Hip fracture:**							
Fujikawa et al. (2022) [34]	14 (14)	HAL; hip support	Hospital<br><br>Within subject design;<br><br>T0: baseline, before HAL rehabilitation<br><br>T1: after HAL rehabilitation	No groups	Conventional rehabilitation + HAL rehabilitation (6x per week, for 15-min per session)	FTSS (sec)	T0 = 34.9 (±8.4) vs T1= 19.8 (±8.0) (P<0.01; ES= 1.81 (95% CI = 0.93 – 2.66))
**Stroke:**							

Taki et al. (2020) [54]	72 post-matched study patients (CRP group: n = 36 and HRP group: n = 36) (NA);	HAL; gait	Hospital<br><br>Observational study<br><br>T0= Time of rehabilitation admission<br><br>T1= Time of hospital discharge	CRP (G1);	Gait training with KAFO or AFO<br><br>3 hours/day for 7 days/week	FIM: motor subscore	T0: G1=19.64 (±11.19) vs G2=18.03 (±7.26) (P=446)<br><br>T1: G1=58.81 (±25.97) vs G2=69.67 (±17.37) (P=0.045)<br><br>T0-T1:<br>G1= increase of 39.17 (±24.37)<br>G2= increase of 51.64 (±16.72)<br>G1 vs G2 (P=0.013)
				HRP (G2);	Gait training with HAL<br><br>3 hours/day for 7 days/week	Locomotion at hospital ward on discharge:<br><br>- ambulation	G1=20 (55.6%) vs G2=30 (83.3%) (P=0.011)
Longatelli et al. (2021) [46]	29 (NA); 34.50 days [28–51] for the CG and 40 [30–64] days for the EG	Ekso GTA™; gait (hip and knee support)	Rehabilitation center<br><br>Pre-Post design<br><br>T0: Pre-intervention assessment<br><br>T1: Post-intervention assessment	CG (G1);	Standard therapy sessions<br><br>4 weeks (3 60-min sessions/week)	Capacity Score (include 6 clinical scales: 5-item Barthel Index, Motricity index, 10 MWT, 6 min walk test, FAC and Trunk Control Test)	Values are Median (I-III Quartile)<br><br>T0-T1:<br>G1: T0=5.06 (3.63-9.63) vs T1=9.06 (6.97-13.83) (P<0.001)<br>G2: T0=3.99 (3.29-5.50) vs T1=6.63 (5.76-7.61) (P<0.001)<br><br>T1:<br>G1=9.06 (6.97-13.83) vs G2=6.63 (5.76-7.61) (P=0.350)
				EG (15);	Standard therapy sessions + exoskeleton-assisted gait rehabilitation<br><br>4 weeks (12 sessions of 60 min of assisted rehabilitation 3 times/week + 8 sessions of conventional therapy)		
Watanabe et al. (2017) [56]	24 (HAL® group: n= 12, CGT group: n= 12) (NA); 57.0±44.3 days HAL group and 48.1±33.3 days in the control group	HAL; gait	Hospital<br><br>RCT;<br><br>T0: Pre-intervention<br><br>T1: Post-intervention (4 weeks)<br><br>T2: Follow-up 8 weeks<br><br>T3: Follow-up 12 weeks	HAL (G1);	Gait training using the HAL<br><br>3 times a week with a total of 12 sessions over 4 weeks	FAC	G1 x G2 (P=0.026)<br>G1: HAL group<br>T0: 2.0 (±1.0)<br>T1: 3.0 (±1.3)<br>T2: 3.5 (±1.5)<br>T3: 3.6 (±1.7)<br><br>G2: Conventional group<br>T0: 2.0 (±0.9)<br>T1: 2.5 (±1.3)<br>T2: 2.4 (±1.5)<br>T3: 2.7 (±1.6)
				CGT (G2);	Conventional gait training<br><br>3 times a week with a total of 12 sessions over 4 weeks	LE Fugl-Meyer Assessment	G1 x G2 (P=0.131)<br>G1: HAL group<br>T0: 19.5 (±6.3)<br>T1: 20.6 (±6.3)<br>T2: 18.7 (±7.0)<br>T3: 18.7 (±7.1)

							G2: Conventional group<br>T0: 21.1 (±5.4)<br>T1: 22.5 (±5.7)<br>T2: 22.7 (±6.4)<br>T3: 23.1 (±6.5)
Park et al. (2021) [51]	20 (NA); 13.20±7.20 days in the CPT-G and 7.60±4.9 days in the ICT-C group	Walkbot; Gait (hip, knee, and ankle support)	Hospital<br><br>RCT<br><br>T0: Pre-intervention<br><br>T1: Post-intervention	CPT-G (G1)	Conventional physical therapy and gait training<br><br>7 days/week, for 2 weeks. At least one 60-min session of physical therapy per day and the additional 30-min standard physical therapy session	FMA-LE synergy scale<br>- flexor synergy test (0-6)<br><br><br><br><br><br><br><br>- extensor synergy test (0-8)<br><br><br><br><br><br><br>- Total synergy	G1:<br>T0: 4.50 (±1.20) vs T1: 5.50 (±0.53)<br><br>G2:<br>T0: 3.86 (±1.83) vs T1: 5.29 (±0.99)<br><br>P (time effect)= 0.000 P (between groups)= 0.116<br>P (Time x Group)= 0.615<br><br>G1:<br>T0: 6.50 (±1.85) vs T1: 7.50 (±1.07)<br><br>G2:<br>T0: 5.71 (±2.16) vs T1: 7.07 (±1.00)<br><br>P (time effect)= 0.007<br>P (between groups)= 0.170<br>P (Time x Group)= 0.797<br><br>G1:<br>T0: 10.00 (±1.41) vs T1: 14.00 (±2.56)<br><br>G2:<br>T0: 9.14 (±2.25) vs T1: 12.79 (±2.64)
				ICT-C (G2)	Interlimb coordinated humanoid robot session with VR/AR game, combined with conventional physical therapy<br><br>7 days/week, for 2 weeks. At least one 60-min session of physical therapy per day and the additional 30-min ICT session		
Yeung et al. (2021) [65]	47 (NA); 27±17 days	Exoskeleton ankle robot; Ankle dorsiflexion and plantar flexion	Bi-center<br><br>RCT<br><br>T0: Pre-intervention<br><br>T1: Post-intervention	PAAR (G1) (N=14)	Conventional training routine (2h/weekday) + 30-min robot-assisted (Power-Assisted Ankle Robot) training (10-min stair training and two times 10-min overground walking)	FAC (max. 5)	T0:<br>G1= 1.9 (±0.7)<br>G2= 2.2 (±0.8)<br>G3= 2.2 (±1.0)<br><br>T0-T1 (mean difference [95% CI]):<br>G1= increase of 1.4 [1.0, 1.9] (P<0.001)<br>G2= increase of 1.4 [0.9, 2.0] (P<0.001)<br>G3= increase of 0.9 [0.4, 1.3] (P<0.01)
				SCAR (G2) (N=16)	Conventional training routine (2h/weekday) + 30-min robot-assisted (Swing-Controlled Ankle Robot) training (10-min		

					stair training and two times 10-min overground walking)		Between-group differences of score improvement (T0-T1):<br>G1 vs G3= 0.4 [-0.2, 1.0] (P=NS, ES=0.671)<br>G2 vs G3= 0.6 [0.0, 1.1] (P<0.05, ES=0.610)<br>G1 vs G2= -0.2 [-0.8, 0.4] (P=NS, ES=0.010)<br><br>T1: % independent walker (FAC ≥4)<br>G1= 57.1<br>G2= 56.3<br>G3= 29.4
				CT (G3) (N=17)	Conventional training routine (2h/weekday)		
**Healthy older adults**							
Jayaraman et al. (2022) [36]	12 (12)	GEMS-H; hip flexion and extension	Senior living community<br><br>Quasi-experimental study: Pre-Post test design<br><br>T0: Pre-intervention<br><br>T1: Post-intervention	No groups	Twelve gait training sessions (30min each) over a period of 4–6 weeks	FGA (scored on 30)<br><br>Number of sedentary bouts (>3min) per day<br><br>Time spent in the sedentary bouts (min/day)<br><br>FTSS (s)	T0: 17 (±2.9) vs T1: 23 (±3.6) (P< 0.001)<br><br>T0: 53.7 vs T1: 44.3 (P=0.004)<br><br>T0: 610.2 vs T1: 497.3 (P=0.003)<br><br>T0: 15.2 (±4.1) vs T1: 12.3 (±4.0) (P<0.001)
Fang et al. (2022) [33]	6 (6)	Dual-mode ankle exoskeleton; ankle plantar flexor assistance as a mobility aid and plantar flexor resistance as a functional muscle recruitment training platform.	Rehabilitation center<br><br>Within-subject design<br><br>Pilot subject:<br>T0: Pre resistance-training<br><br>T1: Post resistance-training	No groups	2 visits assessing 2 different protocols<br><br>Assistance protocol: treadmill waking in two conditions: exo-adaptation (I1) (30-min of bilateral plantarflexion and dorsiflexion assistance) and shod (I2) (no exoskeleton).<br><br>Resistance protocol: treadmill waking in two conditions: baseline (I3) (without exoskeleton) and resisted (I4) (bilateral plantar flexor resistance and biofeedback).<br><br>Resistance training protocol:	Resistance training protocol (N=1)<br><br>Self-selected walking speed (m/s)<br>Fast walking speed (m/s)	T0: 1.07 vs T1: 1.12<br><br>T0: 1.38 vs T1: 1.59

					One pilot participant performed 12 sessions of resistance training over four weeks (T0: Pre resistance-training, T1: Post resistance-training)		
Martini et al. (2019) [47]	20 (20)	APO; Hip flexion and extension	Hospital<br><br>Randomized Controlled Trial<br><br>T0: baseline measurement<br><br>T1: post-training program (end of week 4)<br><br>T2: follow-up measurement (one-month)	APO group (G1)	four-week robot-assisted gait training program, followed by a one-month follow-up period.	Daily steps (/10^3)	T0:<br>G1: 9.89 (±4.28) vs G2= 12.1 (±3.23) (P=0.35)<br><br>T1:<br>G1= 11.2 (±3.40) vs G2= 12.7 (±4.43) (P=0.39)<br><br>T2:<br>G1= 9.77 (±5.83) vs G2= 11.5 (±2.29) (P=0.16)
				Control group (G2)	four-week home exercise program of self-paced overground walking, followed by a one-month follow-up period.		
Lee et al. (2022) [62]	60 (60)	EX1; hip flexion and extension	Randomized controlled trial<br><br>T0: Pre intervention<br>T1: Post intervention	Group A (G1)	4 weeks of exercises with 3 sessions per week (40min each). Exercise consists of overground walking without exoskeleton at a comfortable speed for 300m straight.	SPPB	T0-T1 :<br>G1 : increase of 0.31 (±0.63) (P=NS)<br>G2 : increase of 0.33 (±0.82) (P=NS)<br>G3 : increase of 0.33 (±0.62) (P=NS)<br>G4 : increase of 0.87 (±1.19) (P<0.01)
				Group B (G2)	4 weeks of exercises with 3 sessions per week (40min each). Exercise consists of overground walking using the resistance mode of the exoskeleton at a comfortable speed for 300m straight.		
				Group C (G3)	4 weeks of exercises with 3 sessions per week (40min each). Exercise consists of stair ascent using the assistance mode of the exoskeleton at a comfortable speed from the first basement level to the fourth floor.		

				Group D (G4)	4 weeks of exercises with 3 sessions per week (40min each). Exercise consists of incline treadmill walking using the assistance mode of the exoskeleton at a comfortable speed.		
**Older person's ability to build/maintain social relations**							
**Osteoarthritis**							
Setoguchi et al. (2022) [53]	47 (NA)	HAL;	Hospital<br><br>RCT<br><br>T0: Preoperatively<br><br>T1: 1 week postoperatively<br><br>T2: 3 weeks postoperatively	HAL group (G1);	HAL gait training: 3 sessions per week (40-min each) for 6 weeks in total + typical physical therapy on days without HAL training	SF-36: Social functioning	T0:<br>G1= 36.9 (±12.14) vs G2= 36.9 (±17.37) (P=0.915)<br><br>T2:<br>G1= 52.2 (±9.59) vs G2= 51.4 (±9.39) (P=0.701)<br><br>Within group changes:<br>G1:<br>T0-T2: P=NS<br>G2:<br>T0-T2: P<0.05
				Control group (G2);	Typical physical therapy (40-min) each day		
**Ability to meet basic needs**							
**Stroke**							
Rojek et al. (2020) [63]	44 (NA); 4–12 months for the experimental group and 5-12 months in the control group	Ekso; gait	Rehabilitation center<br><br>RCT;<br><br>T0: pre-intervention<br><br>T1: post-intervention (4 weeks)	Experimental group (G1) (N=23);<br>;	Gait training with the Ekso GT exoskeleton, occupational therapy and individually selected physical therapy<br><br>5 times a week for 4 weeks	Barthel Index<br><br><br><br><br><br><br><br><br>Rivermead Mobility Index	G1: T0=25 (±25) vs T1=50 (±35) (P=0.00001)<br>G2: T0=85 (±25) vs T1=85 (±50) (P=0.006)<br><br>T0:<br>G1=25 (±25) vs G2=85 (±25) (P=0.0003)<br><br>T1:<br>G1=50 (±35) vs G2=85 (±50) (P=0.01)<br><br>G1: T0=3 (±5) vs T1=6 (±7) (P= 0.0003)<br>G2: T0=10 (±12) vs T1=13 (±8) (P=NS)<br><br>T0:<br>G1=3 (±5) vs G2=10 (±12) (P=NS)<br><br>T1:<br>G1=6 (±7) vs G2=13 (±8) (P=NS)
				Control group (G2) (N=21);	Classic rehabilitation: Individual exercises with a therapist, verticalization and gait, group exercises improving general fitness, occupational therapy and individually selected elements of physical therapy<br><br>5 times a week for 4 weeks		
Calabrò et al. (2018) [59]	40 (18 in EGT and 14 in OGT group); mean 10 ±3	Ekso; gait (hip and knee support)	Rehabilitation center<br><br>RCT	EGT (G1);	Conventional physiotherapy training + 45-min session of Ekso™ training + TMS	 <br>Rivermead Mobility Index	EGT-induced improvements:<br><br>ES=0.6, P=0.03

	m in the EGT and 11 ±3 m in the OGT		T0: Pre- gait training<br><br>T1: Post- gait training (8 weeks)		five 60-min sessions/ week for 8 consecutive weeks		
				OGT (G2);	Conventional physiotherapy training + 45-min of conventional gait training +TMS<br><br>five 60-min sessions/ week for 8 consecutive weeks		
Taki et al. (2020) [54]	72 post-matched study patients (CRP group: n = 36 and HRP group: n = 36) (NA);	HAL; gait	Hospital<br><br>Observational study	CRP (G1);	Gait training with KAFO or AFO<br><br>3 hours/day for 7 days/week	Modifed rankin scale	T0:<br>G1= 4 (4-5) vs G2= 4 (4-5) (Median (IQR)) (P=0.513)<br><br>T1: G1= 4 (3-4) vs G2= 4 (3-4) (Median (IQR)) (P=0.806)
				HRP (G2);	Gait training with HAL<br><br>3 hours/day for 7 days/week	FIM - Total	T0: G1=35.14 (±19.07) vs G2=35.92 (±15.8) (P=0.858)<br><br>T1: G1=84.47 (±33.0) vs G2=100.17 (±21.78) (P=0.024)<br><br>T0-T1:<br>G1= increase of 49.33 (±29.51)<br>G2= increase of 64.25 (±21.66)<br>G1 vs G2 (P=0.018)
**Parkinson's disease**							
Gryfe et al. (2022) [60]	40 (NA)	Keeogo Rehab^{TM}; powered knee assistance	Hospital<br><br>RCT<br><br>T0: Baseline measurement<br><br>T1: Post-intervention (8 weeks)	Exoskeleton exercise (G1)	8 weeks of aerobic, strength and functional mobility exercises with the exoskeleton (16 sessions, 2 times a week)	UPDRS - ADL sub-scale (max 52)	G1:<br>T0: 14.2 (±6.2)<br>T1: 14.5 (±5.4)<br>T0-T1: increase of 0.3 (±2.7) (P=0.687, P significance level = 0.017))<br><br>G2:<br>T0: 13.1 (±6.1)<br>T1: 13.0 (±7.7)<br>T0-T1: decrease of 0.1 (±3.5) (P=0.882, P significance level = 0.017))<br><br>G3:<br>T0: 13.8 (±6.0)<br>T1: 15.7 (±6.9)<br>T0-T1: increase of 1.9 (±3.8) (P=0.089, P significance level = 0.017))<br><br>Between-groups comparison:<br>T0: (P=0.910)<br>T0-T1: (P=0.265)
				Non-exoskeleton exercise (G2)	8 weeks of aerobic, strength and functional mobility exercises without the exoskeleton (16 sessions, 2 times a week)		
				Wait-list control (G3)	No intervention		

						PDQ-39 - ADL sub-scale (max 100)	G1:<br>T0: 18.3 (±11.6)<br>T1: 15.4 (±11.7)<br>T0-T1: decrease of 2.9 (±10.0) (P=0.318, P significance level = 0.017))<br><br>G2:<br>T0: 18.9 (±17.6)<br>T1: 19.2 (±18.1)<br>T0-T1: increase of 0.3 (±12.6) (P=0.928, P significance level = 0.017))<br><br>G3:<br>T0: 23.7 (±15.4)<br>T1: 29.5 (±23.7)<br>T0-T1: increase of 5.8 (±11.6) (P=0.098, P significance level = 0.017))<br><br>Between-groups comparison:<br>T0: (P=0.606)<br>T0-T1: (P=0.166)

Data in the results section are Mean (±SD), unless explicitly mentioned otherwise. P-level of significance = 0.05, unless explicitly mentioned otherwise.
N=number of participants; NA=information not available; NS= Not Significant; RCT: Randomized Controlled Trial; ES= effect size; CI= confidence interval; FoG-Q: Freezing of Gait Questionnaire; UPDRS: Unified Parkinson's Disease Rating Scale; PDQ-39: Parkinson's Disease Questionnaire-39; SEM= Standard Error of the Mean; 10MWT= 10 meter walk test; HWA: Honda Walking Assist; TKA: Total Knee Arthroplasty; WOMAC-f: Western Ontario and McMaster Universities Osteoarthritis Index – physical function subscale; HAL: Hybrid Assistive Limb; SF-36= 36-Item Short Form Health Survey; PAFO: Powered Ankle-Foot Orthosis; FTSS= five-times-sit-to-stand; CRP: Conventional Rehabilitation Program; HRP: HAL Rehabilitation Program; FIM= Functional Independence Measure; CG= Control Group; EG= Experimental Group; FAC= Functional Ambulation Category; CGT= Conventional Gait Training; FMA-LE: Fugl-Meyer Assessment of Lower Extremity; CPT-G= conventional physical therapy and gait training; ICT-C= interlimb coordinated humanoid robot combined with conventional physical therapy; SCAR: Swing-Controlled Ankle Robot; PAAR: Power-Assisted Ankle Robot; CT: Conventional Training; GEMS-H: Gait Enhancing and Motivating System for Hip; FGA= Functional Gait Assessment; APO= active pelvis orthosis; EX1= wearable robotic hip exoskeleton, developed at Samsung Electronics Co., Ltd. (Suwon, Republic of Korea); SPPB: Short Physical Performance Battery; EGT= Ekso Gait Training, OGT=Conventional Gait Training; TMS: Transcranial Magnetic Stimulation; IQR= interquartile range; ADL= activities of daily living

Table S2. Summary of studies which used exoskeletons and assessed intrinsic capacity

Study (year)	N (n ≥65 years old)	Exoskeleton; supported movement	Study design	Groups	Intervention	Outcome measures	Results
**Locomotor capacity**							
**Parkinson's Disease**							
Yun et al. (2020) [58]	11 (5)	Walkbot; gait (bilateral hip, knee and ankle)	Hospital<br><br>Quasi-experimental: Pre-Posttest design<br><br>T0: before treatment (N=11)<br><br>T1: immediately after treatment (week 4) (N=11)<br><br>T2: follow-up after treatment (one-month) (N=10)	No groups	4 weeks of exoskeleton use for gait training (45-min sessions, 3 days/week)	BBS	T0: 52.00 (±8.00)<br>T1: 54.00 (±4.00)<br>T2: 54.00 (±5.25)<br><br>T0-T1: increase (P=0.004)<br>T0-T2: increase (P=0.024)
Gryfe et al. (2022) [60]	40 (NA)	Keeogo Rehab™; powered knee assistance	Hospital<br><br>RCT<br><br>T0: Baseline measurement<br><br>T1: Post- intervention (8 weeks)	Exoskeleton exercise (G1)	8 weeks of aerobic, strength and functional mobility exercises with the exoskeleton (16 sessions, 2 times a week)	6MWT (m)	G1: Exo<br><br>T0: 374.4 (±78.9)<br>T1: 409.3 (±90.8)<br>T0-T1: increase of 34.8 (±17.6) (P<0.001, P significance level = 0.017))<br><br>G2: Nxo<br><br>T0: 369.3 (±122.0)<br>T1: 367.9 (±126.3)<br>T0-T1: decrease of 1.4 (±20.4) (P=0.822, P significance level = 0.017))<br><br>G3: Con<br><br>T0: 354.6 (±117.1)<br>T1: 350.9 (±117.0)<br>T0-T1: decrease of 3.8 (±40.5) (P=0.776, P significance level = 0.017))<br><br>Between-groups comparison:<br>T0: (P=0.914)
				Non-exoskeleton exercise (G2)	8 weeks of aerobic, strength and functional mobility exercises without the exoskeleton (16 sessions, 2 times a week)		
				Wait-list control (G3)	No intervention		

						B-BESTest (max 24)	T0-T1: (P=0.008)<br><br>G1: Exo<br><br>T0: 19.6 (±4.2)<br>T1: 19.0 (±4.1)<br>T0-T1: decrease of 0.6 (±2.1) (P=0.321, P significance level = 0.017))<br><br>G2: Nxo<br><br>T0: 17.5 (±5.8)<br>T1: 18.1 (±4.0)<br>T0-T1: increase of 0.6 (±3.0) (P=0.437, P significance level = 0.017))<br><br>G3: Con<br><br>T0: 18.5 (±4.4)<br>T1: 17.5 (±5.7)<br>T0-T1: decrease of 1.0 (±3.8) (P=0.357, P significance level = 0.017))<br><br>Between-groups comparison:<br>T0: (P=0.540)<br>T0-T1: (P=0.455)
Kawashima et al. (2022) [61]	12 (12)	SMA exoskeleton (Honda R&D); Gait	Hospital<br><br>RCT<br><br>T0: Baseline measurement<br><br>T1: Post- intervention measurement (3 months)	SMA group (G1);	10 gait training sessions with the SMA (30 min each) for 3 months (step training and overground walking)<br><br>AND<br><br>Compare immediate effect with/without SMA during the 10MWT (first 6 times) (device off- to device on-mode)<br><br>I1= device off<br>I2= device on	Comparison of effects within- and between- groups after training intervention:<br><br>3MWT (m)<br><br>BBS	Values = mean (SEM)<br><br>G1:<br>T0= 141.4 (14.4) vs T1= 154.7 (15.9) (P=0.023)<br><br>G2:<br>T0= 142.5 (17.9) vs T1= 142.1 (17.0) (P=0.949)<br><br>Between-group difference in change: P=0.109<br><br>G1:<br>T0= 49 (2.4) vs T1= 51.0 (2.2) (P=0.200)<br><br>G2:<br>T0= 49.4 (2.3) vs T1= 49.4 (2.1) (P=1.000)
				Control group (G2);	10 gait training sessions (30 min each) for 3		

					months (step training and overground walking) (**G2**)	FRT	Between-group difference in change: P=0.207<br><br>G1:<br>T0= 25.3 (2.0) vs T1= 25.7 (2.5) (P=0.785)<br><br>G2:<br>T0= 21.4 (3.2) vs T1= 20.1 (3.3) (P=0.626)<br><br>Between-group difference in change: P=0.609
**Neurological diseases:**							
Panizzolo et al. (2022) [50]	10 (NA)	Exoband; passive hip assistance	Rehabilitation center<br><br>Quasi-experimental: Pre-Post test design<br><br>T0: session 1 of wearing the exoband<br><br>T1: session 10 of wearing the exoband	No groups	10 walking sessions (10-min each) wearing the Exoband, for 5 consecutive weeks	Longest walking distance while wearing the Exoband (m)<br><br>Correlation between sessions spent walking with Exoband and meters covered	T0: 392.4 (±135.1) (95% CI: 295.7 – 489.1)<br>T1: 453.1 (±178.8) (95% CI: 325.2 – 581.0)<br>T0-T1: increase (P<0.05, ES: 0.38)<br><br>R= 0.9126; P<0.01; 95% CI: 0.66 - 0.98
**Osteoarthritis:**							
Koseki et al. (2021) [42]	21 (NA)	Honda Walking Assist; assist hip flexion and extension	Hospital<br><br>Quasi-experimental design;<br><br>T0: Pre- TKA measurement<br><br>T1: Week 2 following TKA<br><br>T2: Week 4 following TKA<br><br>T3: Week 8 following TKA	HWA group (G1);	Conventional rehabilitation + HWA gait training from week 1 to 5 after TKA<br><br>Total of 17-20 gait training sessions (each around 20-min), with 4-5 sessions per week	Range of motion knee:<br>Passive extension (°)<br><br>Active extension (°)	T0:<br>G1= -11.36 (±7.10) vs G2= -5.45 (±4.16) (P=0.027, ES= 1.02)<br><br>T1:<br>G1= -7.57 (±5.29) vs G2= -5.45 (±4.16) (P=0.310, ES=0.37)<br><br>T2:<br>G1= -3.64 (±3.93) vs G2= -3.64 (±3.23) (P=0.999, ES=0.00)<br><br>T3:<br>G1= -2.73 (±4.67) vs G2= -2.99 (±2.40) (P=0.870, ES=0.07)<br><br>T0:<br>G1= -12.73 (±7.20) vs G2= -6.36 (±5.52) (P=0.031, ES= 0.99)<br><br>T1:
				Control group (G2);	Conventional rehabilitation		

							G1= -11.05 (±5.70) vs G2= -12.27 (±5.18) (P=0.605, ES=0.23)<br><br>T2:<br>G1= -7.73 (±5.18) vs G2= -5.91 (±4.91) (P=0.408, ES=0.36)<br><br>T3:<br>G1= -5.00 (±5.00) vs G2= -6.37 (±6.35) (P=0.579, ES=0.24)
						Passive flexion (°)	T0:<br>G1= 116.82 (±9.82) vs G2= 123.19 (±16.17) (P=0.280, ES= 0.48)<br><br>T1:<br>G1= 95.17 (±10.48) vs G2= 101.82 (±9.02) (P=0.126, ES=0.68)<br><br>T2:<br>G1= 109.09 (±10.20) vs G2= 110.00 (±11.62) (P=0.847, ES=0.08)<br><br>T3:<br>G1= 115.45 (±8.50) vs G2= 117.21 (±9.49) (P=0.653, ES=0.20)
						Active flexion (°)	T0:<br>G1= 109.55 (±9.34) vs G2= 119.09 (±15.78) (P=0.103, ES= 0.74)<br><br>T1:<br>G1= 91.09 (±10.73) vs G2= 92.73 (±11.48) (P=0.733, ES=0.15)<br><br>T2:<br>G1= 103.64 (±12.06) vs G2= 102.27 (±14.89) (P=0.816, ES=0.10)<br><br>T3:<br>G1= 110.45 (±8.50) vs G2= 108.10 (±9.70) (P=0.552, ES=0.26)
						Maximum walking speed (m/s)	T0:<br>G1= 1.30 (±0.32) vs G2= 1.36 (±0.20) (P=0.583, ES= 0.23)

						Step length at maximum walking speed (m)<br><br><br><br><br><br><br><br><br><br><br><br>Cadence at maximum walking speed (steps/min)	T1:<br>G1= 1.20 (±0.21) vs G2= 0.90 (±0.35) (P=0.025, ES=1.04)<br><br>T2:<br>G1= 1.40 (±0.33) vs G2= 1.23 (±0.25) (P=0.403, ES=0.58)<br><br>T3:<br>G1= 1.46 (±0.29) vs G2= 1.44 (±0.21) (P=0.813, ES=0.08)<br><br><br>T0:<br>G1= 0.61 (±0.07) vs G2= 0.63 (±0.09) (P=0.584, ES= 0.25)<br><br>T1:<br>G1= 0.59 (±0.06) vs G2= 0.50 (±0.11) (P=0.036, ES=1.02)<br><br>T2:<br>G1= 0.65 (±0.07) vs G2= 0.58 (±0.09) (P=0.071, ES=0.87)<br><br>T3:<br>G1= 0.67 (±0.06) vs G2= 0.65 (±0.10) (P=0.679, ES=0.24)<br><br><br>T0:<br>G1= 127.50 (±22.38) vs G2= 130.61 (±11.50) (P=0.686, ES= 0.18)<br><br>T1:<br>G1= 122.70 (±13.15) vs G2= 110.21 (±26.62) (P=0.184, ES=0.60)<br><br>T2:<br>G1= 128.55 (±22.76) vs G2= 126.68 (±18.46) (P=0.834, ES=0.09)<br><br>T3:<br>G1= 131.02 (±19.99) vs G2= 132.30 (±13.53) (P=0.863, ES=0.08)
Yoshikawa et al. (2018) [57]	19 (NA)	HAL; Gait	Hospital<br><br>Quasi-experimental design;	HAL group (G1);	10-12 HAL training sessions (around 15-min each) over a 4 week period + conventional physical therapy (60-80 min a day)	Range of motion knee:<br>Passive extension (°)	T0:<br>G1= -4.0 (±8.4) vs G2= -6.4 (±5.0) (P=0.440)<br><br>T1:

			T0: Pre- TKA measurement<br><br>T1: Week 1 following TKA<br><br>T2: Week 2 following TKA<br><br>T3: Week 3 following TKA<br><br>T4: Week 4 following TKA<br><br>T5: Week 8 following TKA	Control group (G2);	Conventional physical therapy (60-120 min a day)		G1= -5.0 (±3.3) vs G2= -8.6 (±6.4) (P=0.117)<br><br>T2:<br>G1= -3.0 (±4.2) vs G2= -7.7 (±5.2) (P=0.034)<br><br>T3:<br>G1= -2.0 (±3.5) vs G2= -5.9 (±4.9) (P=0.051)<br><br>T4:<br>G1= -0.5 (±1.6) vs G2= -5.5 (±4.7) (P=0.006)<br><br>T5:<br>G1= -0.8 (±2.0) vs G2= -4.2 (±3.8) (P=0.086)
						Active extension (°)	T0:<br>G1= -7.0 (±5.4) vs G2= -6.8 (±5.6) (P=0.940)<br><br>T1:<br>G1= -10.5 (±7.6) vs G2= -13.2 (±7.2) (P=0.416)<br><br>T2:<br>G1= -5.0 (±5.3) vs G2= -12.3 (±5.2) (P=0.005)<br><br>T3:<br>G1= -4.0 (±3.9) vs G2= -7.7 (±4.1) (P=0.048)<br><br>T4:<br>G1= -3.5 (±4.1) vs G2= -6.4 (±6.0) (P=0.220)<br><br>T5:<br>G1= -2.5 (±2.7) vs G2= -5.8 (±3.8) (P=0.110)
						Passive flexion (°)	T0:<br>G1= 126.0 (±20.2) vs G2= 119.1 (±18.4)<br><br>T1:<br>G1= 95.3 (±16.9) vs G2= 95.5 (±7.6)<br><br>T2:<br>G1= 103.5 (±11.1) vs G2= 102.7 (±8.8)<br><br>T3:<br>G1= 109.5 (±9.8) vs G2= 108.2 (±9.3)

							T4:<br>G1= 115.8 (±9.2) vs G2= 110.9 (±10.9)<br><br>T5:<br>G1= 122.5 (±11.7) vs G2= 117.8 (±11.4)
						Active flexion (°)	T0:<br>G1= 123.0 (±22.4) vs G2= 115.0 (±17.0)<br><br>T1:<br>G1= 85.5 (±25.1) vs G2= 85.0 (±12.2)<br><br>T2:<br>G1= 96.0 (±14.3) vs G2= 94.1 (±10.9)<br><br>T3:<br>G1= 100.0 (±21.6) vs G2= 99.5 (±13.5)<br><br>T4:<br>G1= 108.0 (±15.1) vs G2= 104.1 (±13.0)<br><br>T5:<br>G1= 115.8 (±13.6) vs G2= 109.2 (±11.6)
						Walking ability: Maximum walking speed (m/s)	T0:<br>G1= 1.41 (±0.33) vs G2= 1.35 (±0.21) (P=0.588)<br><br>T2:<br>G1= 1.25 (±0.38) vs G2= 1.01 (±0.34) (P=0.137)<br><br>T4:<br>G1= 1.61 (±0.32) vs G2= 1.24 (±0.23) (P=0.006)<br><br>T5:<br>G1= 1.63 (±0.09) vs G2= 1.35 (±0.24) (P=0.027)
						Step length at maximum walking speed (m)	T0:<br>G1= 0.67 (±0.10) vs G2= 0.62 (±0.09) (P=0.274)<br><br>T2:<br>G1= 0.62 (±0.06) vs G2= 0.52 (±0.10) (P=0.016)

						Cadence at maximum walking speed (steps/min)	T4:<br>G1= 0.70 (±0.05) vs G2= 0.58 (±0.07) (P=0.001)<br><br>T5:<br>G1= 0.73 (±0.03) vs G2= 0.61 (±0.07) (P=0.003)<br><br>T0:<br>G1= 125.4 (±14.9) vs G2= 130.7 (±11.2)<br><br>T2:<br>G1= 120.0 (±25.3) vs G2= 114.0 (±28.4)<br><br>T4:<br>G1= 137.7 (±21.2) vs G2= 127.8 (±18.1)<br><br>T5:<br>G1= 133.9 (±10.0) vs G2= 133.4 (±20.6)
**Stroke:**							
Calabrò et al. (2018) [59]	40 (18 in EGT and 14 in OGT group); mean 10 ±3 m in the EGT and 11 ±3 m in the OGT	Ekso; gait (hip and knee support)	Rehabilitation center<br><br>RCT<br><br>T0: Pre- gait training<br><br>T1: Post- gait training (8 weeks)	EGT (G1);	Conventional physiotherapy training + 45-min session of Ekso™ training + TMS<br><br>five 60-min sessions/ week for 8 consecutive weeks	TUG (s)	EGT-induced improvements:<br><br>ES=0.5, P<0.02
				OGT (G2);	Conventional physiotherapy training + 45-min of conventional gait training +TMS<br><br>five 60-min sessions/ week for 8 consecutive weeks		
Rojek et al. (2020) [63]	44 (NA); 4–12 months for the experimental group and 5-12 months in the control group	Ekso; gait	Rehabilitation center<br><br>RCT;<br><br>T0: pre-intervention<br><br>T1: post-intervention (4 weeks)	Experimental group (G1) (N=23) ;	Gait training with the Ekso GT exoskeleton, occupational therapy and individually selected physical therapy<br><br>5 times a week for 4 weeks	Balance and load distribution with eyes open and closed (include COP path length, COP average velocity, length of minor and major	Both forms of rehabilitation resulted in significant changes in balance and functional status.<br>G1: improvement in variables related to sway area represented by the major and minor axes of an ellipse.<br>G2: increase of ellipse major and minor axes, improvement in sway velocity.<br><br>Following the therapy, both groups exhibited a slight and

				Control group (G2) (N=21);	Classic rehabilitation: Individual exercises with a therapist, verticalization and gait, group exercises improving general fitness, occupational therapy and individually selected elements of physical therapy<br><br>5 times a week for 4 weeks	axis, angle to major axis, deviation X and Y, forefoot load (un)involved, backfoot load (un)involved, and total load (un)involved)	insignificant trend towards reducing the total load distribution on their feet, particularly on the uninvolved limb. Notably, in the control group, there was a transfer of load from the backfoot to the forefoot.
Watanabe et al. (2017) [56]	24 (HAL® group: n= 12, CGT group: n= 12) (NA); 57.0±44.3 days HAL group and 48.1±33.3 days in the control group	HAL; gait	Hospital<br><br>RCT;<br><br>T0: Pre-intervention<br><br>T1: Post-intervention (4 weeks)<br><br>T2: Follow-up 8 weeks<br><br>T3: Follow-up 12 weeks	HAL (G1);	Gait training using the HAL<br><br>3 times a week with a total of 12 sessions over 4 weeks	Maximal walking speed (m/s)	G1 x G2 (P=0.975)<br>G1: HAL group<br>T0: 0.56 (±0.43)<br>T1: 0.85 (±0.43)<br>T2: 0.83 (±0.50)<br>T3: 0.84 (±0.51)<br><br>G2: Conventional group<br>T0: 0.45 (±0.53)<br>T1: 0.61 (±0.47)<br>T2: 0.54 (±0.35)<br>T3: 0.57 (±0.41)
				CGT (G2);	Conventional gait training<br><br>3 times a week with a total of 12 sessions over 4 weeks	6MWT (m)	G1 x G2 (P=0.810)<br>G1: HAL group<br>T0: 92.4 (±104.2)<br>T1: 156.7 (±137.8)<br>T2: 154.3 (±139.2)<br>T3: 166.7 (±143.9)<br><br>G2: Conventional group<br>T0: 106.9 (±132.6)<br>T1: 140.8 (±127.8)<br>T2: 142.7 (±102.1)<br>T3: 131.0 (±117.6)
						TUG (s)	G1 x G2 (P=0.413)<br>G1: HAL group<br>T0: 33.9 (±22.4)<br>T1: 16.7 (±6.9)<br>T2: 29.1 (±39.7)

							T3: 23.1 (±23.9)<br><br>G2: Conventional group<br>T0: 46.6 (±24.4)<br>T1: 28.6 (±17.9)<br>T2: 31.3 (±22.3)<br>T3: 27.3 (±18.9)
Yeung et al. (2021) [65]	47 (NA); 27±17 days	Exoskeleton ankle robot; Ankle dorsiflexion and plantar flexion	Bi-center<br><br>RCT<br><br>T0: Pre-intervention<br><br>T1: Post-intervention	PAAR (G1) (N=14)	Conventional training routine (2h/weekday) + 30-min robot-assisted (Power-Assisted Ankle Robot) training (10-min stair training and two times 10-min overground walking)	BBS (max. 56)	T0 (mean (±SD):<br>G1= 24.0 (±11.3)<br>G2= 30.7 (±14.6)<br>G3= 25.9 (±14.4)<br><br>T0-T1 (mean difference [95% CI]):<br>G1= increase of 18.8 [13.1, 24.4] (P<0.001)<br>G2= increase of 12.6 [6.2, 18.9] (P<0.01)<br>G3= increase of 14.4 [9.4, 19.3] (P<0.001)<br><br>Between-group differences of score improvement (T0-T1):<br>G1 vs G3= 3.6 [-2.7, 9.9] (P=NS, ES=0.458)<br>G2 vs G3= 0.3 [-5.8, 6.5] (P=NS, ES=0.166)<br>G1 vs G2= 3.2 [-3.3, 9.7] (P=NS, ES=0.567)
				SCAR (G2) (N=16)	Conventional training routine (2h/weekday) + 30-min robot-assisted (Swing-Controlled Ankle Robot) training (10-min stair training and two times 10-min overground walking)		
				CT (G3) (N=17)	Conventional training routine (2h/weekday)		
**Hip fracture**							
Fujikawa et al. (2022) [34]	14 (14)	HAL; hip support	Hospital<br><br>Within subject design;<br><br>T0: baseline, before HAL rehabilitation<br><br>T1: after HAL rehabilitation	No groups	Conventional rehabilitation + HAL rehabilitation (6x per week, for 15-min per session)	TUG (sec)	Individual participants (case):<br>1: T0= 15.2 vs T1= 8.2<br>2: T0= 48.7 vs T1= 25.4<br>3: T0= 101.2 vs T1= 18.9<br>4: T0= 16.3 vs T1= 11.1<br>7: T0= 72.0 vs T1= 19.4<br>8: T0= 59.7 vs T1= 324<br>13: T0= 32.9 vs T1= 29.7
**Healthy older adults**							
Jayaraman et al. (2022) [36]	12 (12)	GEMS-H; hip flexion and extension	Senior living community<br><br>Quasi-experimental study: Pre-Post test design	No groups	Twelve gait training sessions (30min each) over a period of 4–6 weeks	10MWT (m/s)<br>- Fasted safe gait speed<br><br>6MWT (m)	T0 vs T1: improvement of 0.21 m/s (P= 0.001)<br><br>T0: 371.2 m (±94.5) vs T1 : 433.7 m (± 113.1) (P<0.001)

			T0: Pre-intervention<br><br>T1: Post-intervention			BBS	T0: 48 out of 56 (±7) vs T1: 52 out of 56 (±6) (P< 0.001)
Aprigliano et al. (2019) [32]	6 (6)	APO; Hip flexion and extension	Rehabilitation center<br><br>Within-subject design	No groups	1 session<br><br>14 experimental trials with the APO. These trials include perturbations (sagittal and frontal plane) and fake trials (no perturbation) with two robot-working modalities. The "zero-torque mode" allows the subject to move freely without being obstructed, while the "assistive mode" will supply torques at both hip joints when balance loss is detected to promote stability recovery.	Balance recovery after perturbation while walking:<br><br>Margin of Stability (MoS), defined as the person´s ability to manage unexpected perturbations while walking:<br><br>- Sagittal plane<br>- Frontal plane	The findings indicate that the assistive approach effectively enhances balance recovery in the sagittal plane for both perturbation paradigms. However, it does not demonstrate effectiveness in maintaining stability in the frontal plane
Fang et al. (2022) [33]	6 (6)	Dual-mode ankle exoskeleton; ankle plantar flexor assistance as a mobility aid and plantar flexor resistance as a functional muscle recruitment training platform.	Rehabilitation center<br><br>Within-subject design<br><br>Pilot subject:<br>T0: Pre resistance-training<br><br>T1: Post resistance-training	No groups	2 visits assessing 2 different protocols<br><br>Assistance protocol: treadmill waking in two conditions: exo-adaptation (I1) (30-min of bilateral plantarflexion and dorsiflexion assistance) and shod (I2) (no exoskeleton).<br><br>Resistance protocol: treadmill waking in two conditions: baseline (I3) (without exoskeleton) and resisted (I4) (bilateral plantar flexor resistance and biofeedback).<br><br>Resistance training protocol:	*Resistance training protocol (N=1)*<br><br>6MWT distance (m)<br><br>Plantar flexor strength<br><br>Fast walking speed (m/s)	 <br>T0: 397 to T1: 539 (35% increase)<br><br>T0 vs T1: increase of 18% (right side) and 43% (left side)<br><br>T0: 1.38 vs T1: 1.59

					One pilot participant performed 12 sessions of resistance training over four weeks (T0: Pre resistance-training, T1: Post resistance-training		
Lee et al. (2022) [62]	60 (60)	EX1; hip flexion and extension	Rehabilitation center<br><br>RCT<br><br>T0: Pre intervention<br>T1: Post intervention	Group A (G1)	4 weeks of exercises with 3 sessions per week (40min each). Exercise consists of overground walking without exoskeleton at a comfortable speed for 300m straight.	BBS<br><br><br><br><br><br>TUG (sec)<br><br><br><br><br><br>FRT (cm)<br><br><br><br><br><br>Muscle strength	T0-T1 :<br>G1 : 2.46 (±2.03) (P<0.01)<br>G2 : 2.46 (±1.69) (P<0.01)<br>G3 : 2.95 (±2.47) (P<0.01)<br>G4 : 3.93 (±2.15) (P<0.01)<br><br>T0-T1 :<br>G1 : 0.56 (±1.05) (P=NS)<br>G2 : 1.18 (±0.95) (P<0.01)<br>G3 : 0.73 (±0.81) (P<0.01)<br>G4 : 0.58 (±0.61) (P<0.01)<br><br>T0-T1 :<br>G1 : 0.46 (±2.56) (P=NS)<br>G2 : 1.12 (±5.47) (P=NS)<br>G3 : 2.45 (±6.13) (P=NS)<br>G4 : 3.38 (±4.18) (P<0.01)<br><br>T0-T1:<br>G1: increase in muscle strength of knee flexion (P<0.05) and knee extension (P<0.01)<br>G2: increase in muscle strength of trunk flexion (P<0.01), hip flexion (P<0.05), hip extension (P<0.01), hip abduction (P<0.01), knee flexion (P<0.05), knee extension (P<0.01), ankle dorsiflexion (P<0.05), and ankle plantar flexion (P<0.01)<br>G3: increase in muscle strength of trunk flexion (P<0.05), hip adduction (P<0.01), knee extension (P<0.05), and ankle plantarflexion (P<0.05)<br>G4: increase in muscle strength of trunk extension (P<0.01), hip flexion (P<0.05), hip extension (P<0.01), and knee extension (P<0.01)
				Group B (G2)	4 weeks of exercises with 3 sessions per week (40min each). Exercise consists of overground walking using the resistance mode of the exoskeleton at a comfortable speed for 300m straight.		
				Group C (G3)	4 weeks of exercises with 3 sessions per week (40min each). Exercise consists of stair ascent using the assistance mode of the exoskeleton at a comfortable speed from the first basement level to the fourth floor.		
				Group D (G4)	4 weeks of exercises with 3 sessions per week (40min each). Exercise consists of incline treadmill walking using the assistance mode of the exoskeleton at a comfortable speed.		
**Vitality capacity**							
**Parkinson's Disease**							

Kawashima et al. (2022) [61]	12 (12)	Stride Management Assist exoskeleton (Honda R&D); Gait	Hospital<br><br>RCT<br><br>T0: Baseline measurement<br><br>T1: Post- intervention measurement (3 months)	SMA group (G1);	10 gait training sessions with the SMA (30 min each) for 3 months (step training and overground walking)<br><br>AND<br><br>Compare immediate effect with/without SMA during the 10MWT (first 6 times) (device off- to device on-mode)<br>I1= device off<br>I2= device on	Comparison of effects within- and between- groups after training intervention:<br><br>PCI in 3MWT (beats/min)	G1:<br>T0= 0.29 (0.08) vs T1= 0.13 (0.05) (P=0.046)<br><br>G2:<br>T0= 0.34 (0.13) vs T1= 0.35 (0.14) (P=0.850)<br><br>Between-group difference in change: P=0.147
				Control group (G2);	10 gait training sessions (30 min each) for 3 months (step training and overground walking)		
**Osteoarthritis**							
Koseki et al. (2021) [42]	21 (NA)	Honda Walking Assist; assist hip flexion and extension	Hospital<br><br>Quasi-experimental design;<br><br>T0: Pre- TKA measurement<br><br>T1: Week 2 following TKA<br><br>T2: Week 4 following TKA<br><br>T3: Week 8 following TKA	HWA group (G1);	Conventional rehabilitation + HWA gait training from week 1 to 5 after TKA<br><br>Total of 17-20 gait training sessions (each around 20-min), with 4-5 sessions per week	Knee extension torque (Nm/kg)	T0:<br>G1= 0.88 (±0.21) vs G2= 0.82 (±0.28) (P=0.543, ES= 0.24)<br><br>T1:<br>G1= 0.51 (±0.16) vs G2= 0.56 (±0.17) (P=0.448, ES=0.30)<br><br>T2:<br>G1= 0.78 (±0.31) vs G2= 0.77 (±0.20) (P=0.928, ES=0.04)<br><br>T3:<br>G1= 0.97 (±0.32) vs G2= 0.91 (±0.20) (P=0.629, ES=0.23)
				Control group (G2);	Conventional rehabilitation	Knee flexion torque (Nm/kg)	T0:<br>G1= 0.43 (±0.13) vs G2= 0.46 (±0.21) (P=0.721, ES= 0.17)<br><br>T1:<br>G1= 0.32 (±0.10) vs G2= 0.34 (±0.09) (P=0.687, ES=0.21)<br><br>T2:<br>G1= 0.42 (±0.14) vs G2= 0.39 (±0.16) (P=0.610, ES=0.20)<br><br>T3:<br>G1= 0.50 (±0.15) vs G2= 0.49 (±0.13) (P=0.879, ES=0.07)

Yoshikawa et al. (2018) [57]	19 (NA)	HAL; Gait	Hospital<br><br>Quasi-experimental design;<br><br>T0: Pre- TKA measurement<br><br>T1: Week 1 following TKA<br><br>T2: Week 2 following TKA<br><br>T3: Week 3 following TKA<br><br>T4: Week 4 following TKA<br><br>T5: Week 8 following TKA	HAL group (G1);	10-12 HAL training sessions (around 15-min each) over a 4 week period + conventional physical therapy (60-80 min a day)	Knee extension torque (Nm/kg)<br><br><br><br><br><br><br><br><br><br><br><br>Knee flexion torque (Nm/kg)	T0:<br>G1= 1.10 (±0.64) vs G2= 0.91 (±0.31) (P=0.373)<br><br>T2:<br>G1= 0.68 (±0.43) vs G2= 0.67 (±0.23) (P=0.924)<br><br>T4:<br>G1= 0.93 (±0.32) vs G2= 0.85 (±0.23) (P=0.541)<br><br>T5:<br>G1= 1.15 (±0.12) vs G2= 0.88 (±0.17) (P=0.014)<br><br>T0:<br>G1= 0.56 (±0.22) vs G2= 0.51 (±0.22)<br><br>T2:<br>G1= 0.36 (±0.15) vs G2= 0.41 (±0.15)<br><br>T4:<br>G1= 0.45 (±0.16) vs G2= 0.41 (±0.15)<br><br>T5:<br>G1= 0.51 (±0.12) vs G2= 0.50 (±0.15)
				Control group (G2);	Conventional physical therapy (60-120 min a day)		
**Psychological capacity**							
**Parkinson's Disease**							
Yun et al. (2020) [58]	11 (5)	Walkbot; gait (bilateral hip, knee and ankle)	Hospital<br><br>Quasi-experimental: Pre-Posttest design<br><br>T0: before treatment (N=11)<br><br>T1: immediately after treatment (week 4) (N=11)<br><br>T2: follow-up after treatment (one-month) (N=10)	No groups	4 weeks of exoskeleton use for gait training (45-min sessions, 3 days/week)	KFES	T0: 28.00 (±9.00)<br>T1: 30.00 (±13.00)<br>T2: 32.50 (±15.75)<br><br>T0-T1: increase (P=0.235)<br>T0-T2: increase (P=0.086)
Gryfe et al. (2022) [60]	40 (NA)	Keeogo Rehab™; powered knee assistance	RCT<br><br>T0: Baseline measurement	Exoskeleton exercise (Exo);	8 weeks of aerobic, strength and functional mobility exercises with	ABC	G1:<br>T0: 81.1 (±14.5)<br>T1: 83.8 (±14.3)

			T1: Post- intervention (8 weeks)	G1	the exoskeleton (16 sessions, 2 times a week)		T0-T1: increase of 2.8 (±8.2) (P=0.246, P significance level = 0.017))<br><br>G2:<br>T0: 72.5 (±24.2)<br>T1: 74.8 (±20.2)<br>T0-T1: increase of 2.3 (±15.2) (P=0.576, P significance level = 0.017))<br><br>G3:<br>T0: 71.2 (±16.1)<br>T1: 70.1 (±19.9)<br>T0-T1: decrease of 1.1 (±16.3) (P=0.819, P significance level = 0.017))<br><br>Between-groups comparison:<br>T0: (P=0.361)<br>T0-T1: (P=0.429)
				Non-exoskeleton exercise (Nxo);<br><br>G2	8 weeks of aerobic, strength and functional mobility exercises without the exoskeleton (16 sessions, 2 times a week)		
				Wait-list control (Con);<br><br>G3	No intervention		
**Depression**							
Verrusio et al. (2018) [55]	20 (NA)	Human Body Posturizer;	Nursing home<br><br>RCT<br><br>T0: baseline measurement<br>T1: measurement after the 6 month intervention period	HBP group;<br><br>G1	3 sessions of physical exercise training with the HBP a week (each session around 45-min) for 6 months in total	GDS	G1:<br>T0= 8.5 (±1.17) vs T1= 5.2 (±1.1) (P<0.01)<br><br>G2:<br>T0= 8.6 (±1.26) vs T1= 7.9 (±1.0) (P=NS)
				Exercise group;<br><br>G2	3 sessions of physical exercise training a week (each session around 45-min) for 6 months in total		
**Healthy older adults**							
Lee et al. (2022) [62]	60 (60)	EX1; hip flexion and extension	Rehabilitation center<br><br>Randomized controlled trial<br><br>T0: Pre intervention<br>T1: Post intervention	Group A (G1)	4 weeks of exercises with 3 sessions per week (40min each). Exercise consists of overground walking without exoskeleton at a comfortable speed for 300m straight.	GDS-SF	T0-T1 :<br>G1 : 0.16 (±1.77) (P=NS)<br>G2 : 1.07 (±1.49) (P<0.05)<br>G3 : 1.00 (±2.18) (P=NS)<br>G4 : 1.40 (±2.03) (P<0.05)
				Group B (G2)	4 weeks of exercises with 3 sessions per week (40min each). Exercise consists of overground		

					walking using the resistance mode of the exoskeleton at a comfortable speed for 300m straight.		
				Group C (G3)	4 weeks of exercises with 3 sessions per week (40min each). Exercise consists of stair ascent using the assistance mode of the exoskeleton at a comfortable speed from the first basement level to the fourth floor.		
				Group D (G4)	4 weeks of exercises with 3 sessions per week (40min each). Exercise consists of incline treadmill walking using the assistance mode of the exoskeleton at a comfortable speed.		
**Cognitive capacity**							
**Parkinson's Disease**							
Yun et al. (2020) [58]	11 (5)	Walkbot; gait (bilateral hip, knee and ankle)	Hospital<br><br>Quasi-experimental: Pre-Posttest design<br><br>T0: before treatment (N=11)<br><br>T1: immediately after treatment (week 4) (N=11)<br><br>T2: follow-up after treatment (one-month) (N=10)	No groups	4 weeks of exoskeleton use for gait training (45-min sessions, 3 days/week)	Percentage (%) of dual-task interference: gait velocity (10 MWT):<br>- dual-task cognitive<br><br><br><br><br><br>- dual-task physical	T0: -15.78 (±7.78)<br>T1: -21.50 (±7.62)<br>T2: -20.75 (±6.40)<br><br>T0-T1: increase (P=0.026)<br>T0-T2: increase (P=0.203)<br><br>T0: -21.23 (±7.42)<br>T1: -21.10 (±5.79)<br>T2: -23.51 (±12.55)<br><br>T0-T1: increase (P=0.929)<br>T0-T2: increase (P=0.646)

Data in the results section are Mean (±SD), unless explicitly mentioned otherwise. P-level of significance = 0.05, unless explicitly mentioned otherwise.
N=number of participants; NA=information not available; NS= Not Significant; BBS=Berg Balance Scale; RCT=Randomized Controlled Trial; 6MWT= 6 minute walk test; B-BESTest= Brief-Balance Evaluation Systems Test; SEM= Standard Error of the Mean; SMA= Stride Management Assist exoskeleton; 3MWT=3-minute walk test; FRT= Functional Reach Test; ES=effect size; CI= confidence interval; HWA=Honda Walking Assist; TKA=total knee arthroplasty; HAL=Hybrid Assistive Limb; TMS: Transcranial Magnetic Stimulation; EGT= Ekso Gait Training, OGT=Conventional Gait Training; TUG= Timed Up & Go test; COP: Center of Pressure; MoS: Margin of Stability; APO: active pelvis orthosis; SCAR: Swing-Controlled Ankle Robot; PAAR: Power-Assisted Ankle Robot; CT: Conventional Training; GEMS-H: Gait Enhancing and Motivating System for Hip; FRT: Functional Reach Test; EX1= wearable robotic hip exoskeleton, developed at Samsung Electronics Co., Ltd. (Suwon, Republic of Korea); PCI = Physiological cost index; ABC= Activities-specific Balance Confidence; GDS= Geriatric Depression Scale; GDS-SF= Geriatric Depression Scale (short form)

Table S3. Summary of studies which used exoskeletons and assessed performance indicators

Study (year)	N (n ≥65 years old)	Exoskeleton; supported movement	Study settings and design	Groups	Intervention	Outcome measures	Results
**Locomotor performance indicators**							
**Parkinson's Disease**							
Romanato et al. (2022) [38]	5 (NA)	EksoGT; gait	Rehabilitation center<br><br>Quasi-experimental: Pre-Posttest design<br><br>T0: Pre- exoskeleton training<br><br>T1: Post- exoskeleton training	People with PD	4 week of overground wearable exoskeleton training	Muscle forces during walking (% of body weight)<br>- Anterior kinetic chain<br><br><br><br><br><br><br>- Posterior kinetic chain	T0 vs T1:<br><br>Terminal stance: increase (P<0.05)<br>Early swing: increase (P<0.05)<br><br><br>T0 vs T1:<br><br>Terminal stance: increase (P<0.05)<br>Early swing: increase (P<0.05)
				Healthy controls (data not considered)	No intervention applied		
Yun et al. (2020) [58]	11 (5)	Walkbot; gait (bilateral hip, knee and ankle)	Hospital<br><br>Quasi-experimental: Pre-Posttest design<br><br>T0: before treatment (N=11)<br><br>T1: immediately after treatment (week 4) (N=11)<br><br>T2: follow-up after treatment (one-month) (N=10)	No groups	4 weeks of exoskeleton use for gait training (45-min sessions, 3 days/week)	10 MWT (comfortable gait speed, m/s):<br>- single task condition<br><br><br><br><br><br><br>- dual-task condition (cognitive)<br><br><br><br><br><br><br>- dual-task condition (physical)	 <br>T0: 1.13 (±0.23)<br>T1: 1.24 (±0.28)<br>T2: 1.17 (±0.34)<br><br>T0-T1: increase (P=0.041)<br>T0-T2: increase (P=0.445)<br><br>T0: 0.94 (±0.25)<br>T1: 0.98 (±0.24)<br>T2: 0.92 (±0.26)<br><br>T0-T1: increase (P=1.000)<br>T0-T2: increase (P=0.721)<br><br>T0: 0.89 (±0.22)<br>T1: 0.98 (±0.23)<br>T2: 0.90 (±0.29)<br><br>T0-T1: increase (P=0.075)<br>T0-T2: increase (P=0.721)
Kawashima et al. (2022) [61]	12 (12)	Stride Management Assist exoskeleton (Honda R&D); Gait	Hospital<br><br>RCT	SMA group (G1);	10 gait training sessions with the SMA (30 min each) for 3 months (step training and overground walking)	Immediate effect of the SMA during 10 MWT:	Values = mean (SEM)<br><br>G1: SMA group

			T0: Baseline measurement<br><br>T1: Post- intervention measurement (3 months)		AND<br><br>Compare immediate effect with/without SMA during the 10MWT (first 6 times) (device off- to device on- mode)<br><br>I1= device off<br>I2= device on	Walking speed (m/s)	I1= 0.85 (0.05) vs I2= 0.95 (0.06) (P=0.000)
						Step length (m)	I1= 0.472 (0.023) vs I2= 0.524 (0.022) (P=0.000)
						Cadence (steps/min)	I1= 106.3 (2.4) vs I2= 106.6 (2.7) (P=0.800)
				Control group (G2);	10 gait training sessions (30 min each) for 3 months (step training and overground walking) **(G2)**	Range of flexion mobility of left thigh (°)	I1= 19.3 (1.3) vs I2= 27.8 (1.5) (P=0.000)
						Range of flexion mobility of right thigh (°)	I1= 21.5 (1.3) vs I2= 29.2 (1.7) (P=0.000)
						Range of extension mobility of left thigh (°)	I1= -5.4 (0.8) vs I2= -8.7 (1.0) (P=0.000)
						Range of extension mobility of right thigh (°)	I1= -5.5 (0.8) vs -9.9 (1.1) (P=0.000)
						Symmetry of mobility between both thighs	I1= 0.8 (0.03) vs I2= 0.9 (0.01) (P=0.334)
						Range of scissor angle on left thigh (°)	I1= 24.8 (1.5) vs I2= 37.8 (1.5) (P=0.000)
						Range of scissor angle on right thigh (°)	I1= 26.9 (1.6) vs I2= 37.9 (2.0) (P=0.000)
						Symmetry of scissor angle between both thighs	I1= 0.8 (0.03) vs I2= 0.9 (0.02) (P=0.109)
						Comparison of effects within- and between-groups after training intervention:	
						10MWT (s)	G1:<br>T0= 12.5 (2.5) vs T1= 11.5 (2.4) (P=0.206)<br><br>G2:<br>T0= 13.7 (2.1) vs T1= 13.3 (1.7) (P=0.648)

							Between-group difference in change:<br>P=488
**Osteoarthritis:**							
Setoguchi et al. (2022) [53]	47 (NA)	HAL;	Hospital<br><br>RCT<br><br>T0: Preoperatively<br><br>T1: 1 week postoperatively<br><br>T2: 3 weeks postoperatively	HAL group (G1);<br><br>Control group (G2);	HAL gait training (first-third weeks post-surgery): 3 sessions per week (40-min each) for 6 sessions in total + typical physical therapy on days without HAL training<br><br>Typical physical therapy (40-min) each day	Sagittal hip motion:<br>- flexion (°)<br><br>- extension (°)<br><br>- full ROM (°)	T0:<br>G1= 30.1 vs G2= 29.6 (P=NS)<br><br>T1:<br>G1= 31.1 vs G2= 31.3 (P=NS)<br><br>T2:<br>G1= 32.4 vs G2= 32.1 (P=NS)<br><br>Within group changes:<br>G1: P=NS<br>G2: P=NS<br><br>T0:<br>G1= -8.8 vs G2= -7.5 (P=NS)<br><br>T1:<br>G1= -12.1 vs G2= -11.6 (P=NS)<br><br>T2:<br>G1= -3.2 vs G2= -7.9 (P<0.05)<br><br>Within group changes:<br>G1: P=NS<br>G2: P=NS<br><br>T0:<br>G1= 21.2 vs G2= 22.2 (P=NS)<br><br>T1:<br>G1= 19 vs G2= 19.7 (P=NS)<br><br>T2:<br>G1= 29.1 vs G2= 24.2 (P<0.05)<br><br>Within group changes:<br>G1:<br>T0-T1: P=NS

							T0-T2: P<0.05<br>G2: P=NS
						Sagittal knee motion:<br>- Flexion (°)	T0:<br>G1= 59.9 vs G2= 62.5 (P=NS)<br><br>T1:<br>G1= 62.7 vs G2= 66.2 (P=NS)<br><br>T2:<br>G1= 68.4 vs G2= 69.4 (P=NS)<br><br>Within group changes:<br>G1:<br>T0-T1: P=NS<br>T0-T2: P<0.05<br>G2:<br>T0-T1: P=NS<br>T0-T2: P<0.05
						- Extension (°)	T0:<br>G1= -10.2 vs G2= -9.2 (P=NS)<br><br>T1:<br>G1= -13.9 vs G2= -12.5 (P=NS)<br><br>T2:<br>G1= -11.5 vs G2= -10 (P=NS)<br><br>Within group changes:<br>G1: P=NS<br>G2: P=NS
						- full ROM (°)	T0:<br>G1= 49.7 vs G2= 53.3 (P=NS)<br><br>T1:<br>G1= 48.8 vs G2= 53.8 (P=NS)<br><br>T2:<br>G1= 56.9 vs G2= 59.4 (P=NS)<br><br>Within group changes:<br>G1:<br>T0-T1: P=NS

							T0-T2: P<0.05<br>G2:<br>T0-T1: P=NS<br>T0-T2: P<0.05
						Temporospatial gait parameters:<br>- Single support time (%)	T0:<br>G1= 28.6 vs G2= 32.8 (P=NS)<br><br>T1:<br>G1= 28.1 vs G2= 30.3 (P=NS)<br><br>T2:<br>G1= 35.6 vs G2= 33.9 (P=NS)<br><br>Within group changes:<br>G1:<br>T0-T1: P=NS<br>T0-T2: P<0.05<br>G2:<br>T0-T1: P=NS<br>T0-T2: P=NS
						- Double support time (%)	T0:<br>G1= 38.3 vs G2= 33.3 (P=NS)<br><br>T1:<br>G1= 40.6 vs G2= 38.6 (P=NS)<br><br>T2:<br>G1= 29.6 vs G2= 30.4 (P=NS)<br><br>Within group changes:<br>G1:<br>T0-T1: P=NS<br>T0-T2: P<0.05<br>G2:<br>T0-T1: P=NS<br>T0-T2: P<0.05
						- Cadence (steps/min)	T0:<br>G1= 100.5 vs G2= 106 (P=NS)<br><br>T1:<br>G1= 92.5 vs G2= 94.9 (P=NS)

						 <br><br><br><br><br><br>- Velocity (cm/s)<br><br><br><br><br><br><br><br><br><br><br><br><br><br><br><br><br>- Stride length (cm)<br><br><br><br><br><br><br><br><br><br><br><br><br><br><br><br><br>- anterior/posterior variability	T2:<br>G1= 108.1 vs G2= 112.5 (P=NS)<br><br>Within group changes:<br>G1: P=NS<br>G2: P=NS<br><br>T0:<br>G1= 2.5 vs G2= 2.9 (P=NS)<br><br>T1:<br>G1= 2.1 vs G2= 2.5 (P=NS)<br><br>T2:<br>G1= 3.2 vs G2= 3.3 (P=NS)<br><br>Within group changes:<br>G1:<br>T0-T1: P=NS<br>T0-T2: P=NS<br>G2:<br>T0-T1: P=NS<br>T0-T2: P<0.05<br><br>T0:<br>G1= 80.6 vs G2= 89.5 (P=NS)<br><br>T1:<br>G1= 74.3 vs G2= 85 (P=NS)<br><br>T2:<br>G1= 100.1 vs G2= 98.3 (P=NS)<br><br>Within group changes:<br>G1:<br>T0-T1: P=NS<br>T0-T2: P<0.05<br>G2:<br>T0-T1: P=NS<br>T0-T2: P=NS<br><br>T0:<br>G1= 10.3 vs G2= 7.4 (P=NS)

							T1:<br>G1= 11 vs G2= 9 (P=NS)<br><br>T2:<br>G1= 3.9 vs G2= 4.6 (P=NS)<br><br>Within group changes:<br>G1: P=NS<br>G2: P=NS
						- lateral variability	T0:<br>G1= 14.4 vs G2= 10.5 (P=NS)<br><br>T1:<br>G1= 16.8 vs G2= 11.9 (P=NS)<br><br>T2:<br>G1= 6.6 vs G2= 5.8 (P=NS)<br><br>Within group changes:<br>G1:<br>T0-T1: P=NS<br>T0-T2: P<0.05<br>G2:<br>T0-T1: P=NS<br>T0-T2: P=NS
Koseki et al. (2021) [42]	21 (NA)	Honda Walking Assist; assist hip flexion and extension	Hospital<br><br>Quasi-experimental design;<br><br>T0: Pre- TKA measurement<br><br>T1: Week 2 following TKA<br><br>T2: Week 4 following TKA<br><br>T3: Week 8 following TKA	HWA group (G1);	Conventional rehabilitation + HWA gait training from week 1 to 5 after TKA<br><br>Total of 17-20 gait training sessions (each around 20-min), with 4-5 sessions per week	Self-selected walking speed (m/s)	T0:<br>G1= 1.04 (±0.22) vs G2= 1.09 (±0.20) (P=0.586, d= 0.24)<br><br>T1:<br>G1= 0.96 (±0.17) vs G2= 0.70 (±0.29) (P=0.022, d=1.09)<br><br>T2:<br>G1= 1.13 (±0.25) vs G2= 1.00 (±0.26) (P=0.260, d=0.51)<br><br>T3:<br>G1= 1.19 (±0.23) vs G2= 1.04 (±0.19) (P=0.107, d=0.71)<br><br>T0:
				Control group (G2);	Conventional rehabilitation		

						Step length at self-selected walking speed (m)	G1= 0.55 (±0.07) vs G2= 0.58 (±0.09) (P=0.443, d= 0.37)<br><br>T1:<br>G1= 0.55 (±0.08) vs G2= 0.46 (±0.11) (P=0.032, d=0.94)<br><br>T2:<br>G1= 0.58 (±0.07) vs G2= 0.54 (±0.08) (P=0.194, d=0.53)<br><br>T3:<br>G1= 0.60 (±0.08) vs G2= 0.58 (±0.09) (P=0.852, d=0.24)
						Cadence at self-selected walking speed (steps/min)	T0:<br>G1= 112.1 (±14.05) vs G2= 112.34 (±9.61) (P=0.964, d= 0.02)<br><br>T1:<br>G1= 104.10 (±8.47) vs G2= 87.53 (±23.99) (P=0.051, d=0.92)<br><br>T2:<br>G1= 117.72 (±14.45) vs G2= 111.87 (±20.39) (P=0.446, d=0.33)<br><br>T3:<br>G1= 119.10 (±13.82) vs G2= 113.50 (±8.45) (P=0.265, d=0.49)
Yoshikawa et al. (2018) [57]	19 (NA)	HAL; Gait	Hospital<br><br>Quasi-experimental design;<br><br>T0: Pre- TKA measurement<br><br>T1: Week 1 following TKA<br><br>T2: Week 2 following TKA<br><br>T3: Week 3 following TKA<br><br>T4: Week 4 following TKA<br><br>T5: Week 8 following TKA	HAL group (G1);<br><br>Control group (G2);	10-12 HAL training sessions (around 15-min each) over a 4 week period + conventional physical therapy (60-80 min a day)<br><br>Conventional physical therapy (60-120 min a day)	Self-selected walking speed (m/s)	T0:<br>G1= 1.84 (±0.92) vs G2= 1.49 (±0.60) (P=0.794)<br><br>T2:<br>G1= 0.87 (±0.19) vs G2= 0.77 (±0.27) (P=0.366)<br><br>T4:<br>G1= 1.20 (±0.09) vs G2= 0.99 (±0.26) (P=0.030)<br><br>T5:<br>G1= 1.34 (±0.11) vs G2= 1.05 (±0.23) (P=0.022)

						Step length at self-selected walking speed (m)<br><br><br><br><br><br><br><br><br><br><br><br><br><br><br>Cadence at self-selected walking speed (steps/min)	T0:<br>G1= 0.59 (±0.11) vs G2= 0.57 (±0.11) (P=0.656)<br><br>T2:<br>G1= 0.54 (±0.05) vs G2= 0.50 (±0.09) (P=0.215)<br><br>T4:<br>G1= 0.63 (±0.03) vs G2= 0.53 (±0.07) (P=0.002)<br><br>T5:<br>G1= 0.67 (±0.03) vs G2= 0.56 (±0.08) (P=0.011)<br><br>T0:<br>G1= 108.1 (±13.7) vs G2= 109.9 (±11.1)<br><br>T2:<br>G1= 96.2 (±13.7) vs G2= 91.5 (±22.9) (P=0.051, d=0.92)<br><br>T4:<br>G1= 115.1 (±6.9) vs G2= 111.3 (±20.7)<br><br>T5:<br>G1= 119.9 (±5.3) vs G2= 113.1 (±13.4)
**Stroke:**							
Calabrò et al.. (2018) [59]	40 (18 in EGT and 14 in OGT group); mean 10 $\pm$3 m in the EGT and 11 $\pm$3 m in the OGT	Ekso; gait (hip and knee support)	Rehabiliation center<br><br>RCT<br><br>T0: Pre- gait training<br><br>T1: Post- gait training (8 weeks)	EGT (G1);	Conventional physiotherapy training + 45-min session of Ekso™ training + TMS<br><br>five 60-min sessions/ week for 8 consecutive weeks	Walking speed during 10MWT (m/s)<br><br>Hip and knee muscle activation (sEMG)<br><br>Gait quality index<br><br>Step cadence	EGT-induced improvements:<br><br>ES=0.9, P<0.001<br><br>ES=0.8, P=0.001<br><br>ES=0.9, P<0.001<br><br>ES=0.9, P<0.001<br><br>ES=0.8, P=0.008
				OGT (G2);	Conventional physiotherapy training + 45-min of conventional gait training +TMS<br><br>five 60-min sessions/ week for 8 consecutive weeks		

						Stance/swing ratio affected limb<br><br>Gait cycle duration affected limb<br><br>Gait cycle duration unaffected limb<br><br>Stance/swing ratio unaffected limb	EGT-induced reductions:<br><br>ES=0.9, P<0.001<br><br>ES=0.9, P<0.001<br><br>ES=0.9, P<0.001
Longatelli et al. (2021) [46]	29 (NA); 34.50 days [28–51] for the CG and 40 [30–64] days for the EG	Ekso GTA™; gait (hip and knee support)	Rehabilitation center<br><br>Pre-Post design<br><br>T0: Pre-intervention assessment<br><br>T1: Post-intervention assessment	CG (G1);	Standard therapy sessions<br><br>4 weeks (3 60-min sessions/week)	Gait performance indexes (include Gait Metric, Burst Duration Similarity Index and agonist-antagonist coherence function) (sEMG)	Only in the intervention group a selective improvement of muscular activation strategies (especially in the semitendinosus muscle) towards the normative patterns was found
				EG (15);	Standard therapy sessions + exoskeleton-assisted gait rehabilitation<br><br>4 weeks (12 sessions of 60 min of assisted rehabilitation 3 times/week + 8 sessions of conventional therapy)		
Rojek et al. (2020) [63]	44 (NA); 4–12 months for the experimental group and 5-12 months in the control group	Ekso; gait	Rehabilitation center<br><br>RCT;<br><br>T0: pre-intervention<br><br>T1: post-intervention (4 weeks)	Experimental group (G1) (N=23) ;	Gait training with the Ekso GT exoskeleton, occupational therapy and individually selected physical therapy<br><br>5 times a week for 4 weeks	Walking time during EksoGT gait therapy (min)<br><br>Number of steps during EksoGT gait therapy	Increase in G1:<br>Baseline- week 2 (P=0.0006)<br>Baseline-week 3 (P=0.0004)<br>Baseline-week 4 (P=0.0001)<br><br>Increase in G1:<br>Baseline- week 2 (P=0.0002)<br>Baseline-week 3 (P=0.0001)<br>Baseline-week 4 (P=0.0001)
				Control group (G2) (N=21);	Classic rehabilitation: Individual exercises with a therapist, verticalization and gait, group exercises improving general fitness, occupational therapy and individually selected elements of physical therapy<br><br>5 times a week for 4 weeks		
Taki et al. (2020) [54]	72 post-matched	HAL; gait	Hospital<br>Observational study	CRP (G1);	Gait training with KAFO or AFO	Br-stage<br>- Upper limb	T0: G1= 2 (2-4) vs G2= 2 (2-3) (Median

	study patients (CRP group: n = 36 and HRP group: n = 36) (NA);				3 hours/day for 7 days/week		(IQR)) (P=0.992)<br><br>T1: G1= 4 (2-5) vs G2= 4 (2-5) (Median (IQR)) (P=0.6)<br><br>T0-T1:<br>G1= increase of 1.17 (±1.32)<br>G2= increase of 1.28 (±1.3)<br>G1 vs G2 (P=0.725)
				HRP (G2);	Gait training with HAL<br><br>3 hours/day for 7 days/week	- Finger	T0: G1= 2 (2-5) vs G2= 2 (1-4) (Median (IQR)) (P=0.802)<br><br>T1: G1= 4 (2-5) vs G2= 5 (3-5) (Median (IQR)) (P=0.482)<br><br>T0-T1:<br>G1= increase of 0.97 (±1.42)<br>G2= increase of 1.31 (±1.45)<br>G1 vs G2 (P=0.331)
						- Lower limb	T0: G1= 3 (2-4) vs G2= 3 (2-4) (Median (IQR)) (P=0.954)<br><br>T1: G1= 4 (3-5) vs G2= 4 (2-5) (Median (IQR)) (P=0.303)<br><br>T0-T1:<br>G1= increase of 1.28 (±1.45)<br>G2= increase of 1.5 (±1.13)<br>G1 vs G2 (P=0.453)
Watanabe et al. (2017) [56]	24 (HAL® group: n= 12, CGT group: n= 12) (NA); 57.0±44.3 days HAL group and 48.1±33.3 days in the control group	HAL; gait	Hospital<br><br>RCT;<br><br>T0: Pre-intervention<br><br>T1: Post-intervention (4 weeks)<br><br>T2: Follow-up 8 weeks<br><br>T3: Follow-up 12 weeks	HAL (G1);	Gait training using the HAL<br><br>3 times a week with a total of 12 sessions over 4 weeks	Stride (m)	G1 x G2 (P=0.581)<br>G1: HAL group<br>T0: 0.37 (±0.16)<br>T1: 0.44 (±0.12)<br>T2: 0.44 (±0.14)<br>T3: 0.46 (±0.15)<br><br>G2: Conventional group<br>T0: 0.29 (±0.18)<br>T1: 0.34 (±0.18)<br>T2: 0.37 (±0.18)<br>T3: 0.36 (±0.16)
				CGT (G2);	Conventional gait training<br><br>3 times a week with a total of 12 sessions over 4 weeks		

						Cadence (steps/min)	G1 x G2 (P=0.844)<br>G1: HAL group<br>T0: 81.5 (±36.3)<br>T1: 108.4 (±33.2)<br>T2: 103.6 (±46.8)<br>T3: 99.3 (±40.3)<br><br>G2: Conventional group<br>T0: 75.1 (±37.1)<br>T1: 98.7 (±26.3)<br>T2: 83.4 (±16.2)<br>T3: 88.9 (±30.2)
Firouzi et al. (2022) [24]	5 (5); 115 ± 213.6 days	HWA; Hip flexion and extension	Hospital<br><br>Experimental cross-sectional study with crossover design	No groups	3 walking conditions:<br><br>- normal walking (I1): over a walkway at self-selected comfortable speed<br><br>- unassisted (I2): walking with the HWA but without any assistance<br><br>- optimal assisted (I3): walking with the HWA but with optimal assistance<br><br>3 times 5m walkway in each condition (total time: 40-60 min)	Walking speed (m/s)<br><br><br><br><br><br><br><br><br><br><br><br><br><br>Cadence (steps/min)<br><br><br><br><br><br><br><br><br><br><br><br>Paretic and non-paretic cycle time (s)	I1 vs I3:<br>4 out of 5 patients: increase (ranging from 0.057 to 0.095)<br><br>I1 vs I2:<br>Remained consistent in two patients, increased (ranging from 0.012 to 0.051) in two patients, and decreased (by up to -0.022) in one patient<br><br>I2 vs I3:<br>All patients: increase (ranging from 0.020 to 0.063)<br><br><br>I1 vs I3:<br>4 out of 5 patients: increase (ranging from 0.7 to 3.4)<br><br>I1 vs I2:<br>All patients: increase (ranging from 0.8-5.2)<br><br>I2 vs I3:<br>3 out of 5 patients: increase (ranging from 0.3-2.5)<br><br>I1 vs I3:<br>3 out of 5 patients: decrease (ranging from -0.04 to -0.01)<br>2 out of 5 patients: unchanged

							I1 vs I2:<br>All patients: minimal/absent changes (ranging from -0.07 to -0.01)<br><br>I2 vs I3:<br>All patients: minimal/absent changes (ranging from -0.03 to 0.06)
						Paretic stride length (m)	I1 vs I3:<br>4 out of 5 patients: increase (ranging from 0.055 to 0.069)<br>1 out of 5 patients: decrease (-0.008)<br><br>I1 vs I2:<br>3 out of 5 patients: increase (ranging from 0.009 to 0.029)<br><br>I2 vs I3:<br>4 out of 5 patients: increase (ranging from 0.036 to 0.059)
						Non-paretic stride length (m)	I1 vs I3:<br>4 out of 5 patients: increase (ranging from 0.053 to 0.077)<br>1 out of 5 patients: decrease (-0.020)<br><br>I1 vs I2:<br>3 out of 5 patients: increase (ranging from 0.011 to 0.019)<br><br>I2 vs I3:<br>All patients: increase (ranging from 0.046 to 0.082)
						Paretic stride velocity (m/s)	I1 vs I3:<br>4 out of 5 patients: increase (ranging from 0.060 to 0.090)<br><br>I1 vs I2:<br>4 out of 5 patients: increase (ranging from 0.005 to 0.050)

							I2 vs I3:<br>All patients: increase (ranging from 0.025 to 0.063)
						Non-paretic stride velocity (m/s)	I1 vs I3:<br>4 out of 5 patients: increase (ranging from 0.063 to 0.100)<br><br>I1 vs I2:<br>3 out of 5 patients: increase (ranging from 0.018 to 0.047)<br><br>I2 vs I3:<br>All patients: increase (ranging from 0.015 to 0.077)
						Paretic swing phase (% gait cycle)	I1 vs I3:<br>4 out of 5 patients: increase (ranging from 0.6 to 1.5%)<br><br>I1 vs I2:<br>4 out of 5 patients: decrease (ranging from -0.3 to -3.3%)<br>1 out of 5 patients: unchanged<br><br>I2 vs I3:<br>All patients: increase (ranging from 0.5 to 4.8%)
						Non-paretic swing phase (% gait cycle)	I1 vs I3:<br>4 out of 5 patients: increase (ranging from 0.7 to 1.3%)<br><br>I1 vs I2:<br>3 out of 5 patients: decrease (ranging from -0.3 to -2.7%)<br>1 out of 5 patients: increase (1%)<br>1 out of 5 patients: unchanged<br><br>I2 vs I3:<br>All patients: increase (ranging from 0.1 to 2.7%)

						Paretic stance phase (% gait cycle)	I1 vs I2:<br>4 out of 5 patients: increase (ranging from 0.4 to 3.4%)<br><br>I2 vs I3:<br>All patients: decrease (ranging from -0.5 to -4.9%)
						Non-paretic stance phase (% gait cycle)	I1 vs I2:<br>3 out of 5 patients: increase (ranging from 0.1 to 2.8%)<br><br>I2 vs I3:<br>All patients: decrease (ranging from -0.1 to -2.7%)<br><br>I1 vs I3:<br>All patients: decrease (ranging from-1.1 to -2.3%)
						Paretic double support phase (% gait cycle)	I1 vs I2:<br>3 out of 5 patients: increase (ranging from 0.7 to 5.6%)<br><br>I2 vs I3:<br>All patients: decrease (ranging from -1 to -7%)<br><br>I1 vs I3:<br>All patients: decrease (ranging from-0.7 to -1.6%)
						Non-paretic double support phase (% gait cycle)	I1 vs I2:<br>All patients: increase (ranging from 0.5 to 7.1%)<br><br>I2 vs I3:<br>All patients: decrease (ranging from -1 to -8.3%)<br><br>I1 vs I3:

						Paretic stride velocity (m/s)<br><br><br><br><br><br><br><br><br><br><br><br>Non-paretic stride velocity (m/s)	4 out of 5 patients: increase (ranging from 0.060 to 0.090)<br><br>I1 vs I2:<br>4 out of 5 patients: increase (ranging from 0.005 to 0.050)<br><br>I2 vs I3:<br>All patients: increase (ranging from 0.025 to 0.063)<br><br>I1 vs I3:<br>4 out of 5 patients: increase (ranging from 0.063 to 0.100)<br><br>I1 vs I2:<br>3 out of 5 patients: increase (ranging from 0.018 to 0.047)<br><br>I2 vs I3:<br>All patients: increase (ranging from 0.015 to 0.077)
Park et al. (2021) [51]	20 (NA); 13.20±7.20 days in the CPT-G and 7.60±4.9 days in the ICT-C group	Walkbot; Gait (hip, knee, and ankle support)	Hospital<br><br>RCT<br><br>T0: Pre-intervention<br><br>T1: Post-intervention	CPT-G (G1)	Conventional physical therapy and gait training<br><br>7 days/week, for 2 weeks. At least one 60-min session of physical therapy per day and the additional 30-min standard physical therapy session	Kinematic data<br>- Hip angle (°)<br>- Knee angle (°)<br><br>Kinetic data:<br>Active force (Newton)<br>- Hip<br><br><br>- Knee<br><br>- Ankle<br><br><br>Resistive force (Newton)<br>- Hip<br><br>- Knee<br><br>- Ankle	G2: ICT-C group<br>T0: 6.45 vs T1: 7.03 (P>0.05)<br>T0: 22.42 (±0.61) vs T1: 26.69 (±1.10) (P=0.00)<br><br>G2: ICT-C group<br>T0: 0.59 (±0.48) vs T1: 1.32 (±0.52) (P=0.03, ES=0.64)<br>T0: 0.05 (±0.04) vs T1: 1.66 (±1.95) (P=0.04, ES=0.64)<br>T0: 0.46 (±0.67) vs T1: 1.52 (±1.06) (P=0.02, ES=0.67)<br><br><br>T0: 6.18 (±0.21) vs T1: 2.08 (±0.11) (P=0.02, ES=1.00)<br>T0: 1.53 (±0.80) vs T1: 0.12 (±0.09) (P=0.001, ES=0.87)<br>T0: -0.84 (±0.21) vs T1: -0.07 (±0.53) (P=0.02, ES=0.85)
				ICT-C (G2)	Interlimb coordinated humanoid robot session with VR/AR game, combined with conventional physical therapy<br><br>7 days/week, for 2 weeks. At least one 60-min session of physical therapy per day and the additional 30-min ICT session		

						Peak passive stiffness (Nm)<br>- Hip<br><br>- Knee<br><br>- Ankle	T0: 1.53 (±0.23) vs T1: 0.72 (±0.17)<br>(P=0.00, ES=0.95)<br>T0: 1.17 (±0.11) vs T1: 0.70 (±0.15)<br>(P=0.00, ES=0.87)<br>T0: 0.67 (±0.33) vs T1: 0.40 (±0.11)<br>(P=0.04, ES=0.68)
						Clinical spasticity assessment (MAS: 0-4)<br>- Hip flexor	G1:<br>T0: 0 vs T1: 0.14 (±0.38)<br><br>G2:<br>T0: 0.31 (±0.59) vs T1: 0.13 (±0.35)<br><br>P (time effect)= 0.837<br>P (between groups)= 0.077<br>P (Time x Group)= 0.107<br>ES= 0.003
						- Hip extensor	G1:<br>T0: 0 vs T1: 0<br><br>G2:<br>T0: 0.44 (±0.62) vs T1: 0.25 (±0.46)<br><br>P (time effect)= 0.335<br>P (between groups)= 0.000<br>P (Time x Group)= 0.335<br>ES= 0.011
						- Knee flexor	G1:<br>T0: 0.14 (±0.38) vs T1: 0.36 (±0.63)<br><br>G2:<br>T0: 0.50 (±0.53) vs T1: 0.25 (±0.46)<br><br>P (time effect)= 0.698<br>P (between groups)= 0.555<br>P (Time x Group)= 0.368<br>ES= 0.004
						- Knee extensor	G1:<br>T0: 0.14 (±0.38) vs T1: 0.29 (±0.49)

							G2:<br>T0: 0.31 (±0.60) vs T1: 0.25 (±0.46)<br><br>P (time effect)= 0.678<br>P (between groups)= 0.580<br>P (Time x Group)= 0.335<br>ES= 0.005
						- Ankle dorsiflexor	G1:<br>T0: 0.29 (±0.76) vs T1: 0.31 (±0.59)<br><br>G2:<br>T0: 0.14 (±0.38) vs T1: 0<br><br>P (time effect)= 0.635<br>P (between groups)= 0.043<br>P (Time x Group)= 0.527<br>ES= 0.006
						- Ankle plantar-flexor	G1:<br>T0: 0.21 (±0.57) vs T1: 0.14 (±0.38)<br><br>G2:<br>T0: 0.37 (±0.74) vs T1: 0.13 (±0.35)<br><br>P (time effect)= 0.187<br>P (between groups)= 0.565<br>P (Time x Group)= 0.466<br>ES= 0.009
Son et al. (2021) [64]	24 (13); 11.7±6.9 y	Healbot T; Hip adduction/abduction and hip and knee extension/flexion	Hospital<br><br>RCT<br><br>T0: day 1<br>T1: day 5<br>T2: day 10	Pelvis-off (G1)	30-min gait training with the Healbot T. 10 sessions within one month<br><br>Pelvic movements in the transverse plane were not allowed during gait training	Self-selected speed (km/h)	G1 vs G2 (P=NS)<br><br>G1:<br>T0 vs T1: increase (P=0.00023)<br>T1 vs T2: increase (P=0.00015)<br>T0 vs T2: increase (P<0.00001)<br><br>G2:<br>T0 vs T1: increase (P=0.0008)<br>T1 vs T2: increase (P=NS)<br>T0 v T2: increase (P=0.00005)
				Pelvis-on (G2)	30-min gait training with the Healbot T. 10 sessions within one month<br><br>Pelvic movements in the transverse plane were provided during gait training	Average EMG (affected side)<br>- m. Rectus Femoris	G1:

							T0 vs T2 (P=NS)<br><br>G2:<br>T0 vs T2 (P=NS)
						- m. Tibialis Anterior	G1:<br>T0 vs T2 (P=NS)<br><br>G2:<br>T0 vs T2 (P=NS)
						- m. Gluteus Medius	G1:<br>T0 vs T2 (P=NS)<br><br>G2:<br>T0 vs T2: increase of 46.8% (P<0.05)
						- m. Biceps Femoris	G1:<br>T0 vs T2 (P=NS)<br><br>G2:<br>T0 vs T2: increase of 51.9% (P<0.05)
						- m. Gastrocnemius Medial	G1:<br>T0 vs T2: increase of 64.4% (P<0.05)<br><br>G2:<br>T0 vs T2: increase of 65.3% (P<0.05)
						- m. Gastrocnemius Lateral	G1:<br>T0 vs T2 (P=NS)<br><br>G2:<br>T0 vs T2: increase of 135.7% (P<0.05)
						Gait parameters:<br>Stride length (cm)	 G1:<br>T0 vs T2: increase of 16.5% (P<0.05)<br><br>G2:<br>T0 vs T2: increase of 3.3% (P<0.05)
						Cadence (steps/min)	G1:<br>T0 vs T2 (P=NS)

						Walking speed (km/h)	G2:<br>T0 vs T2: increase of 10.6% (P<0.05)<br><br>G1:<br>T0 vs T2 (P=NS)<br><br>G2:<br>T0 vs T2: increase of 11.8% (P<0.05)
Yeung et al. (2021) [65]	47 (NA); 27±17 days	Exoskeleton ankle robot; Ankle dorsiflexion and plantar flexion	Bi-center<br><br>RCT<br><br>T0: Pre-intervention<br><br>T1: Post-intervention	PAAR (G1) (N=14)	Conventional training routine (2h/weekday) + 30-min robot-assisted (Power-Assisted Ankle Robot) training (10-min stair training and two times 10-min overground walking)	Self-selected walking speed (10MWT)(m/s)	T0 (mean (±SD):<br>G1= 0.13 (±0.16)<br>G2= 0.14 (±0.16)<br>G3= 0.14 (±0.18)<br><br>T0-T1 (mean difference [95% CI]):<br>G1= increase of 0.32 [0.18, 0.46] (P<0.001)<br>G2= increase of 0.17 [0.09, 0.25] (P<0.01)<br>G3= increase of 0.17 [0.06, 0.29] (P<0.01)<br><br>Between-group differences of score improvement (T0-T1):<br>G1 vs G3= 0.15 [0.0, 0.29] (P<0.05, ES=0.641)<br>G2 vs G3= 0.0 [-0.15, 0.14] (P=NS, ES=0.020)<br>G1 vs G2= 0.15 [0.0, 0.30] (P<0.05, ES=0.752)
				SCAR (G2) (N=16)	Conventional training routine (2h/weekday) + 30-min robot-assisted (Swing-Controlled Ankle Robot) training (10-min stair training and two times 10-min overground walking)		
				CT (G3) (N=17)	Conventional training routine (2h/weekday)		
**Healthy older adults**							
Jayaraman et al. (2022) [36]	12 (12)	GEMS-H; hip flexion and extension	Senior living community<br><br>Quasi-experimental study: Pre-Post test design<br><br>T0: Pre-intervention<br><br>T1: Post-intervention	No groups	Twelve gait training sessions (30min each) over a period of 4–6 weeks	10MWT (m/s)<br>- Self-selected gait speed	T0 vs T1: improvement of 0.18 m/s (P= 0.001)
Lee et al. (2017) [43]	30 (30)	GEMS; Hip flexion and extension	Rehabilitation center	No groups	1 session	Gait speed (km/h)	I1: 3.53 (±0.54) vs I2: 3.46 (±0.54) vs I3: 4.00 (±0.13)<br>I1 vs I2 (P=NS)

			Experimental cross-sectional study: Within-subject design		15 overground walking trials at comfortable speed under three different conditions: free gait without robot Assistance (I1), RAG with zero torque (I2), and full RAG (I3).	 <br><br>Cadence (steps/min)<br><br><br><br><br><br>Stride length (cm)<br><br><br><br><br><br>Step width (cm)<br><br><br><br><br><br>Single support time (%cycle)<br><br><br><br><br><br><br>Muscle activity (sEMG)	I1 vs I3 (P<0.01)<br>I2 vs I3 (P<0.01)<br><br>I1: 105.61 (±5.46) vs I2: 103.71 (±15.04) vs I3: 111.32 (±9.43)<br>I1 vs I2 (P=NS)<br>I1 vs I3 (P<0.01)<br>I2 vs I3 (P<0.01)<br><br>I1=107.23 (±15.04) vs I2=110.37 (±15.04) vs I3=117.76 (±12.95)<br>I1 vs I2 (P=NS)<br>I1 vs I3 (P<0.01)<br>I2 vs I3 (P<0.01)<br><br>I1=15.10 (±2.11) vs I2=13.23 (±2.08) vs I3=11.68 (±3.2)<br>I1 vs I2 (P=NS)<br>I1 vs I3 (P<0.01)<br>I2 vs I3 (P<0.05)<br><br>I1=35.15 (±3.53) vs I2=36.0 (±3.50) vs I3=38.89 (±3.06)<br>I1 vs I2 (P=NS)<br>I1 vs I3 (P<0.01)<br>I2 vs I3 (P<0.01)<br><br>Reduction in muscle activation (%MVC) due to hip assistance by the GEMS
Lee et al. (2017) [44]	30 (30)	GEMS; Hip flexion and extension	Research center<br><br>Within-subject design	No groups	1 session<br><br>10-m overground walking at comfortable speed under three different conditions: free gait without robot Assistance (I1), RAG with zero torque (I2), and full RAG (I3).	Gait speed (cm/s)<br><br><br><br><br><br>Cadence (steps/min)<br><br><br><br><br><br>Stride length (cm)	I1=97.94 (±15.28) vs I2=97.98 (±15.52) vs I3=110.71 (±13.14)<br>I1 vs I2 (P=NS)<br>I1 vs I3 (P<0.05)<br>I2 vs I3 (P<0.05)<br><br>I1=107.90 (±5.80) vs I2=105.48 (±8.54) vs I3=113.36 (±6.92)<br>I1 vs I2 (P=NS)<br>I1 vs I3 (P<0.05)<br>I2 vs I3 (P<0.01)<br><br>I1=107.23 (±15.56) vs I2=108.24 (±13.57) vs I3=117.76 (±12.95)<br>I1 vs I2 (P=NS)

							I1 vs I3 (P<0.05)<br>I2 vs I3 (P<0.05)
						Step width (cm)	I1=11.64 (±3.03) vs I2=11.01 (±2.94) vs I3=11.85 (±3.96)<br>I1 vs I2 (P=NS)<br>I1 vs I3 (P=NS)<br>I2 vs I3 (P=NS)
						Single support time (%cycle)	I1=36.02 (±2.73) vs I2=36.46 (±2.81) vs I3=38.64 (±2.14)<br>I1 vs I2 (P=NS)<br>I1 vs I3 (P<0.01)<br>I2 vs I3 (P<0.05)
						Muscle activity (sEMG)	- Rectus Femoris and Medial Gastrocnemius muscle activity in I3 were reduced compared to I1 and I2 throughout the terminal stance phase (p<0.05)<br>- Medial Gastrocnemius muscle activity in I3 was reduced compared to I1 and I2 throughout pre-swing phase (p<0.05)
						Foot pressure distribution<br>Maximum Force (%BW)	
						- Total foot	I1=112.85 (±10.30) vs I2=119.56 (±12.06) vs I3=128.97 (±14.55)<br>I1 vs I2 (P=NS)<br>I1 vs I3 (P<0.01)<br>I2 vs I3 (P<0.05)
						- Medial Masks	I1=20.92 (±4.08) vs I2=21.59 (±4.33) vs I3=24.21 (±4.36)<br>I1 vs I2 (P=NS)<br>I1 vs I3 (P<0.05)<br>I2 vs I3 (P<0.05)
						- Lateral Masks	I1=13.75 (±2.29) vs I2=13.94 (±2.89) vs I3=13.83 (±3.85)<br>I1 vs I2 (P=NS)<br>I1 vs I3 (P=NS)<br>I2 vs I3 (P=NS)
						- Anterior Masks	

							I1=10.89 (±1.69) vs I2=11.37 (±1.75) vs I3=13.48 (±2.42)<br>I1 vs I2 (P=NS)<br>I1 vs I3 (P<0.01)<br>I2 vs I3 (P<0.01)
						- Posterior Masks	I1=31.66 (±6.56) vs I2=32.84 (±5.76) vs I3=37.81 (±7.96)<br>I1 vs I2 (P=NS)<br>I1 vs I3 (P<0.05)<br>I2 vs I3 (P<0.05)
						Peak Pressure (Newton/cm^2)<br>- Total foot	I1=34.63 (±11.32) vs I2=35.72 (±10.73) vs I3=48.24 (±18.49)<br>I1 vs I2 (P=NS)<br>I1 vs I3 (P<0.01)<br>I2 vs I3 (P<0.05)
						- Medial Masks	I1=22.28 (±7.52) vs I2=22.68 (±7.67) vs I3=30.69 (±12.26)<br>I1 vs I2 (P=NS)<br>I1 vs I3 (P<0.05)<br>I2 vs I3 (P<0.05)
						- Lateral Masks	I1=18.63 (±4.84) vs I2=19.52 (±6.05) vs I3=17.06 (±5.06)<br>I1 vs I2 (P=NS)<br>I1 vs I3 (P=NS)<br>I2 vs I3 (P=NS)
						- Anterior Masks	I1=16.47 (±4.14) vs I2=16.35 (±3.63) vs I3=20.01 (±5.84)<br>I1 vs I2 (P=NS)<br>I1 vs I3 (P<0.05)<br>I2 vs I3 (P<0.05)
						- Posterior Masks	I1=4.30 (±1.87) vs I2=4.65 (±1.76) vs I3=5.29 (±1.99)<br>I1 vs I2 (P=NS)<br>I1 vs I3 (P=NS)<br>I2 vs I3 (P=NS

Jin et al. (2017) [37]	9 (9)	Soft wearable robotic suit built in-house; gait with hip and knee control	Research center<br><br>Within-subject design	No groups	2 sets of measurements in 2 days<br><br>four 6-minute treadmill walking trials at comfortable speed, separated by rest periods. Each session implemented alternate conditions: robotic suit worn and powered on (PON) and off (POFF).<br>I1: Robotic suit worn ad powered off (POFF)<br>I2: Robotic suit worn and powered on (PON)	Maximum hip angle (°)<br><br>Maximum vertical displacement of COM (m)<br><br>Maximum vertical position of knee (m)<br><br>Maximum vertical position of ankle (m)<br><br>Maximum vertical position of toe (m)<br><br>Stride duration (s)<br><br>Walk ratio (m/steps/min):	I2=28.4 vs I1=26.9 (P<0.05)<br><br>I2=0.033 vs I1=0.029 (P<0.01)<br><br>I2=0.54 vs I1=0.52 (P<0.001)<br><br>I2=0.26 vs I1=0.26 (P=NS)<br><br>I2=0.15 vs I1 (0.14) (P<0.001)<br><br>I2=1.14 vs I1=1.09 (P<0.001)<br><br>I2=0.0057 vs I1=0.0052 (P<0.001)
Jin et al. (2019) [41]	4 (4)	Soft robotic suit; hip flexion	Research center<br><br>Prospective design;<br><br>T0: Baseline<br><br>T1: After 6 weeks of training	No groups	Six-week training intervention, with 4 trials of 6-min treadmill walking each day, implementing alternate conditions: robotic suit worn and powered on (PON) and off (POFF).<br><br>I1: Robotic suit worn ad powered off (POFF)<br><br>I2: Robotic suit worn and powered on (PON)	I1 vs I2:<br><br>Maximum hip angle (°)<br><br>Maximum knee angle (°)<br><br>Maximum ankle angle (°)<br><br>Walk ratio (m/steps/min)<br><br>Kinematics assessed in POFF condition:<br><br>Maximum hip flexion (°)<br><br>Maximum hip extension (°)<br><br>Maximum knee flexion (°)<br><br>Maximum knee extension (°)	 <br>average increase of 10.5% from I1 to I2<br><br>average increase of 1.9% from I1 to I2<br><br>average increase of 33.7% from I1 to I2<br><br>I1= 0.0054 vs I2= 0.0057 (P=0.0052)<br><br><br><br>T0: 27.6 (±3.1) vs T1: 22.5 (±3.2) (reduction of 20.7%, P=0.0411)<br><br>T0: 15.4 (±9.1) vs T1: 20.8 (±5.2) (P=0.1861)<br><br>T0: 66.5 (±5.0) vs T1: 58.3 (±6.2) (reduction of 12.5%, P=0.0350)<br><br>T0: 10.1 (±9.0) vs T1: 3.6 (±3.9) (P=0.0892)

						Maximum ankle dorsiflexion (°)<br><br>Maximum ankle plantarflexion (°)<br><br>Walk ratio (m/steps/min)	T0: 7.4 (±5.0) vs T1 : 3.4 (±5.8) (reduction of 54.3%, P=0.0085)<br><br>T0: 19.44 (±6.1) vs T1 : 21.4 (±5.6) (P=0.1272)<br><br>T0 : 0.0052 vs T1 : 0.0057 (increase of 9.8%, P=0.1460)
Fang et al. (2022) [33]	6 (6)	Dual-mode ankle exoskeleton; ankle plantar flexor assistance as a mobility aid and plantar flexor resistance as a functional muscle recruitment training platform.	Rehabilitation center<br><br>Within-subject design<br><br>Pilot subject:<br>T0: Pre resistance-training<br><br>T1: Post resistance-training	No groups	2 visits assessing 2 different protocols<br><br>Assistance protocol: treadmill waking in two conditions: exo-adaptation (I1) (30-min of bilateral plantarflexion and dorsiflexion assistance) and shod (I2) (no exoskeleton).<br><br>Resistance protocol: treadmill waking in two conditions: baseline (I3) (without exoskeleton) and resisted (I4) (bilateral plantar flexor resistance and biofeedback).<br><br>Resistance training protocol: One pilot participant performed 12 sessions of resistance training over four weeks (T0: Pre resistance-training, T1: Post resistance-training)	*Ankle assistance protocol:*<br><br>Minimum soleus variance ratio<br><br>Lowest soleus iEMG<br><br>*Ankle resistance protocol:*<br><br>Stance-phase soleus iEMG<br><br>Stance-phase average positive ankle power<br><br>*Resistance training protocol (N=1)*<br><br>Self-selected speed (m/s)	 <br>I1 is 30.3% (±14.1) greater than I2 (P=0.027)<br><br>I1 is 17.0% (±10.2) lower than I2 (P=0.009)<br><br><br><br>I4: 99.5% (±57.2) increase compared to I3 (P=0.007)<br><br>I4: 55.4% (±28.3) greater compared to I3 (P=0.013)<br><br><br><br>T0: 1.07 vs T1: 1.12
Galle et al. (2022) [35]	8 (8)	Bilateral ankle-foot exoskeletons ; plantar flexion during push-off	Hospital<br><br>Within-subject design	No groups	four walking trials (five minutes with three minutes rest in between) in four different conditions:<br><br>I1: Waking with normal shoes<br>I2: Walking with exoskeleton powered off<br>I3: Walking with exoskeleton powered on in first condition	Perceived balance (VAS)<br><br>Step length (m)	I1 vs I3 (P=NS)<br>I1 vs I4 (P=NS)<br><br>I1= 0.59 (±0.04), I2: 0.59 (±0.03), I3: 0.61 (±0.05) and I4: 0.62 (±0.05)<br><br>I1 vs I2 (P=NS)<br>I1 vs I3 (P=NS)<br>I1 vs I4 (P=0.02, ES=0.67)<br>I2 vs I3 (P=0.04, ES=0.38)

					I4: Walking with exoskeleton powered on in second condition	Step width (m)	I2 vs I4 (P=0.01, ES=0.73)<br><br>I1= 0.10 (±0.02), I2: 0.10 (±0.01), I3: 0.10 (±0.02) and I4: 0.09 (±0.01)<br><br>I1 vs I2 (P=NS)<br>I1 vs I3 (P=NS)<br>I1 vs I4 (P=NS)<br>I2 vs I3 (P=NS)<br>I2 vs I4 (P=NS)
Lee et al. (2022) [62]	60 (60)	EX1; hip flexion and extension	Rehabilitation center<br><br>Randomized controlled trial<br><br>T0: Pre intervention<br>T1: Post intervention	Group A (G1)	4 weeks of exercises with 3 sessions per week (40min each). Exercise consists of overground walking without exoskeleton at a comfortable speed for 300m straight.	10MWT: self-selected velocity (m/s)	T0-T1 :<br>G1 : 0.04 (±0.17) (P=NS)<br>G2 : 0.09 (±0.13) (P<0.05)<br>G3 : 0.10 (±0.15) (P<0.05)<br>G4 : 0.10 (±0.15) (P<0.05)
				Group B (G2)	4 weeks of exercises with 3 sessions per week (40min each). Exercise consists of overground walking using the resistance mode of the exoskeleton at a comfortable speed for 300m straight.		
				Group C (G3)	4 weeks of exercises with 3 sessions per week (40min each). Exercise consists of stair ascent using the assistance mode of the exoskeleton at a comfortable speed from the first basement level to the fourth floor.		
				Group D (G4)	4 weeks of exercises with 3 sessions per week (40min each). Exercise consists of incline treadmill walking using the assistance mode of the exoskeleton at a comfortable speed.		
**Vitality performance indicator**							
**Neurological diseases**							
Panizzolo et al. (2022) [50]	10 (NA)	Exoband; passive hip assistance	Rehabilitation center	No groups	10 walking sessions (10-min each) wearing the Exoband, for 5 consecutive weeks	RPE difference between sessions 1 and 10 (Borg CR-10)	T0: 5.1 (±2.7) (95% CI: 3.2 – 7.0)<br>T1: 4.8 (±2.8) (95% CI: 2.8 – 6.8)<br>T0-T1: P>0.05, ES: 0.11

			Quasi-experimental: Pre-Post test design<br><br>T0: session 1 of wearing the exoband<br><br>T1: session 10 of wearing the exoband			Correlation between sessions spent walking with Exoband and RPE	R= -0.813; P<0.01; 95% CI: -0.95 – -0.38
**Osteoarthritis**							
Setoguchi et al. (2022) [53]	47 (NA)	HAL;	Hospital<br><br>RCT<br><br>T0: Preoperatively<br><br>T1: 1 week postoperatively<br><br>T2: 3 weeks postoperatively	HAL group (G1);	HAL gait training: 3 sessions per week (40-min each) for 6 weeks in total + typical physical therapy on days without HAL training	SF-36 - Vitality	T0:<br>G1= 48.6 (±7.06) vs G2= 41.8 (±10.99) (P=0.218)<br><br>T2:<br>G1= 53.0 (±9.24) vs G2= 52.2 (±6.59) (P=0.516)<br><br>Within group changes:<br>G1:<br>T0-T2: P=NS<br>G2:<br>T0-T2: P<0.05
				Control group (G2);	Typical physical therapy (40-min) each day		
**Stroke**							
Lefeber (2018) [45]	12 (NA); median 80 days	Lokomat; gait	Within-subject design;<br><br>T1: minute 6<br><br>T2: minute 12<br><br>T3: minute 18	No groups	1st session: walking in a treadmill-based exoskeleton according to conventional training parameters (I1)<br><br>2nd and 3rd session: walking with two different levels of robotic assistance: one session 100% (I2) and one session 60% (I1) (randomized)<br><br>3 RATW sessions (20 min each) (1 session/day)	Net RPE (0-14 Borg scale)	Values = median (IQR)<br><br>I1:<br>T1= 2.0 (4.0), T2= 3.5 (3.0) and T3= 5 (4.0)<br>T1 vs T2 (P=0.005)<br>T1 vs T3 (P=0.007)<br><br>I2:<br>T1= 2.0 (1.0), T2= 2.5 (2.0) and T3= 2.5 (3.0)<br><br>I3:<br>T1= 2.0 (3.0), T2= 2.0 (2.0) and T3= 3.5 (3.0)<br><br>T1, T2 & T3 : I2 vs I3 (P=NS)
						Net oxygen consumption (mL/kg/min)	I1:

							T1= 2.49 (1.35), T2= 2.27 (1.86) and T3= 2.43 (1.88)<br>T1 vs T2 (P>0.05)<br>T1 vs T3 (P>0.05)<br><br>I2:<br>T1= 2.71 (0.99), T2= 2.71 (1.11) and T3= 2.88 (1.09)<br><br>I3:<br>T1= 1.96 (1.07), T2= 2.33 (1.38) and T3= 2.35 (1.49)<br><br>T1: I2 vs I3 (P=0.037)<br>T2 & T3: I2 vs I3 (P=NS)
						Net carbon dioxide production (mL/kg/min)c	I1:<br>T1= 1.64 (1.24), T2= 1.86 (1.31) and T3= 2.00 (1.86)<br>T1 vs T2 (P>0.05)<br>T1 vs T3 (P>0.05)<br><br>I2:<br>T1= 2.12 (0.70), T2= 2.21 (0.94) and T3= 2.35 (1.04)<br><br>I3:<br>T1= 1.63 (0.88), T2= 1.72 (1.24) and T3= 1.97 (1.35)<br><br>T1, T2 & T3 : I2 vs I3 (P=NS)
						Net respiratory exchange ratio (no unit)	I1:<br>T1= -0.02 (0.06), T2= 0.01 (0.07) and T3= 0.01 (0.07)<br>T1 vs T2 (P>0.05)<br>T1 vs T3 (P>0.05)<br><br>I2:<br>T1= -0.01 (0.04), T2= 0.01 (0.02) and T3= 0.00 (0.03)<br><br>I3:<br>T1= 0.02 (0.07), T2= 0.00 (0.05) and T3= 0.02 (0.05)

							T1: I2 vs I3 (P=0.047)<br>T2 & T3: I2 vs I3 (P=NS)
						Net oxygen cost (mL/kg/m)	I1:<br>T1= 0.07 (0.05), T2= 0.06 (0.04) and T3= 0.07 (0.06)<br>T1 vs T2 (P>0.05)<br>T1 vs T3 (P>0.05)<br><br>I2:<br>T1= 0.07 (0.03), T2= 0.07 (0.03) and T3= 0.07 (0.03)<br><br>I3:<br>T1= 0.05 (0.03), T2= 0.06 (0.04) and T3= 0.06 (0.04)<br><br>T1: I2 vs I3 (P=0.037)<br>T2 & T3: I2 vs I3 (P=NS)
						Net heart rate (beats/min)	I1:<br>T1= 8 (16), T2= 10 (14) and T3= 10 (17)<br>T1 vs T2 (P>0.05)<br>T1 vs T3 (P>0.05)<br><br>I2:<br>T1= 9 (9), T2= 9 (9) and T3= 10 (8)<br><br>I3:<br>T1= 6 (4), T2= 7 (6) and T3= 8 (6)<br><br>T1, T2 & T3 : I2 vs I3 (P=NS)
						Net Minute ventilation (L/min)	I1:<br>T1= 5.18 (4.72), T2= 5.01 (2.21) and T3= 5.52 (3.84)<br>T1 vs T2 (P>0.05)<br>T1 vs T3 (P>0.05)<br><br>I2:<br>T1= 6.67 (2.13), T2= 7.09 (2.58) and T3= 7.31 (3.35)

						Net breathing frequency (breaths/min)	I3:<br>T1= 5.46 (1.87), T2= 5.53 (2.21) and T3= 6.99 (3.62)<br><br>T1, T2 & T3 : I2 vs I3 (P=NS)<br><br>I1:<br>T1= 6 (6), T2= 6 (7) and T3= 6 (8)<br>T1 vs T2 (P>0.05)<br>T1 vs T3 (P>0.05)<br><br>I2:<br>T1= 7 (3), T2= 7 (4) and T3= 7 (4)<br><br>I3:<br>T1= 7 (4), T2= 6 (3) and T3= 6 (4)<br><br>T1, T2 & T3 : I2 vs I3 (P=NS)
**Sarcopenia**							
Norris et al. (2006) [49]	7 (7)	PAFO; ankle plantarflexion assistance	Cross-sectional design;	Older adults	Participants walked across 3 conditions at their preferred walking speed:<br>I1= with standard shoes,<br>I2= with the PAFOs inactive,<br>I3= with the PAFOs active	Metabolic cost of transport (W/N/(m/s))<br><br>Metabolic energy per stride (J/kg)<br><br>(at the fixed preferred walking speed)	I1= 0.399 (±0.048), I2= 0.406 (±0.054) and I3= 0.389 (±0.044)<br><br>I1= 5.103 (±0.601), I2= 4.862 (±0.362) and I3= 4.812 (±0.562)
				Young adults (data not considered)	Participants walked across 3 conditions at their preferred walking speed:<br>I1= with standard shoes,<br>I2= with the PAFOs inactive,<br>I3= with the PAFOs active		
**Healthy older adults**							
Lee et al. (2017) [43]	30 (30)	GEMS; Hip flexion and extension	Rehabilitation center<br><br>Experimental cross-sectional study: Within-subject design	No groups	1 session<br><br>15 overground walking trials at comfortable speed under three different conditions: free gait without robot Assistance (I1), RAG with zero torque (I2), and full RAG (I3).	Oxygen consumption per unit mass<br><br>EEm (kcal/min)<br><br>(at own comfortable speed)	about 7% lower in I3 vs I1 at self-selected speed (P<0.05)<br><br>6.6% lower in I3 vs I1 at self-selected speed (P<0.05)

Jin (2017) [37]	9 (9)	Soft wearable robotic suit built in-house; gait with hip and knee control	Within-subject design	No groups	2 sets of measurements in 2 days<br><br>four 6-minute treadmill walking trials at comfortable speed, separated by rest periods. Each session implemented alternate conditions: robotic suit worn and powered on (PON) and off (POFF).<br>I1: Robotic suit worn ad powered off (POFF)<br>I2: Robotic suit worn and powered on (PON)	Energy expenditure (W/kg)<br><br>Energy efficiency<br><br>(at fixed preferred walking speed)	I2=3.67 vs I1=3.90 (5.9% difference; P<0.05)<br><br>With the average 3.52 W injected power the average efficiency of the robotic suit reaches 75.1% at maximum
Fang et al. (2022) [33]	6 (6)	Dual-mode ankle exoskeleton; ankle plantar flexor assistance as a mobility aid and plantar flexor resistance as a functional muscle recruitment training platform.	Within-subject design<br><br>Pilot subject:<br>T0: Pre resistance-training<br><br>T1: Post resistance-training	No groups	2 visits assessing 2 different protocols<br><br>Assistance protocol: treadmill waking in two conditions: exo-adaptation (I1) (30-min of bilateral plantarflexion and dorsiflexion assistance) and shod (I2) (no exoskeleton).<br><br>Resistance protocol: treadmill waking in two conditions: baseline (I3) (without exoskeleton) and resisted (I4) (bilateral plantar flexor resistance and biofeedback).<br><br>Resistance training protocol: One pilot participant performed 12 sessions of resistance training over four weeks (T0: Pre resistance-training, T1: Post resistance-training)	*Ankle assistance protocol:*<br><br>Net metabolic power<br><br>*Resistance training protocol (N=1)*<br><br>Metabolic power	No group level difference between I1 and I2 (P=0.317)<br>,yet four out of five participants experienced a reduction (up to 19%) in metabolic power during assisted walking compared to their baseline.<br>Notably, participants with higher baseline metabolic power demonstrated more substantial reductions while walking with assistance ($R2=0.898$, P=0.014).<br><br>T0 vs T1: decrease of 9%

Galle et al. (2022) [35]	8 (8)	Bilateral ankle-foot exoskeletons ; plantar flexion during push-off	Within-subject design	No groups	four walking trials (five minutes with three minutes rest in between at a fixed speed) in four different conditions:<br><br>I1: Waking with normal shoes<br>I2: Walking with exoskeleton powered off<br>I3: Walking with exoskeleton powered on in first condition<br>I4: Walking with exoskeleton powered on in second condition	Net metabolic power (W/kg)<br><br>Perceived fatigue in the legs (VAS)<br><br>(at fixed speed)	I1= 3.24 (±0.36), I2: 3.55 (±0.38), I3: 3.23 (±0.49) and I4: 3.12 (±0.40)<br><br>I1 vs I2 (P=0.02, ES=0.84)<br>I1 vs I3 (P=0.90, ES=0.04)<br>I1 vs I4 (P=0.23, ES=0.31)<br>I2 vs I3 (P=0.01, ES=0.75)<br>I2 vs I4 (P=0.01, ES=1.10)<br><br>I1 vs I3 (P=NS)<br>I1 > I4 (P=0.05, ES=0.91)
Martini et al. (2019) [47]	20 (20)	APO; Hip flexion and extension	RCT<br><br>T0: baseline measurement<br><br>T1: post-training program (end of week 4)<br><br>T2: follow-up measurement (one-month)	APO group (G1)	four-week robot-assisted gait training program, followed by a one-month follow-up period.<br><br>+<br><br>Comparison between walking with APO (I1) and free walking (I2) to assess training efficiency	Metabolic Cost of Transport (ml/kg/m)<br><br>Robot-assisted training efficiency (G1)<br><br>Oxygen uptake rate (ml/min/kg)<br><br>Metabolic power (W/kg)	T0:<br>G1=0.162 (±0.038) vs G2= 0.145 (±0.038) (P=0.33)<br><br>T1:<br>G1= 0.115 (±0.021) vs G2: 0.133 (±0.020)<br><br>T2:<br>G1= 0.124 (±0.024) vs G2: 0.137 (±0.024)<br><br>T0-T1:<br>G1: reduction of 0.115 (±0.021) (26.6% ±16.1) (P<0.01)<br>G2: reduction of 0.113 (±0.020) (P=0.44)<br><br>T0-T2:<br>G1: reduction of 0.124 (±0.024) (20.2% ±19.7) (P<0.01)<br>G2: reduction of 0.137 (±0.024) (P=0.44)<br><br>I1= 10.06 (±1.88) vs I2= 10.54 (±2.11) (P=0.0024)<br><br>I1= 3.45 (±0.64) vs I2= 3.58 (±0.72) (P=0.011)
				Control group (G2)	four-week home exercise program of self-paced overground walking, followed by a one-month follow-up period.		

						(at fixed speed)	
Lee et al. (2022) [62]	60 (60)	EX1; hip flexion and extension	Randomized controlled trial<br><br>T0: Pre intervention<br>T1: Post intervention	Group A (G1)	4 weeks of exercises with 3 sessions per week (40min each). Exercise consists of overground walking without exoskeleton at a comfortable speed for 300m straight.	Net cardiopulmonary metabolic cost (mL.kg^{-1}.min^{-1})	T0-T1:<br>G1: 4.96% reduction (P=NS)<br>G2: 8.28% reduction (P=NS)<br>G3: 12.80% reduction (P<0.05)<br>G4: 21.66% reduction (P<0.05)
				Group B (G2)	4 weeks of exercises with 3 sessions per week (40min each). Exercise consists of overground walking using the resistance mode of the exoskeleton at a comfortable speed for 300m straight.	EEm (kcal/min)<br><br>(at fixed comfortable speed)	G1: 4.39% reduction (P=NS)<br>G2: 7.88% reduction (P=NS)<br>G3: 10.03% reduction (P<0.05)<br>G4: 18.30% reduction (P<0.05)
				Group C (G3)	4 weeks of exercises with 3 sessions per week (40min each). Exercise consists of stair ascent using the assistance mode of the exoskeleton at a comfortable speed from the first basement level to the fourth floor.		
				Group D (G4)	4 weeks of exercises with 3 sessions per week (40min each). Exercise consists of incline treadmill walking using the assistance mode of the exoskeleton at a comfortable speed.		
**Psychological performance indicators**							
**Parkinson's Disease**							
Gryfe et al. (2022) [60]	40 (NA)	Keeogo Rehab™; powered knee assistance	Hospital<br><br>RCT<br><br>T0: Baseline measurement	Exoskeleton exercise (Exo);<br><br>G1	8 weeks of aerobic, strength and functional mobility exercises with the exoskeleton (16 sessions, 2 times a week)	UPDRS - Mentation sub-scale (max 16)	G1:<br>T0: 3.6 (±1.5)<br>T1: 3.6 (±1.9)<br>T0-T1: change of 0.0 (±1.2) (P=1.000, P significance level = 0.017))

			T1: Post- intervention (8 weeks)				
				Non-exoskeleton exercise (Nxo);<br><br>G2	8 weeks of aerobic, strength and functional mobility exercises without the exoskeleton (16 sessions, 2 times a week)		G2:<br>T0: 3.5 (±2.0)<br>T1: 3.4 (±1.9)<br>T0-T1: decrease of 0.1 (±1.5) (P=0.720, P significance level = 0.017))<br><br>G3:<br>T0: 4.2 (±1.7)<br>T1: 4.0 (±2.2)<br>T0-T1: decrease of 0.2 (±1.3) (P=0.534, P significance level = 0.017))<br><br>Between-groups comparison:<br>T0: (P=0.518)<br>T0-T1: (P=0.945)
				Wait-list control (Con);<br><br>G3	No intervention	PDQ-39 - Emotional well-being sub-scale (max 100)	G1:<br>T0: 20.5 (±12.2)<br>T1: 16.3 (±9.8)<br>T0-T1: decrease of 4.2 (±4.8) (P=0.009, P significance level = 0.017))<br><br>G2:<br>T0: 21.8 (±18.0)<br>T1: 22.4 (±19.7)<br>T0-T1: increase of 0.6 (±11.4) (P=0.843, P significance level = 0.017))<br><br>G3:<br>T0: 22.8 (±15.7)<br>T1: 21.8 (±14.7)<br>T0-T1: decrease of 1.0 (±11.9) (P=0.776, P significance level = 0.017))<br><br>Between-groups comparison:<br>T0: (P=0.934)<br>T0-T1: (P=0.377)
						PDQ-39 - Stigma sub-scale (max 100)	G1:<br>T0: 20.2 (±15.8)<br>T1: 14.9 (±10.1)<br>T0-T1: decrease of 5.3 (±11.1) (P=0.111, P significance level = 0.017))

							G2:<br>T0: 25.0 (±23.9)<br>T1: 22.6 (±25.1)<br>T0-T1: decrease of 2.4 (±13.4) (P=0.530, P significance level = 0.017))<br><br>G3:<br>T0: 14.9 (±17.6)<br>T1: 11.5 (±13.9)<br>T0-T1: decrease of 3.4 (±10.1) (P=0.252, P significance level = 0.017))<br><br>Between-groups comparison:<br>T0: (P=0.424)<br>T0-T1: (P=0.555)
						HADS- Anxiety Score (max 21)	G1:<br>T0: 4.4 (±2.6)<br>T1: 4.2 (±2.5)<br>T0-T1: decrease of 0.2 (±2.0) (P=0.470, P significance level = 0.017))<br><br>G2:<br>T0: 5.7 (±2.5)<br>T1: 5.3 (±3.8)<br>T0-T1: decrease of 0.4 (±3.3) (P=0.158, P significance level = 0.017))<br><br>G3:<br>T0: 5.5 (±3.1)<br>T1: 4.8 (±2.6)<br>T0-T1: decrease of 0.7 (±1.8) (P=0.356, P significance level = 0.017))<br><br>Between-groups comparison:<br>T0: (P=0.407)<br>T0-T1: (P=0.959)
						HADS- Depression Score (max 21)	G1:<br>T0: 5.8 (±3.5)<br>T1: 5.3 (±3.3)<br>T0-T1: decrease of 0.5 (±2.6) (P=0.790, P significance level = 0.017))<br><br>G2:

							T0: 7.3 (±4.8)<br>T1: 6.1 (±4.1)<br>T0-T1: decrease of 1.1 (±2.9) (P=0.637, P significance level = 0.017))<br><br>G3:<br>T0: 5.2 (±3.7)<br>T1: 4.8 (±3.4)<br>T0-T1: decrease of 0.4 (±1.4) (P=0.190, P significance level = 0.017))<br><br>Between-groups comparison:<br>T0: (P=0.402)<br>T0-T1: (P=0.943)
Neurological diseases							
Roggeman et al. (2022) [52]	11 (11)	Honda Walking Assist; powered hip assistance	Cross-sectional study	Older adults	30-min of walking with the HWA	Intrinsic Motivation Inventory:<br><br>- interest/enjoyment (max 49)	Values = medians (25th- 75th percentile)<br><br>43 (41-47)
				Rehabilitation patients (data not considered)	30-min of walking with the HWA	- perceived competence (max 42)<br><br>- effort/importance (max 35)<br><br>- pressure/tension (max 35)<br><br>- value/usefulness (max 49)<br><br>- relatedness (max 35)	36 (33-40)<br><br>19 (17-29)<br><br>6 (6-8)<br><br>45 (35-49)<br><br>32 (29-34)
Osteoarthritis							
Setoguchi et al. (2022) [53]	47 (NA)	HAL;	RCT<br><br>T0: Preoperatively<br><br>T1: 1 week postoperatively<br><br>T2: 3 weeks postoperatively	HAL group (G1);	HAL gait training: 3 sessions per week (40-min each) for 6 weeks in total + typical physical therapy on days without HAL training	SF-36 - Role emotional	T0:<br>G1= 31.1 (±8.90) vs G2= 34.2 (±17.49) (P=0.424)<br><br>T2:<br>G1= 47.7 (±9.95) vs G2= 47.2 (±10.06) (P=0.912)

				Control group (G2);	Typical physical therapy (40-min) each day		Within group changes:<br>G1:<br>T0-T2: P<0.05<br>G2:<br>T0-T2: P=NS
						SF-36 - Mental health	T0:<br>G1= 51.2 (±7.28) vs G2= 48.8 (±10.87) (P=0.428)<br><br>T2:<br>G1= 53.5 (±9.29) vs G2= 56.2 (±8.48) (P=0.481)<br><br>Within group changes:<br>G1:<br>T0-T2: P=NS<br>G2:<br>T0-T2: P=NS
**Healthy older adults**							
Carral et al. (2022) [40]	12 (12)	AUTONOMYO; gait	Medical or day-care center, domestic caregiver or retirement homes<br><br>Qualitative study	Home dwelling older people	Semi-structured interviews	The psychosocial themes on the acceptance of a lower-limb exoskeleton in daily living	Older adults with reduced mobility expressed the belief that lower limb exoskeletons would enhance their autonomy, while also perceiving that their usage would alleviate the sense of burden they might impose on their support network. However, there was a degree of ambivalence among participants, influenced by their personal experiences of the aging process and their perceptions of human-machine interaction.
				Informal caregivers (data not considered)			
				Professional caregivers (data not considered)			
**Cognitive performance indicators**							
**Parkinson's Disease**							
Gryfe et al. (2022) [60]	40 (NA)	Keeogo Rehab™; powered knee assistance	Hospital<br><br>RCT<br><br>T0: Baseline measurement<br><br>T1: Post- intervention (8 weeks)	Exoskeleton exercise (Exo);<br><br>G1	8 weeks of aerobic, strength and functional mobility exercises with the exoskeleton (16 sessions, 2 times a week)	SCOPA-COG (max 43)	G1: Exo<br><br>T0: 25.7 (±4.7)<br>T1: 29.1 (±4.6)<br>T0-T1: increase of 3.4 (±3.3) (P=0.003, P significance level = 0.017))<br><br>G2: Nxo
				Non-exoskeleton exercise (Nxo);	8 weeks of aerobic, strength and functional mobility exercises without the		

				G2	exoskeleton (16 sessions, 2 times a week)		T0: 26.4 (±5.6)<br>T1: 26.9 (±6.5)<br>T0-T1: increase of 0.6 (±3.5) (P=0.552, P significance level = 0.017))<br><br>G3: Con<br><br>T0: 24.4 (±5.9)<br>T1: 25.2 (±5.5)<br>T0-T1: increase of 0.8 (±3.0) (P=0.374, P significance level = 0.017))<br><br>Between-groups comparison:<br>T0: (P=0.635)<br>T0-T1: (P=0.052)
				Wait-list control (Con);<br><br>G3	No intervention	- Memory and learning sub-scale (max 22)	G1: Exo<br><br>T0: 8.3 (±2.8)<br>T1: 11.2 (±2.4)<br>T0-T1: increase of 2.9 (±2.4) (P=0.001, P significance level = 0.017))<br><br>G2: Nxo<br><br>T0: 9.4 (±3.5)<br>T1: 9.7 (±3.3)<br>T0-T1: increase of 0.4 (±3.1) (P=0.669, P significance level = 0.017))<br><br>G3: Con<br><br>T0: 8.5 (±2.7)<br>T1: 8.5 (±3.5)<br>T0-T1: decrease of 0.1 (±2.6) (P=0.918, P significance level = 0.017))<br><br>Between-groups comparison:<br>T0: (P=0.642)<br>T0-T1: (P=0.0014)
						- Attention sub-scale (max 4)	G1: Exo<br><br>T0: 3.2 (±1.2)

							T1: 3.2 (±1.0)<br>T0-T1: decrease of 0.1 (±1.1) (P=0.808, P significance level = 0.017))<br><br>G2: Nxo<br><br>T0: 3.7 (±0.8)<br>T1: 3.3 (±1.3)<br>T0-T1: decrease of 0.4 (±0.9) (P=0.082, P significance level = 0.017))<br><br>G3: Con<br><br>T0: 3.4 (±1.1)<br>T1: 3.5 (±1.0)<br>T0-T1: increase of 0.2 (±0.8) (P=0.502, P significance level = 0.017))<br><br>Between-groups comparison:<br>T0: (P=0.490)<br>T0-T1: (P=0.384)
						- Executive functions sub-scale (max 12)	G1: Exo<br><br>T0: 9.6 (±1.7)<br>T1: 10.3 (±1.5)<br>T0-T1: increase of 0.7 (±1.2) (P=0.056, P significance level = 0.017))<br><br>G2: Nxo<br><br>T0: 9.7 (±2.1)<br>T1: 10.0 (±2.4)<br>T0-T1: increase of 0.3 (±0.9) (P=0.263, P significance level = 0.017))<br><br>G3: Con<br><br>T0: 8.6 (±2.5)<br>T1: 9.2 (±2.5)<br>T0-T1: increase of 0.6 (±1.9) (P=0.264, P significance level = 0.017))<br><br>Between-groups comparison:<br>T0: (P=0.358)

							T0-T1: (P=0.748)
						- Visuospatial functions sub-scale (max 5)	G1: Exo<br><br>T0: 4.5 (±0.5)<br>T1: 4.4 (±0.5)<br>T0-T1: decrease of 0.2 (±0.8) (P=0.502, P significance level = 0.017))<br><br>G2: Nxo<br><br>T0: 3.6 (±0.9)<br>T1: 3.9 (±0.7)<br>T0-T1: increase of 0.4 (±0.8) (P=0.136, P significance level = 0.017))<br><br>G3: Con<br><br>T0: 3.8 (±1.2)<br>T1: 3.9 (±0.9)<br>T0-T1: increase of 0.1 (±1.4) (P=0.851, P significance level = 0.017))<br><br>Between-groups comparison:<br>T0: (P=0.032)<br>T0-T1: (P=0.435)
						PDQ-39 - Cognition sub-scale (max 100)	G1:<br>T0: 25.5 (±15.4)<br>T1: 21.6 (±13.4)<br>T0-T1: decrease of 3.8 (±7.5) (P=0.088, P significance level = 0.017))<br><br>G2:<br>T0: 18.3 (±16.2)<br>T1: 24.0 (±18.2)<br>T0-T1: increase of 5.8 (±7.4) (P=0.016, P significance level = 0.017))<br><br>G3:<br>T0: 24.0 (±14.6)<br>T1: 21.2 (±14.3)<br>T0-T1: decrease of 2.9 (±11.3) (P=0.376, P significance level = 0.017))

							Between-groups comparison:<br>T0: (P=0.459)<br>T0-T1: (P=0.037)
Taki et al. (2020) [54]	72 post-matched study patients (CRP group: n = 36 and HRP group: n = 36) (NA);	HAL; gait	Hospital<br><br>Observational study	CRP (G1);	Gait training with KAFO or AFO<br><br>3 hours/day for 7 days/week	FIM - Cognitive subscore	T0: G1=15.5 (±9.3) vs G2=17.89 (±9.57) (P=0.347)<br><br>T1: G1=25.67 (±8.63) vs G2=30.5 (±5.14) (P=0.008)<br><br>T0-T1:<br>G1= increase of 10.17 (±8.45)<br>G2= increase of 12.61 (±8.7)<br>G1 vs G2 (P=0.299)
				HRP (G2);	Gait training with HAL<br><br>3 hours/day for 7 days/week		
**Sensory performance indicators ("symptom-based")**							
**Parkinson's disease**							
Gryfe et al. (2022) [60]	40 (NA)	Keeogo Rehab™; powered knee assistance	Hospital<br><br>RCT<br><br>T0: Baseline measurement<br><br>T1: Post- intervention (8 weeks)	Exoskeleton exercise (G1)	8 weeks of aerobic, strength and functional mobility exercises with the exoskeleton (16 sessions, 2 times a week)	PDQ-39 - Bodily discomfort sub-scale (max 100)	G1:<br>T0: 24.4 (±16.1)<br>T1: 24.4 (±20.0)<br>T0-T1: change of 0.0 (±13.6) (P=1.000, P significance level = 0.017))<br><br>G2:<br>T0: 34.6 (±24.0)<br>T1: 27.6 (±17.5)<br>T0-T1: decrease of 7.1 (±13.1) (P=0.076, P significance level = 0.017))<br><br>G3:<br>T0: 26.9 (±18.7)<br>T1: 28.8 (±20.3)<br>T0-T1: increase of 1.9 (±12.3) (P=0.584, P significance level = 0.017))<br><br>Between-groups comparison:<br>T0: (P=0.401)<br>T0-T1: (P=0.351)
				Non-exoskeleton exercise (G2)	8 weeks of aerobic, strength and functional mobility exercises without the exoskeleton (16 sessions, 2 times a week)		
				Wait-list control (G3)	No intervention	PDQ-39 - Communication sub-scale (max 100)	G1:<br>T0: 30.1 (±23.9)<br>T1: 21.8 (±21.1)

							T0-T1: decrease of 8.3 (±11.3) (P=0.021, P significance level = 0.017))<br><br>G2:<br>T0: 15.4 (±19.5)<br>T1: 19.9 (±18.2)<br>T0-T1: increase of 4.5 (±14.7) (P=0.292, P significance level = 0.017))<br><br>G3:<br>T0: 17.9 (±14.8)<br>T1: 24.4 (±23.2)<br>T0-T1: increase of 6.4 (±16.0) (P=0.175, P significance level = 0.017))<br><br>Between-groups comparison:<br>T0: (P=0.142)<br>T0-T1: (P=0.083)
**Osteoarthritis:**							
Koseki et al. (2021) [42]	21 (NA)	Honda Walking Assist; assist hip flexion and extension	Hospital<br><br>Quasi-experimental design;<br><br>T0: Pre- TKA measurement<br><br>T1: Week 2 following TKA<br><br>T2: Week 4 following TKA<br><br>T3: Week 8 following TKA	HWA group (G1);	Conventional rehabilitation + HWA gait training from week 1 to 5 after TKA<br><br>Total of 17-20 gait training sessions (each around 20-min), with 4-5 sessions per week	WOMAC-p	T0:<br>G1= 45.91 (±19.34) vs G2= 60.91 (±21.89) (P=0.104, d= 0.73)<br><br>T1:<br>G1= 64.66 (±18.17) vs G2= 62.73 (±16.18) (P=0.795, d=0.11)<br><br>T2:<br>G1= 74.55 (±18.09) vs G2= 79.09 (±14.29) (P=0.521, d=0.28)<br><br>T3:<br>G1= 80.00 (±13.42) vs G2= 84.95 (±11.70) (P=0.367, d=0.39)
				Control group (G2);	Conventional rehabilitation		
Setoguchi et al. (2022) [53]	47 (NA)	HAL;	Hospital<br><br>RCT<br><br>T0: Preoperatively	HAL group (G1);	HAL gait training: 3 sessions per week (40-min each) for 6 weeks in total + typical physical therapy on days without HAL training	Harris hip score – pain	T0:<br>G1= 17.5 (±8.86) vs G2= 22.5 (±7.07) (P=0.221)<br><br>T2:

			T1: 1 week postoperatively<br><br>T2: 3 weeks postoperatively	Control group (G2);	Typical physical therapy (40-min) each day	 <br>Harris hip score - Bodily pain	G1= 40.3 (±4.59) vs G2= 42.5 (±2.07) (P=0.263)<br><br>Within group changes:<br>G1:<br>T0-T2: P<0.05<br>G2:<br>T0-T2: P<0.05<br><br>T0:<br>G1= 33.9 (±8.14) vs G2= 28.3 (±6.11) (P=0.150)<br>T2:<br>G1= 52.2 (±12.06) vs G2= 48.9 (±8.09) (P=0.392)<br>Within group changes:<br>G1:<br>T0-T2: P<0.05<br>G2:<br>T0-T2: P<0.05
Yoshikawa et al. (2018) [57]	19 (NA)	HAL; Gait	Hospital<br><br>Quasi-experimental design;<br><br>T0: Pre- TKA measurement<br>T1: Week 1 following TKA<br>T2: Week 2 following TKA<br>T3: Week 3 following TKA<br>T4: Week 4 following TKA<br>T5: Week 8 following TKA	HAL group (G1);	10-12 HAL training sessions (around 15-min each) over a 4 week period + conventional physical therapy (60-80 min a day)	WOMAC-p	T0:<br>G1= 72.0 (±13.4) vs G2= 60.0 (±23.2) (P=0.169)<br>T2:<br>G1= 78.0 (±15.7) vs G2= 59.1 (±18.4) (P=0.021)<br>T4:<br>G1= 79.0 (±12.4) vs G2= 80.0 (±13.6) (P=0.863)<br>T5:<br>G1= 88.0 (±5.7) vs G2= 79.2 (±14.6) (P=0.218)
				Control group (G2);	Conventional physical therapy (60-120 min a day)		

Data in the results section are Mean (±SD), unless explicitly mentioned otherwise. P-level of significance = 0.05, unless explicitly mentioned otherwise.

N=number of participants; NA=information not available; NS= Not Significant; RCT: Randomized Controlled Trial; ES= effect size; CI= confidence interval; SD= Standard Deviation; PD: Parkinson's Disease; 10MWT=10 Meter Walk Test; SMA= Stride Management Assist exoskeleton; SEM= Standard Error of the Mean; HAL: Hybrid Assistive Limb exoskeleton; ROM= Range of Motion; HWA= Honda Walking Assist exoskeleton; EGT= Ekso Gait Training, OGT=Conventional Gait Training; sEMG= surface electromyography; CG= Control Group; EG= Experimental Group; KAFO= Knee-ankle-foot orthosis; AFO= Ankle-foot orthosis; CRP= Conventional Gait Training; HRP= HAL Rehabilitation Program; Br-stage= Brunnstrom Stages of Stroke Recovery; IQR= interquartile range; CPT-G= conventional physical therapy and gait training; ICT-C= interlimb coordinated humanoid robot combined with conventional physical therapy; MAS= modified Ashworth scale; EMG= electromyography; SCAR: Swing-Controlled Ankle Robot; PAAR: Power-Assisted Ankle Robot; CT: Conventional Training; Gems-H= Gait Enhancing and Motivating System for Hip; Gems= Gait Enhancing Mechatronic System; BW= Body Weight; iEMG= integrated electromyography; VAS= visual analog scale; EX1= wearable

robotic hip exoskeleton; RPE= rating of perceived exertion; Borg CR= Borg Category Ratio scale; SF-36= 36-Item Short Form Health Survey; RATW= robot-assisted treadmill walking; PAFO= powered ankle-foot orthoses; RAG= robot-assisted gait; Eem= aerobic energy expenditure measurement; APO= active pelvis orthosis; UPDRS= Unified Parkinson's Disease Rating Scale; PDQ-39= Parkinson's Disease Questionnaire-39; HADS= Hospital Anxiety and Depression Scale; SCOPA-COG= Scales for Outcomes in Parkinson's Disease-COGnition; FIM= Functional Independence Measure; WOMAC-p= Western Ontario and McMaster Universities Osteoarthritis Index – pain subscale; TKA=total knee arthroplasty
